# Best practices in ‘0 K’ DFT energy calculations on mol­ecular crystal structures

**DOI:** 10.1107/S2053229626006169

**Published:** 2026-07-08

**Authors:** Jacco van de Streek, Erin R. Johnson

**Affiliations:** a Avant-garde Materials Simulation, Alte Strasse 2, Merzhausen, 79249, Germany; b Department of Chemistry, Dalhousie University, 6243 Alumni Crescent, Halifax, NS, B3H 4R2, Canada; c Yusuf Hamied Department of Chemistry, University of Cambridge, Lensfield Road, Cambridge, CB1 1EW, United Kingdom; University of Strathclyde, United Kingdom

**Keywords:** energy calculations, zero Kelvin, density functional theory, DFT, energy minimization, best practice

## Abstract

This article describes best practices for ‘zero Kelvin’ energy calculations on mol­ecular crystals and is targeted for non-experts who would like to perform their own calculations or assess the accuracy and validity of such calculations reported in the scientific literature. The recommended com­putational procedure can be summarized as follows. (1) Energy optimize the crystal structures of inter­est with a suitable dispersion-corrected density functional, such as PBE (abbreviated here as PBE+D). (2) Based on the extent of geometric distortions after energy minimization, decide if subsequent energy calculations are required and meaningful. (3) Decide if the PBE+D crystal energies suffice; if they do not, perform a single-point energy calculation on the mol­ecular crystal with a hybrid exchange-correlation functional, such as PBE0, again with a suitable dispersion correction. Similarly, decide if single-point energy calculations on the individual mol­ecules with a more accurate level of theory, such as MP2D or ωB97X+D, are required to im­prove the mol­ecular energies to account for multiple conformations or tautomers.

## Abstract 2

This article describes best practices for ‘zero Kelvin’ energy calculations on mol­ecular crystals and is targeted for non-experts who would like to perform their own calculations or assess the accuracy and validity of such calculations reported in the scientific literature.

## Introduction

Density functional theory (DFT) calculations are now a routinely used technology in applications to mol­ecular crystals, providing valuable predictions of their structures, relative energies and other properties of inter­est. DFT calculations can be used for many purposes where experimental data are cur­rently not available or cannot be ob­tained directly, or where there are reasons to suspect that the experimental crystal structure model may be incorrect. For example, the results from a DFT energy minimization can be used to confirm if an experimental crystal structure is stable, or to decide between multiple models dif­fering in H-atom positions depending on their relative energies.

As hardware becomes cheaper and faster, and software becomes more efficient, increasingly many non-experts will wonder how they can use DFT calculations in their work, find themselves collaborating with com­pu­ta­tional crys­tal­log­raph­ers, or review papers reporting DFT calculations. In this article, we provide an overview of best practices and guiding principles, based on a collection of our own experiences collected over the past 30 years, the scientific literature, discussions with many experts in the field, and questions from non-experts.

As the field of DFT calculations is a vast domain, the hardest part of writing a best practices guide for a general audience is the decision of what to include. For simplicity, this article is restricted to closed-shell mol­ecular crystal structures in their ground state in thermodynamic equilibrium that are insulators. In practice, this spans most crystal structures con­sisting of the elements C and H, and any of B, N, O, P, S, Se, Si, F, Cl, Br and I, as well their ions (*R*—COO^−^, *R*—NH_3_^+^, Cl^−^, *etc*.), and the Na^+^, K^+^ and Ca^2+^ cations. Transition metals (*e.g.* Cu^2+^, Fe^2+^), which may have unpaired electrons, and any materials that are conductors (such as metals), require special treatment. The crystals are assumed to be ideal: there are no defects and the crystals extend to infinity in all directions. Temperature-dependent properties (*i.e*. free energies and phonons) will not be dis­cus­sed, and neither will any calculation requiring the search for a global minimum (*e.g.* crystal structure prediction or conformer analysis). Energies will only be com­pared between systems having the same tem­per­a­ture, pressure and chemical com­position. Only energy calculations for crystal structures and isolated mol­ecules in a vacuum will be addressed.

As DFT calculations on mol­ecular crystals begin with relaxation of the structures of inter­est, ‘0 K’ energy minimization is described in detail. Electronic energies, even if they are not proper tem­per­a­ture-dependent free energies, can reveal trends and distinguish between com­peting models. Thus, single-point calculations on crystal structures and individual mol­ecules, which are required to im­prove the calculated electronic energies after the energy minimization, are also dis­cus­sed in detail. In brief, the best practices for 0 K energy calculations on mol­ecular crystals can be summarized as: energy minimize the crystal structure with a lower-level (cheap) DFT method, then run a single-point calculation with a higher-level (more expensive) method on the crystal structure and, if required, also on the isolated mol­ecule(s). Recommendations regarding the best choices of methods for each of these com­putational steps are provided. Perhaps more important than describing how to run DFT energy calculations is to explain *why* it makes sense to perform them that way. Hence, a list of guiding principles that should be considered when deciding how to apply DFT to a research question is also included. The article ends with some examples of crystallographic cases from the literature where DFT calculations could have been applied.

## Best practices for DFT calculations

### Overview

To avoid losing the main message in the details, a high-level overview of a recommended com­putational workflow is described in detail below, including the individual steps and their justifications.

(1) Energy optimize the crystal structures of inter­est with the Perdew–Burke–Ernzerhof or PBE functional (Perdew *et al.*, 1996*a* ▸) paired with a dispersion correction (PBE+D) or another similarly reliable generalized gradient approximation (GGA). This provides a good balance between com­putational cost and accuracy, as PBE+D calculations are faster, but less accurate, than more sophisticated DFT methods. Except for certain very specific use cases, the unit cell should be fully optimized as part of the energy minimization.

(2) Based on the distortions after energy minimization, decide if subsequent energy calculations are required and meaningful. If the unit cell was kept fixed, a subsequent energy minimization with the unit cell allowed to fully relax (starting from the result of the energy minimization with the fixed unit cell) is probably required.

(3*a*) Decide if the PBE+D crystal energies suffice. Even if the PBE+D energies are deemed accurate enough, a subsequent single-point calculation, possibly with a larger basis set, larger *k*-point grid, or in the space group *P*1, can be used to detect possible problems with any of the numerical approximations in the calculation.

(3*b*) If the PBE+D crystal energies do not suffice, add a single-point crystal energy calculation with a hybrid exchange-correlation functional, such as PBE0 (Adamo & Barone, 1999 ▸), with a dispersion correction.

(3*c*) Decide if single-point energy calculations on the individual mol­ecules, with higher levels of theory such as MP2D or ωB97X+D, are required to im­prove the mol­ecular energies.

### Density functional theory (DFT)

A very brief overview of DFT is given here with the aim to introduce some of the terminology and levels of approximation. In DFT, the energy *E* is written as a functional of the electron density ρ(*x*,*y*,*z*): *E* = *E*[ρ(*x*,*y*,*z*)]. *E* can be written as a sum of several contributions, all of which are exact except for two: the exchange functional and the correlation functional, for which only approximations are known. The better the approximation, the longer the calculation typically takes. The challenge when applying DFT is, therefore, the choice of an appropriate level of approximation.

DFT functionals can be divided into roughly four levels:

(1) Local density approximation (LDA) functionals.

(2) Generalized gradient approximation (GGA) functionals, such as PBE.

(3) Hybrid functionals, such as PBE0 (pronounced ‘p b e zero’). In hybrid functionals, the approximated exchange energy is im­proved by mixing in some amount of ‘exact exchange’ from Hartree–Fock theory. In global hybrid functionals, the extent of this exact exchange is a constant, usually 25% as argued based on physical grounds (Perdew *et al.*, 1996*b* ▸), although mixing fractions of *ca* 15 to 55% may be used depending on the particular choice of functional. Alternatively, some hybrid functionals (HSE06, ωB97X, *etc*.) are range-separated, meaning that the extent of exact-exchange mixing varies depending on the electron–electron distance.

(4) More sophisticated or specialized functionals, including double-hybrid and local-hybrid functionals, which will not be dis­cus­sed further here.

As the approximations made in their construction have proven to give both qualitatively and qu­anti­tatively poor results, LDA functionals should not be used in general.

The PBE functional is a GGA that is available in most DFT packages, requires relatively modest com­putational resources (com­puter time and memory) and is appropriate for the energy minimization of most mol­ecular crystals. PBE was the basis for the crystal structures and energies of the first four-out-of-four success rate in the 2007 crystal structure prediction blind test (Neumann *et al.*, 2008 ▸; Neumann & Perrin, 2005 ▸). Throughout this article, we write the PBE functional whenever we refer to a GGA functional because PBE is such a familiar name and it is con­sistently implemented in all common DFT codes. However, most GGA functionals are at least as good a choice and we have been very satisfied with the performance of the PBE, PW91 (Wang & Perdew, 1991 ▸), revPBE (Zhang & Yang, 1998 ▸) and B86bPBE (Becke, 1986 ▸) functionals paired with appropriate dispersion corrections.

Like all GGA functionals, PBE has several well-known shortcomings:

(1) It very severely underestimates the strength of attractive van der Waals inter­actions (London dispersion) (Johnson *et al.*, 2004 ▸).

(2) It overestimates the strengths of hy­dro­gen bonds and halogen bonds (Gillan *et al.*, 2016 ▸; Kim *et al.*, 2019 ▸).

(3) It overestimates the stability and degree of planarity of conjugated systems (Nam *et al.*, 2021 ▸).

(4) It overestimates the stability of organic salts over acid–base cocrystals (favours proton jumps) (LeBlanc *et al.*, 2018 ▸).

(5) It gives relatively poor accuracy for the energies of breaking and forming bonds (Goerigk *et al.*, 2017 ▸).

Points 2 to 4 are all manifestations of delocalization error (Bryenton *et al.*, 2023 ▸). In practice, pragmatic solutions for over­coming these shortcomings are known for many applications.

Ad 1. The failure of most density functionals to capture the weak attractive London dispersion forces between mol­ecules is a well-researched topic and, fortunately, an excellent solution exists in the form of dispersion corrections. There are now many available dispersion corrections from which to choose; some of the more popular ones are D2 (Grimme, 2006 ▸), D3 (Grimme *et al.*, 2010 ▸), D3(BJ) (D3 with Becke–Johnson damping; Grimme *et al.*, 2011 ▸; Johnson & Becke, 2006 ▸), D4 (Caldeweyher *et al.*, 2020 ▸), XDM (exchange–hole dipole moment model; Becke & Johnson, 2007 ▸; Otero-de-la-Roza & Johnson, 2012*b* ▸), TS (for Tkatchenko and Scheffler; Tkatchenko & Scheffler, 2009 ▸) and MBD or MBD-nl (many-body dispersion and non-local many body dispersion; Tkatchenko *et al.*, 2012 ▸). The dispersion correction is appended to the functional (*i.e.* PBE+D3, PBE+MBD-nl, *etc*.). We have more than once observed that experimental mol­ecular crystal structures energy minimized with PBE+D2 contracted by 8 to 12%, while none of the other dispersion corrections show this contraction, and recommend against the use of D2 for mol­ecular crystal structures. In our experience, the newer MBD correction performs much better than the TS correction by the same authors, so we similarly do not recommend TS. We have used Neumann–Perrin (Neumann & Perrin, 2005 ▸), D3, D3(BJ), XDM and MBD-nl in our publications and, for the reproduction of experimental crystal structures, their performance is excellent. In this article, we will use the notation ‘+D’ to indicate any of these five recommended dispersion methods.

Ad 2. The effect of overstabilizing hy­dro­gen (and halogen) bonds on calculated crystal structures is hardly noticeable because 0 K energy minimizations already cause the unit cell to contract slightly relative to experimental single-crystal structures ob­tained at finite tem­per­a­tures. Additionally, strong hy­dro­gen bonds tend to be persistent across polymorphs, leading to significant error com­pensation; if two polymorphs have dif­ferent hy­dro­gen-bonding patterns, the error com­pensation will be reduced. If energy dif­ferences are important, single-point calculations with the more accurate PBE0+D functional can be performed at the PBE+D geometries to im­prove the predicted energetics (Hoja *et al.*, 2019 ▸). Observing that two polymorphs have dif­ferent hy­dro­gen-bonding patterns, for example, is a good reason to assume that PBE0+D single-point energy calculations are needed to im­prove the calculated energies. While PBE0 uses 25% exact exchange, alternative hybrid functionals including 50% exact-exchange mixing can sometimes be more reliable for systems with strong hy­dro­gen bonding and/or halogen bonding due to mitigation of delocalization error, although they are less accurate in general (Goerigk *et al.*, 2017 ▸) and should therefore be em­ployed with care.

Ad 3. Delocalization error is perhaps the most problematic shortcoming of the PBE functional and its effect on mol­ecular conformational energies can be difficult to predict and detect. The typical manifestation is that a more-planar conformer with extended conjugation is overstabilized relative to a less-planar conformer that may form more favourable inter­molecular inter­actions (Greenwell & Beran, 2020 ▸). In practice, a single-point correction on the isolated mol­ecules with MP2D or ωB97X+D [see Beran (2025 ▸) and below] is an adequate solution, but requires relatively large com­putational resources, regularly for little gain in accuracy. However, there are some instances where neglect of delocalization error on conformational energies can be catastrophic in terms of polymorph ranking.

Ad 4. Accurately predicting the relative energies of crystals related by proton transfer remains an outstanding challenge for DFT. Unlike points 2 and 3, this manifestation of delocalization error cannot be addressed in a con­sistently reliable way even with hybrid functionals, and single-point energy calculations will not resolve the situation as the error may alter the proton positions during initial energy minimization. The special case of salt *versus* cocrystal will be dis­cus­sed further in Section 6.4.

Ad 5. PBE and other GGA functionals are well known to overestimate covalent bond strengths, and this was the original motivation for the development of hybrid functionals (Becke, 1993 ▸). However, this error will not affect mol­ecular crystal geometries significantly. In practice, it is recommended that users only com­pare DFT energies between crystal structures containing the same chemical com­pounds for maximum error cancellation. If energies must be com­pared for two or more tautomeric forms of a com­pound, accuracy can be im­proved using PBE0+D and/or single-mol­ecule corrections from ωB97X+D or MP2D.

Even today, the PBE functional still offers an adequate accuracy *versus* speed com­promise for energy minimization, *i.e.* for reproducing crystal *structures*, but has been proven to give incorrect *energy* rankings for experimental polymorphs of several com­pounds. Even in the 0 K approximation, crystal energies are better calculated with a hybrid functional. For 0 K energies of mol­ecular crystal structures, a single-point calculation with the PBE0+D (Adamo & Barone, 1999 ▸) or B86bPBE0+D (Price *et al.*, 2023 ▸) hybrid functional offers an excellent accuracy *versus* speed com­promise.

### Basis sets

In a DFT calculation, the electron density, ρ(*x*,*y*,*z*), is approximated as a series expansion in a set of functions called basis functions. *CASTEP*, *Quantum ESPRESSO* and *VASP* are plane-wave codes, which means that they use a Fourier series to model the electron density, while *FHI-aims* uses numerical atom-centred basis functions. Both of these basis-set types are capable of excellent accuracy if sufficiently many functions are included. An exact representation of the electron density would require an infinite number of basis functions, and truncating the expansion to a finite number of functions introduces numerical errors. The more basis functions, *i.e*. the more degrees of freedom, the lower the energy. Only energies calculated with the same basis-set size should be com­pared or used to com­pute the relative energies of dif­ferent crystal structures.

The size of the basis set can be viewed as the number of mathematical functions per Å^3^, such that the basis-set size increases, and the energy decreases, when the unit-cell volume shrinks. The resulting driving force is called the Pulay stress or Pulay pressure. The smaller the basis set, the greater the Pulay pressure. Furthermore, the number of plane waves, the cut-off energy and the unit-cell volume are related, *i.e.* when the unit-cell volume changes either the cut-off energy or the number of plane waves must change accordingly. Because the number of plane waves is a discrete number, this may lead to sudden small jumps in the energy, which confuses the minimization algorithm. Such jumps can be prevented by keeping the number of plane waves constant, but then the basis set changes while the unit-cell volume changes and the final crystal energy no longer corresponds to the initially specified plane-wave cut-off energy. If the unit-cell volume changes significantly during the energy minimization, it may therefore be necessary to restart the energy minimization using the energy-minimized structure as the starting structure. To check if this is necessary, calculate a single-point energy on the energy-minimized crystal structure with the same settings used for the energy minimization; the energy ob­tained from the single-point calculation should be the same as the final energy of the energy minimization within numerical accuracy, *i.e.* |Δ*E*| < 0.05 kcal mol^−1^.

For energy minimizations on an individual crystal structure, or a small set of structures, a plane-wave basis set with a minimum cut-off energy of 800 eV (electron volts) is recommended. For the purposes described in this article, values over 1200 eV cannot be expected to lead to better results and should not be used. Conversely, for energy minimizations with the unit cell fixed, Pulay pressure does not play a role and such minimizations can be carried out reliably with a relatively low cut-off energy of 520 eV. One can pre-optimize the crystal structure with the unit-cell parameters fixed with a smaller cut-off energy. That energy-minimized structure can then be used as the starting point for a subsequent energy minimization, with a larger cut-off energy, in which the unit-cell parameters are allowed to relax as well. For the particular application of mol­ecular crystal structure prediction (CSP), this cut-off energy of 520 eV is often used as a com­promise between speed and accuracy since CSP requires the DFT energy minimization of possibly hundreds of crystal structures. CSP studies benefit substanti­ally from error cancellation and single-point calculations with a larger basis set can be used to im­prove the energies, while the effect of tem­per­a­ture on the energy dif­ferences is greater than the error introduced by using a relatively small basis set for energy minimization. Note that the cut-off energy is sometimes given in Ry (Rydberg); 520 eV corresponds to about 40 Ry, 800 eV to about 60 Ry and 1200 eV to about 90 Ry. These values are appropriate for use with either ultrasoft or PAW pseudopotentials (see Section 2.5), which are generally recommended for mol­ecular crystals; in contrast, norm-conserving potentials tend to require higher plane-wave cut-offs.

In *FHI-aims*, the available numerical atomic orbital (NAO) basis sets are referred to as ‘light’, ‘lightdense’, ‘lightdenser’, ‘inter­mediate’ and ‘tight’. The lightdense or lightdenser basis set is a good com­promise for energy minimization, while a single-point calculation with the tight basis set on the final energy-minimized structure is recommended to im­prove the energy. The use of NAOs makes calculations with hybrid functionals such as PBE0 feasible for mol­ecular crystals, while the com­putational cost of such calculations can be prohibitive with plane-wave basis sets. Even greater efficiency can be achieved using the PBE0′ (pronounced ‘p b e zero prime’) implementation in *FHI-aims*, which makes use of range separation (Kokott *et al.*, 2025 ▸).

Finally, the class of Gaussian basis sets is by far the most widely used for DFT calculations on isolated mol­ecules. Here, collections of atom-centred Gaussian functions are used to construct the orbitals and electron density. Calculations on mol­ecular crystals with Gaussian basis sets are possible, for example, using the *CRYSTAL17* code (Dovesi *et al.*, 2018 ▸). However, small basis sets such as 6-31G* (pronounced ‘six three one g star’) suffer from substantial basis-set incom­pleteness error, resulting in a large Pulay pressure and unit-cell contraction. Use of larger sets, such as 6-31+G*, im­proves accuracy, but leads to linear dependencies and often inhibits self-con­sistent-field convergence of the DFT energies. Additionally, time-consuming counterpoise corrections are still needed to ob­tain reasonable energy rankings. Thus, Gaussian basis sets are ultimately not recommended for calculations on mol­ecular crystals.

### *k*-points

As a consequence of periodic boundary conditions, DFT calculations on mol­ecular crystals involve integration over a set of ‘*k*-points’ that span the unit cell in reciprocal (mo­men­tum) space, termed the Brillouin zone. The finer the integer grid of *k*-points, the more accurate the numerical integration, but the more costly the calculation. In general, we recommend a *k*-point spacing of 0.07 Å^−1^ or better to calculate reliable energies. Additionally, Neumann & Perrin (2005 ▸) recommend a minimum of two *k*-points in each reciprocal unit-cell direction. When com­paring the energies of two closely related crystal structures, for example, a low-tem­per­a­ture and a high-tem­per­a­ture form of the same com­pound with very similar unit cells, one might consider ensuring that the same set of *k*-points is used for maximum error com­pensation.

To check if there are any problems with the number of *k*-points, a single-point energy calculation can be performed on the crystal structure (either before or after energy minimization) with and without a minimum of two *k*-points in each reciprocal unit-cell direction. The energies should be the same within numerical accuracy, *i.e.* |Δ*E*| < 0.05 kcal mol^−1^. Similarly, to check if there are any problems with the *k*-point generation caused by the space-group symmetry, one can perform a single-point energy calculation with the same settings on the crystal structure converted to the *P*1 space group. Finally, because the set of *k*-points depends on the reciprocal unit cell, it is also possible that the number of *k*-points required after energy minimization is dif­ferent from the initial set. It may therefore be necessary to restart the minimization with an updated starting structure if recalculating the single-point energy of the energy-minimized structure with an updated *k*-grid does not give the same result as the final energy of the minimization to within numerical accuracy.

### Core electrons and relativistic effects

The electrons in an atom can be divided into the valence electrons, which determine the chemistry (as well as the structures and energies of most mol­ecular crystals), and the core electrons. The properties of the core electrons depend almost exclusively on the element and are otherwise relatively constant across chemical com­pounds. Moreover, for the purpose of DFT energy calculations, the core electrons are much more difficult to model than the valence electrons (particularly with plane-wave basis sets) due to the need to accurately describe the nuclear ‘cusps’. This has led to two approaches to deal with the core electrons: (i) model them explicitly or (ii) precalculate their electron-density contributions and store them in a file. The first option is implemented in ‘all-electron’ codes such as *FHI-aims*, while the second option is standard in plane-wave codes such as *CASTEP*, *Quantum ESPRESSO* and *VASP*, and is referred to by names such as the ‘pseudopotential’ or ‘effective core potential’ approach.

A pseudopotential can be thought of as a sphere centred at the nucleus; the electron density inside the sphere is precom­puted, while the electrons outside the sphere are treated explicitly. The radius of the sphere can be varied, and the extent to which the core electrons are treated explicitly is referred to as ‘soft’ or ‘hard’. The harder the pseudopotential, the smaller the sphere and the more of the explicit character of the core electrons is preserved. When the core electrons are not treated explicitly, the nucleus and its core electrons can be considered as a unit to which the valence electrons are added. In that picture, the nucleus plus its core electrons is sometimes referred to as an ‘ion’. Using that terminology, a paracetamol mol­ecule, for example, con­sists of 20 ions plus the valence electrons.

Several types of pseudopotentials are commonly used, including frozen-core potentials, norm-conserving pseudopotentials, ultrasoft core potentials and PAW (Projector-Augmented Wave) potentials. The latter two categories are used in the vast majority of com­putations on mol­ecular crystals, for which the default settings or recommendations of the particular DFT software are usually sufficient. GIPAW (Gauge-Including Projector Augmented Wave) potentials are required for solid-state NMR calculations, in which the core electrons play a prominent role, although this is beyond the scope of the present article.

The closer the electrons are to the nucleus and the heavier the element, the greater the effect of relativistic corrections. For the light elements that form most organic mol­ecules, relativistic corrections are unnecessary for the reproduction of crystal structures or for energy dif­ferences. However, such corrections are needed for heavier elements, such as Br or I atoms. In plane-wave DFT calculations (*CASTEP*, *Quantum ESPRESSO*, *VASP*, *etc*.), scalar relativistic effects are in­clu­ded automatically through the pseudopotential. However, in all-electron codes, such as *FHI-aims*, an explicit relativistic correction must be used. Specifically, when an element heavier than Ca (atomic number 20) is present, *FHI-aims* forces the use of the ZORA (for Zeroth-Order Regular Approximation) relativistic correction. Because energies can only be com­pared if the treatment of relativistic effects is con­sistent, and because the additional com­putational resources required are negligible, it is strongly recommended to always use ZORA in *FHI-aims* (include ‘relativistic atomic_zora scalar’ in the control.in input file).

### Energy definition and single-point calculations

There are many specific qu­anti­ties to which the vague general term ‘energy’ can refer. The energy that is routinely reported by quantum-mechanical (QM) pro­grams is the ‘electronic energy’, which we write as *E* throughout this article. Whereas qu­anti­ties like tem­per­a­ture, volume and mass have a natural zero point, energies do not, meaning that only energy *dif­ferences* can be determined. Hence, the electronic energy reported by QM software is the electronic energy with respect to a fixed, but arbitrary, reference point, which is most often the energy of the electrons and atomic nuclei at infinite separations. The formation of mol­ecules from separated electrons and nuclei always releases large amounts of energy, so all mol­ecules and crystal structures have large negative electronic energies, with their magnitude proportional to the number of electrons in the system. Calculating the electronic energy of a crystal structure as is, without any changes to atomic coordinates or the unit cell, is referred to as a single-point energy or a single-point calculation. An energy minimization is made up of a series of single-point calculations.

It is also possible to calculate enthalpies and Gibbs or Helmholtz ‘free energies’ with quantum-mechanical software, but these are much harder to com­pute and will not be dis­cus­sed further here. Because of the missing tem­per­a­ture-dependent contributions, electronic energies are often refer­red to as ‘0 K’ (zero Kelvin) energies; however, experimental energies at 0 K include the zero-point energy (ZPE), which is not included in the electronic energy. As a result, even electronic energy dif­ferences ob­tained from QM software cannot be directly com­pared to relative experimental energies, because no tem­per­a­ture-dependent contributions and no zero-point energies are included.

The electronic energy is the energy of the entire unit cell, and so is a combination of the intra- and inter­molecular inter­actions. The ‘crystal energy’ is this electronic energy normalized per mole of mol­ecules (*i.e.* to kcal mol^−1^ or kJ mol^−1^). The ‘lattice energy’ can be calculated from the crystal energy by subtracting the individual isolated mol­ecular energies: 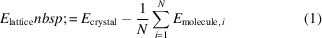
where the sum runs over all *N* mol­ecules in the unit cell.

In *FHI-aims*, which uses atom-centred basis sets, the energy of an isolated mol­ecule can be calculated directly. However, in plane-wave codes such as *CASTEP*, *Quantum Espresso* and *VASP*, calculations can only be performed on periodic structures. Thus, the energy of an isolated mol­ecule can only be calculated by embedding it in a vacuum by defining a con­trived crystal structure (in *P*1 symmetry) with very large unit-cell parameters to reduce the inter­action between periodic copies. Alternatively, a series of calculations using increasingly larger unit cells can be conducted, and the energy of the mol­ecule in an infinitely large unit cell can be ob­tained by extrapolation. If the isolated mol­ecule has a net charge, this must be set in the input file for the calculation to ensure that the number of electrons is correct. For periodic-boundary calculations, a com­pensating background charge is automatically added for charged mol­ecules to prevent divergence of the electrostatic com­ponent of the energy.

Mol­ecular energies from single-point energy calculations in vacuum, using mol­ecular geometries taken from a crystal structure. can therefore be used to calculate the inter­molecular com­ponent of the lattice energy as per Equation (1). This approximation assumes rigid mol­ecules and can be useful to identify trends for flexible mol­ecules. However, the mol­ecular energies should properly take into account the relaxation of the mol­ecule upon being excised from the crystal structure (due to the accom­panying loss of packing forces), and the fact that the most favourable mol­ecular conformation may be com­pletely dif­ferent from that found in the crystal (Thompson & Day, 2014 ▸). A simple example of this is oxalic acid, in which the proton positions will dif­fer between the crystal and the gas-phase mol­ecule to ensure stronger intra­molecular hy­dro­gen bonding in the latter case. For more sophisticated energy calculations on individual mol­ecules, the articles by Bursch *et al.* (2022 ▸) and Chattopadhyay *et al.* (2025 ▸), and references therein, are good starting points.

### Energy minimization

‘Energy minimization’ refers to a local minimization of the electronic energy with respect to changes in atomic positions and lattice parameters (unit-cell lengths and angles), *i.e.* to the nearest local minimum on a very high-dimensional potential energy surface. Note that the energy minimization cannot break symmetry to lower the energy if space-group symmetry is imposed, meaning that it may be possible to geometry optimize to ‘saddle points’ instead of local minima in rare cases. It is also possible that the crystal structure has higher symmetry after energy minimization (Hempler *et al.*, 2017 ▸).

The energy minimization process con­sists of a series of single-point calculations (as well as evaluation of the forces and stresses) on increasingly low-energy crystal structure models until some specified convergence criteria are satisfied and the optimization stops. As this process requires several, generally dozens, of successive single-point calculations, it should therefore use a relatively cheap DFT method. ‘Geom­etry optimization’ or ‘geometry relaxation’ are common terms used as alternatives for energy minimization. While energy minimization is sometimes referred to as ‘geometry minimization’, this is incorrect as the atomic coordinates are not made smaller.

Energy minimization ensures that experimental crystal structures (from any of single-crystal, powder diffraction, X-ray, neutron or electron diffraction data), manually created alternative crystal structure models (in case of ambiguities in the experimental data) and *in silico* crystal structures from CSP studies are all treated on an equal footing. This is an example of the Anna Karenina principle: there exists only one unique energy-minimized crystal structure model (with a particular DFT method and basis) for a given polymorph, but an infinite number of models that do not correspond to the energy-minimized crystal structure, each with its own crystal energy (Fig. S1 in the supporting information). Checking if two crystal structures correspond to the same polymorph (at 0 K) is also facilitated by energy minimization (Sacchi *et al.*, 2020 ▸).

The influence of omitting parts of the geometry optimization protocol on the crystal energy is shown in Table 1 ▸ for axitinib Form XLI determined at room tem­per­a­ture [Cambridge Structural Database (CSD) (Groom *et al.*, 2016 ▸) refcode VUSDIX04; Campeta *et al.*, 2010 ▸]. The average energy dif­ference between any two of the five known axitinib polymorphs (0.85 kcal mol^−1^) is included for com­parison. The shortened *X*—H bond lengths present in the experimental single-crystal X-ray structure account for *ca* 300 kcal mol^−1^ of destabilization; obviously, this value depends on the number of *X*—H bonds and, for paracetamol (CSD refcode HXA­CAN07; Nichols & Frampton, 1998 ▸), the corresponding de­stabilization is ‘only’ 69.7 kcal mol^−1^. Even after correcting the short *X*—H distances, energy minimization lowers the energy by *ca* 10 kcal mol^−1^, an order of magnitude more than the average energy dif­ference between two polymorphs. In short, in order for the com­parison of calculated energy dif­ferences to be meaningful, the crystal structure models must be energy minimized first.

Energy minimization also offers the opportunity to assess how well the DFT method reproduces the input crystal structure model, identifying possible issues with the proposed crystal structure, or with the choice of functional or basis set, before progressing to single-point energy calculations with higher levels of theory. If the crystal structure distorts substanti­ally during the relaxation, it is likely incorrect and there is no value in performing additional single-point calculations. In approximate order of importance, experimental structures can distort during energy minimization for the following reasons.

(1) Incorrectly positioned or missing H atoms. Due to their low X-ray scattering power, H atoms are sometimes positioned incorrectly, even in single-crystal X-ray structures. Incorrect models contain large inter­nal strains and stresses that can trivially be detected by relaxing the unit cell as part of the energy minimization and seeing the unit cell deform.

(2) If the crystal structure has been determined from powder diffraction data, functional groups having a similar electron-density distribution may have been swapped. For example, N and C—H represent the same number of electrons and may be exchanged without it being noticeable in the Rietveld refinement. Real-space structure solution algorithms may not explore all ring conformations of flexible rings, and a published structure may reflect the input conformation rather than the optimal ring conformation.

(3) Disorder. The crystal structure model resulting from a diffraction experiment records a space and time average, and disorder can be hidden in the anisotropic displacement parameters (ADPs). This is illustrated in Fig. S2 for CSD refcode ANOCOW01 (Zouev *et al.*, 2011 ▸). If the ADPs are ignored, the propyl chain is incorrectly inter­preted as a propenyl chain due to the short distances between the disordered C atoms when their positions are averaged. Only after the large ADPs have been split into pairs of disordered atoms, and the space-group symmetry broken, can the two underlying individual propyl chains be resolved.

(4) Temperature effects other than disorder. If an experimental crystal structure was determined at a relatively high tem­per­a­ture, it may be distorted relative to the ‘0 K’ energy-minimized structure. If the source of distortion is a tem­per­a­ture effect, it should be possible to confirm this by determining the crystal structure at a lower tem­per­a­ture (Bond *et al.*, 2011 ▸).

(5) Inaccuracies in the com­putational methods used. For example, due to Pulay pressure, a decent basis set must be used when energy minimizing crystal structures with the unit cell free to relax. The approximations inherent in PBE+D may affect calculated relative energies of a set of crystal structures, but hardly ever lead to distortions in the energy-minimized structures themselves; however, there are some rare exceptions. In 2014, we observed a space-group change from *C*2/*m* to *R*
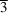
*m* upon energy minimization with PBE+D for one of the polymorphs of butyne [van de Streek & Neumann (2014 ▸); CSD refcode BUTYNE01 (Ibberson & Prager, 1995 ▸)] due to a shift of the layers during the energy minimization that we could not explain. With PBE0+D, the crystal structure of butyne is reproduced without any layer shift and the space group remains *C*2/*m* (Fig. S3). Such failures of PBE+D are rare and we have never observed any failure for large mol­ecules without mol­ecular symmetry, such as pharmaceutical com­pounds.

(6) Unknown reasons. The crystal structure of Form E of crystal structure prediction blind test com­pound XXIII [CSD refcode XAFPAY04 (Samas, 2016); *CSD Communication*, doi:10.5517/cc1kl8dt], distorts when energy minimized with PBE+D (Fig. S4) or PBE0+D, but we have not been able to find a structural model that explains this behaviour.

Incorrect H-atom positions, disorder and minor errors in crystal structures determined from powder diffraction data are not uncommon and affect perhaps one in ten energy minimizations. The remaining three reasons are rare, and less than 1% of energy minimizations are affected by them.

An exception is spurious proton transfer upon energy minimization with a GGA functional such as PBE, which can be regularly observed. When proton transfer is observed, the energy of the crystal structure can no longer be calculated with any degree of accuracy. The positions of the non-H atoms, however, are not affected, and since all measures to com­pare crystal structures described in Section 4 are almost exclusively determined by the positions of the non-H atoms, measures based on crystal structure (as opposed to crystal energy) can still be used.

### Convergence

During geometry optimization, the energy changes between successive minimization steps are monitored, as well as the changes to the atomic coordinates and the unit-cell parameters. When the changes are all smaller than certain pre-set criteria, it is assumed that further minimization steps will have negligible effects on the energy, atomic positions and lattice constants, and the energy minimization is taken to be com­pleted or ‘converged’. Usual convergence criteria are, for example, limits of 0.005 kcal mol^−1^ on the energy dif­ference between successive minimization steps, 0.003 Å on the largest Cartesian displacement and 0.7 kcal mol^−1^/Å on the largest force.

Setting a maximum number of steps is a common safety mechanism to prevent wasting resources on an energy minimization that is undergoing large geometric changes, which indicates that the starting model was probably far from optimal or even wrong (for example, due to missing or extra H atoms). If a maximum number of minimization steps has been set, it is important to check that the minimization com­pleted because the convergence criteria were met and not because the step limit was reached. The chosen step limit may need to be exceeded in cases where the optimization is slow to converge, for example, due to a fairly flat potential energy surface, which may occur for crystal structures with π-stacked mol­ecular layers for example. In contrast, the minimization algorithm is occasionally unable to determine which changes should be made to the crystal structure to decrease the energy and effectively gets ‘stuck’, such that successive changes cause the energy to oscillate; the minimization ultimately aborts when the step limit is reached without having achieved con­vergence. In these cases, the lack of progress can be difficult to distinguish from regular convergence as the minimization may appear to have finished normally. Indeed, literature reports of two successive equivalent DFT energy minimizations resulting in dif­ferent energy minima (*e.g.* Hodge *et al.*, 2020 ▸) might be explained by assuming that the first minimization terminated prematurely, before reaching convergence. If it is suspected that the energy minimization terminated before proper con­vergence, it might be sufficient to start the minimization from scratch taking the semi-optimized crystal structure as the starting point. In cases where the minimization becomes stuck and continuing from the last geometry is insufficient, it may also help to pre-optimize the crystal structure with slightly dif­ferent settings, for example, with the unit cell kept fixed or with a dif­ferent dispersion correction.

### Including pressure

Simulating the effect of pressure is easy and requires negligible com­putational resources: all that is needed is the addition of a pressure–volume (*PV*) term during the energy minimization. This corresponds to an isotropic pressure, often referred to as ‘hydro­static pressure’. It is also possible to apply anisotropic pressures by specifying the applied pressure as a tensor that is entered as a 3×3 matrix; entering any multiple of the identity matrix is equivalent to applying a hydro­static pressure. The effect of standard atmospheric pressure, *ca* 0.0001 GPa, on crystal structures and their relative energies is negligible, but the effect of pressure becomes significant above 0.1 GPa. For example, Fig. S5 shows the effect of a 4.0 GPa applied pressure on the crystal structure of paracetamol (CSD refcode HXACAN12; Boldyreva *et al.*, 2000 ▸), which can be reproduced very well by DFT calculations.

### Hardware and software considerations

The main bottleneck when running quantum-mechanical calculations is memory (RAM), which is why DFT calculations are best run on a High-Performance Computing (HPC) cluster where the calculations can be parallelized over multiple CPUs, increasing the amount of RAM available for the calculation and reducing the wall-clock time. Most energy minimizations with the PBE+D functional, which is a useful com­plementary technique when solving crystal structures from powder diffraction data, can be run on a high-end PC, which at the time of writing is, for example, a 16-core Xeon w5 with 64 GB DDR5 RAM and a Solid State Drive (SSD).

As early as 2016, a validation across dif­ferent DFT packages implementing dif­ferent types of basis sets and dif­ferent numerical methods demonstrated that all provided con­sistent results (Lejaeghere *et al.*, 2016 ▸). Although there may be dif­ferences in the resources required, 0 K energy minimizations with PBE+D and single-point calculations with PBE0+D are now routine, and the final crystal structures and energy dif­ferences should be the same independent of the DFT software used. We do note that the electronic energy does not have a unique zero point, so that only relative energies and not absolute energies should be directly com­pared in most cases. While there are occasional reports in the literature of equivalent DFT energy minimizations resulting in dif­ferent energy minima (*e.g.* Hodge *et al.*, 2020 ▸), this is likely due to an insufficiently tight convergence setting for one of the energy minimizations (*i.e.* one of the minimizations aborting prematurely without having reached the local minimum), which could have been overcome with a little trial and error. For the more than one thousand energy minimizations that we have published thus far, we have used the *CASTEP* (Clark *et al.*, 2005 ▸), *VASP* (Kresse & Furthmüller, 1996 ▸; Kresse & Hafner, 1994 ▸) and *FHI-aims* (Blum *et al.*, 2009 ▸) codes with great satisfaction, but this should not be considered a recommendation against other DFT softwares.

## Best practices for input crystal structures

### Preparing input files, H atoms, disorder and fractional occupancies

The CIF file format can be com­plicated and not all pro­grams may be able to read all CIF files. We have found that the free version of *Mercury* (Macrae *et al.*, 2020 ▸), which is available for Linux, MacOS and Windows, saves very short clean CIF files that can be inter­preted by all pro­grams we have ever used (Table 2 ▸). For mol­ecules at special lattice positions, be careful about the distinction between the asymmetric unit and the expanded mol­ecule; the latter is the default in *Mercury*. The free and open-source *critic2* code (Otero-de-la-Roza *et al.*, 2014*b* ▸) is useful for converting CIF files to the appropriate input file formats used by most popular QM codes.

By far the most common problems with experimental crystal structures relate to H atoms, which may be missing or not form the correct hy­dro­gen-bonding pattern. The automatic addition of missing H atoms in *Mercury* should add H atoms bonded to C atoms correctly, but –OH H atoms are added in a random orientation and may have to be repositioned manually. When an incorrect number of H atoms is present, the QM calculation still assumes an overall neutral charge, which can result in dangling bonds. In such cases, the only sign that something is wrong with the structural model might be convergence problems due to a vanishing band gap that results from performing a spin-restricted calculation on a system with unpaired electrons.

Disorder remains one of the hardest features of mol­ecular crystals to treat com­putationally. While QM pro­grams do not allow fractionally occupied atoms to match the fractional occupancies in disordered mol­ecular crystal structures, disordered atoms can be correlated. Thus, even if fractional occupations were possible, a model in which electrons inter­act with the average electron density of the disordered atoms would lead to incorrect crystal energies. It is, however, straightforward to write separate CIF files for the major and minor com­ponents of a disordered crystal structure using *Mercury*. In more com­plicated cases, preparing crystal structure models suitable for energy calculations may require breaking the space-group symmetry, *e.g.* if an infinite chain of hy­dro­gen bonds is propagated across twofold axes or inversions; see CSD refcode ARUFIE (Tejchman *et al.*, 2015 ▸) for an example.

For non-stoichiometric hydrates, it is often possible to construct structural models with fully occupied atoms that approximate the experimental stoichiometry and that lead to acceptable atomic coordinates and unit-cell parameters. However, we are not aware of a generally applicable method to calculate their energies and the configurational entropy contribution to their free energies, and such calculations are beyond the scope of the present article.

Overall, the effect of disorder on the atomic coordinates, unit-cell parameters, electronic energies and configurational entropy contribution to the free energies must be carefully investigated on a case-by-case basis. After many years of research into this topic, we wrote a com­prehensive guide to the calculation of the structures, energies and free energies of disordered mol­ecular crystal structures, which is to be submitted in the near future (van de Streek *et al.*, 2026 ▸).

### Normalize the *X*—H bond lengths

The electron density of H atoms is usually displaced towards the non-H atom to which it is bonded. Thus, the positions of the electron-density maxima and the H-atom nuclei do not coincide and are typically about 0.1 Å apart. As crystallographers tend to position the H atoms at the electron-density maxima, the *X*—H bond lengths are about 0.1 Å too short in most experimental single-crystal structures. This leads to strong forces, large atomic displacements and large energy changes in the initial stages of the energy minimization, which serve no other purpose than to position the H atoms correctly. Convergence of the energy minimization can, therefore, be accelerated by normalizing the *X*—H bond distances in the input structure to match the values found from neutron diffraction experiments, which measure the nuclear positions.

### Choice of unit cell

The choice of unit cell is extremely important in QM calculations on periodic systems as it will affect the com­pu­tational cost and smoothness of convergence in the energy minimization. In particular, the more orthogonal the unit-cell axes are, the less they correlate and the more numerically stable the calculations can be expected to be. Thus, while crystallographers tend to prefer standard space-group settings over orthogonality of unit-cell axes, transformation to a non-standard setting may im­prove convergence by reducing linear dependencies. Let us consider the crystal with CSD refcode CUKGAR01 (Feast *et al.*, 2009 ▸) as an example. When refined in the standard setting of *P*2_1_/*c*, it has a unit-cell angle β = 132.04°, while transformation to the non-standard setting of *P*2_1_/*n* results in β = 92.00° and reduces the correlation between the *x*- and *z*-coordinates. Selecting an appropriate set of *k*-points is also easier when the unit cell is as orthogonal as possible, and very acute or obtuse angles may lead to more *k*-points than necessary, increasing the com­putational re­sources. It is therefore recommended to make all unit-cell angles as close as possible to 90° before energy minimizing a crystal structure.

A related consideration is the greater the unit-cell volume, the greater the required com­putational resources. This means that centred unit cells can be reduced to primitive unit cells to speed up the DFT calculations, which can be especially useful for R- and F-centred unit cells. However, standard transformation matrices may lead to very acute or very obtuse unit-cell angles, so some trial and error may be needed in practice.

For example, consider the crystal structure of the 1,2,4-tri­bromo­benzene isomer (CSD refcode UYUQAJ; Bujak *et al.*, 2021 ▸), which has a space group of *Fdd*2 with a unit-cell volume of 9313 Å^3^. Conversion to a primitive unit cell using the transformation matrix [0 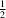

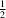
, 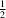 0 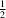, 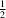

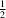
 0] leads to a volume of 2328 Å^3^ and a very acute angle of β = 20.6°. A subsequent transformation using the matrix [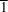

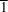
 1, 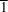 0 1, 0 1 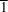] reduces the orthogonality defect and gives unit-cell angles of 90.38, 92.86 and 82.36°, all close to 90°. However, after the second transformation, the matrix representation of one of the symmetry operators is [1 1 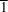, 0 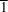 0, 0 0 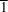], which is not one of the 64 standard representations and may, therefore, lead to error messages in crystallographic pro­grams. If non-standard matrix representations cause problems, the space group can be reduced to *P*1 or *P*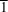 (if the original space group contained an inversion at the origin). Conversion to a lower-symmetry space group will not usually affect the energy minimization, except for some crystal structures that undergo reversible phase transitions as a function of tem­per­a­ture. In almost all such cases, the high-tem­per­a­ture (HT) phase has the higher space-group symmetry, such that a ‘0 K’ energy minimization of the HT phase in *P*1 or *P*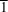 may convert to the low-tem­per­a­ture phase. Note that the HT phase should be less stable at 0 K and its higher minimized energy, caused by the fewer degrees of freedom in the imposed space-group symmetry, is not an artefact.

If multiple experimental crystal structures are available, the structure determined at the lowest tem­per­a­ture should be the easiest to energy minimize with the unit cell free because the calculations reflect the unit-cell volume at 0 K. All other experimental determinations of the same polymorphs should trivially converge to the same energy-minimized structure, but probably at the cost of requiring more minimization steps due to the greater adjustment of the unit-cell volume that is needed.

## Comparing crystal structures

Before using the energy-minimized crystal structure for energy calculations, it is important to confirm that the crystal structure has not distorted too much as a result of the energy minimization. If the structure distorted considerably, something is likely wrong and it should not be carried forward to further energy calculations. Many methods are available to com­pare the initial and the energy-minimized crystal structure (Mayo & Johnson, 2025 ▸), but the two simplest options are (i) visually through an overlay or (ii) by calculating the Root-Mean-Square Cartesian Displacement (RMSCD).

**Figure 1:**
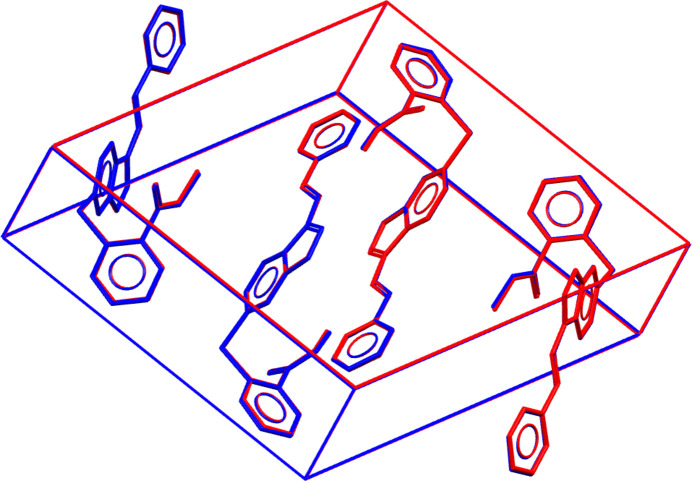
Overlay of the experimental crystal structure of Form XLI of axitinib at room tem­per­a­ture (red) and the same structure after energy minimization with PBE+D (blue). The *FHI-aims* result with PBE+MBD-nl/lightdense is shown, yielding an RMSCD = 0.059 Å.

*Mercury* can be used to create visual structure overlays. Fig. 1 ▸ shows the overlay of the experimental crystal structure of form XLI of axitinib and the corresponding energy-minimized PBE+D structure. We selected red (warm) for the experimental crystal structure, which includes thermal effects, and blue (cold) for the calculated ‘0 K’ structure. A black background is environmentally unfriendly in case the document is printed, so a white or transparent background is recommended. The high quality of reproduction between the experimental and energy-minimized crystal structures shown in Fig. 1 ▸ is routinely achievable with PBE+D.

The qu­antity used for com­parison of experimental and energy-minimized crystal structures is the RMSCD, excluding H atoms. ‘Cartesian displacement’ is not uniquely defined when the unit cells of the two crystal structures to be com­pared are dif­ferent, as is the case when the lattice parameters are allowed to vary in the energy minimization. In this work, the Cartesian displacement for an atom in two crystal structures (1) and (2) is defined as (see Neumann & Perrin, 2005 ▸).
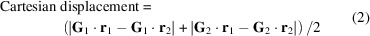
where **r**_*i*_ are the fractional coordinates of the atom in crystal structure *i* and **G**_*i*_ is the transformation matrix from fractional to Cartesian coordinates for crystal structure *i*. This definition of Cartesian displacement has the advantages that it is symmetric with respect to the two structures to be com­pared, that it varies smoothly upon smooth distortions of either or both of the structures, and that there is no need for a user-defined parameter such as the number of mol­ecules used for the com­parison. Note that the COMPACK algorithm (Chis­holm & Motherwell, 2005 ▸), as implemented in *Mercury*, com­pares atomic coordinates without taking into account the dif­ferences in the unit-cell parameters and thus yields dif­ferent RMSCD values.

In 2017, we determined how well 200 crystal structures were reproduced upon four repeated energy minimizations from slightly dif­ferent starting points (Hempler *et al.*, 2017 ▸). We established that the average discrepancy between two energy minimizations was an RMSCD of 0.0045 Å, although this was not explicitly reported in the article.

Based on a set 225 single-crystal X-ray structures, we previously established that correct structures distort with an average RMSCD of only 0.084 Å when energy minimized with the unit cell free, while RMSCD values greater than 0.25 Å indicate an error in the experimental crystal structure (van de Streek & Neumann, 2010 ▸). Crystal structures determined from powder diffraction data are less accurate and, for a set of 215 such crystal structures, we found that correct structures may have RMSCD values of up to 0.35 Å (van de Streek & Neumann, 2014 ▸).

When assessing whether an input crystal structure distorted significantly upon geometry optimization, it is important to note that there is no linear relationship between the distortion, the energy and the correctness of a crystal structure. Whereas a correct crystal structure can generally be easily identified based on a single energy minimization, a large distortion does not imply a major issue. Indeed, very minor issues in a crystal structure, such as an incorrect orientation of an –OH group, can lead to large distortions and large energy dif­ferences. Conversely, a small distortion does not necessarily imply that the crystal structure model is close to correct as there may be multiple offsetting contributions. This makes hunting for mistakes in crystal structures a challenge.

An analogous problem to com­paring *in silico* and experimental crystal structures is deciding whether a pair of crystal structures are the same form or dif­ferent polymorphs. There is an extensive literature on this subject (Sacchi *et al.*, 2020 ▸; Mayo *et al.*, 2022 ▸; Mayo & Johnson, 2025 ▸) and, in many cases, no DFT calculations are required to make a conclusive determination. However, energy minimizations may be helpful to resolve the question of same *versus* dif­ferent in edge cases where COMPACK or powder-dif­ference com­parisons yield ambiguous or contradictory results. If one aims to determine if two similar crystal structures are actually the same form, it is greatly advantageous to first generate slightly distorted unit cells using the average of the two sets of lattice parameters prior to fixed-cell energy minimization, as this allows for the DFT energy minimizations to converge to identical, as opposed to merely similar, structures. Establishing that two crystal structures are identical is easier than qu­anti­fying similarity.

Comparing an experimental crystal structure before and after energy minimization is trivial because the unit-cell setting, space-group setting and definition of the asymmetric unit, and even the order of the individual atoms, are all guaranteed to be identical. The two crystal structures can, therefore, be com­pared without any prior alignment. The same is not true when com­paring two arbitrary crystal structures of the same com­pound, or even of the same polymorph. In cases of missed symmetry, symmetry-breaking phase transitions and triclinic unit-cell angles very close to 90°, there is no guarantee that reducing the two (or more) unit cells to their standard settings leads to the same origin and orientation for all crystal structures, and finding the corresponding transformation relating the two (or more) structures can be time consuming.

Simulated powder diffraction patterns have the advantage that they are one-dimensional and have a fixed origin; in other words, simulated powder diffraction patterns can always be com­pared directly without the need for a prior alignment step. Furthermore, simulated X-ray powder diffraction patterns are, by definition, relatively immune to common crystallographic errors from X-ray diffraction experiments. These include misplaced H atoms, missed symmetry and incorrect element assignments such as C instead of N. However, tem­per­a­ture or pressure dif­ferences and energy minimization all cause minor anisotropic dif­ferences in the unit-cell parameters, leading to peak shifts in the simulated powder diffraction patterns. Point-by-point measures for powder diffraction profiles, such as *R*_wp_, are very sensitive to minor dif­ferences in peak positions and, thus, should not be used to qu­antify the similarity of two simulated powder diffraction patterns. Several similarity measures for powder diffraction patterns account for peak shifts (Otero-de-la-Roza, 2024 ▸; Mayo *et al.*, 2022 ▸; Habermehl *et al.*, 2014 ▸; van de Streek & Motherwell, 2005 ▸), which can be described as special cases of the normalized weighted cross-correlation of the two powder diffraction patterns (de Gelder *et al.*, 2001 ▸). For the overlay shown in Fig. 1 ▸, the normalized weighted cross-correlation value of the simulated powder diffraction patterns with triangle value *l* = 3.0° 2θ is 0.986.

To assess the effect of tem­per­a­ture on similarity measures, we consider an extreme example. Bond (2021 ▸) published a survey of thermal unit-cell expansion coefficients in the CSD and found that the thermal expansion of refcode family BIJWAS is exceptionally large. BIJWAS01 (Rogers & Green, 1986 ▸) was determined at room tem­per­a­ture, which is the most common tem­per­a­ture for PXRD measurements, whereas BIJWAS02 (Rogers & Richards, 1987 ▸) was determined at 123 K, which is a common tem­per­a­ture for single-crystal measurements. After energy minimization, the structures are indistinguishable. The overlays of the experimental structures and their energy-minimized counterparts are shown in Fig. S6 (see supporting information). With COMPACK, which com­pares the Cartesian atomic coordinates while ignoring the unit-cell parameters, the RMSCD values are 0.35 and 0.10 Å for the RT and LT structures, respectively. The RMSCD, as defined by Neumann & Perrin (2005 ▸), yields 0.18 and 0.075 Å for the RT and LT structures, respectively. The normalized weighted cross-correlation values of the simulated powder diffraction patterns with triangle value *l* = 3.0° 2θ are 0.935 and 0.997, respectively, *cf*. the values for axitinib Form XLI, de­ter­mined at room tem­per­a­ture.

## Guiding principles

The preceding sections dealt with the com­parably straightforward tasks of energy optimizing and com­paring crystal structures (atomic coordinates and unit-cell parameters); cal­culating and com­paring crystal energies generally requires more thought and is described in the next two sections.

### Structures converge faster than energies with increasing method quality

It is very well established in the field of com­putational chemistry that mol­ecular geometries are much less sensitive to the chosen level of theory than are electronic energies. Similarly, crystal structures (unit-cell parameters and atomic coordinates) converge faster and are much easier to reproduce than energies. As a consequence, decisions that can be made based on the reproduction of experimental unit-cell parameters and non-H atomic positions by alternative crystal structure models (*e.g.* with dif­fering H-atom positions) may be more reliable than the com­parison of small energy dif­ferences.

**Figure 2:**
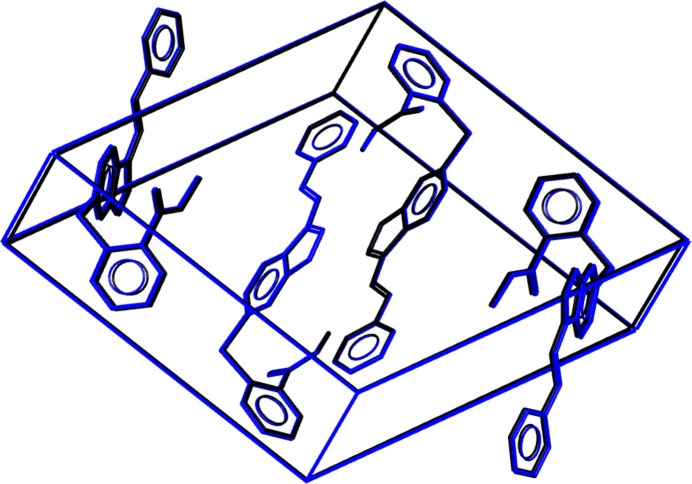
Overlay of the crystal structure of Form XLI of axitinib energy minimized with PBE+D (blue) and energy minimized with PBE0+D (black); RMSCD = 0.021 Å. Whereas the energies may be significantly dif­ferent, the atomic coordinates and unit-cell parameters from GGA functionals such as PBE and hybrid functionals such as PBE0 are essentially the same. LDA functionals (not shown) are not suitable and lead to distortions.

In the following, we will illustrate how to make use of the more rapid convergence of crystal geometries to reduce com­putational cost. Various combinations of density functionals and basis sets will be written using the method/basis notation; examples of small and large basis sets would be ‘lightdense(r)’ and ‘tight’, respectively. With this notation, crystal energies calculated with PBE+D/small and PBE0+D/small, or with PBE+D/small and PBE+D/large, may be quite dif­ferent. However, energy-minimized crystal structures ob­tained with PBE+D/small and PBE0+D/large are hardly dis­tin­guishable. For the particular example of axitinib Forms I, IV, VI, XXV and XLI, the RMSCD values between the crystal structures energy minimized with PBE+D/lightdense and PBE0+D/lightdense are 0.034, 0.032, 0.033, 0.027 and 0.021 Å, respectively (see Fig. 2 ▸).

**Figure 3:**
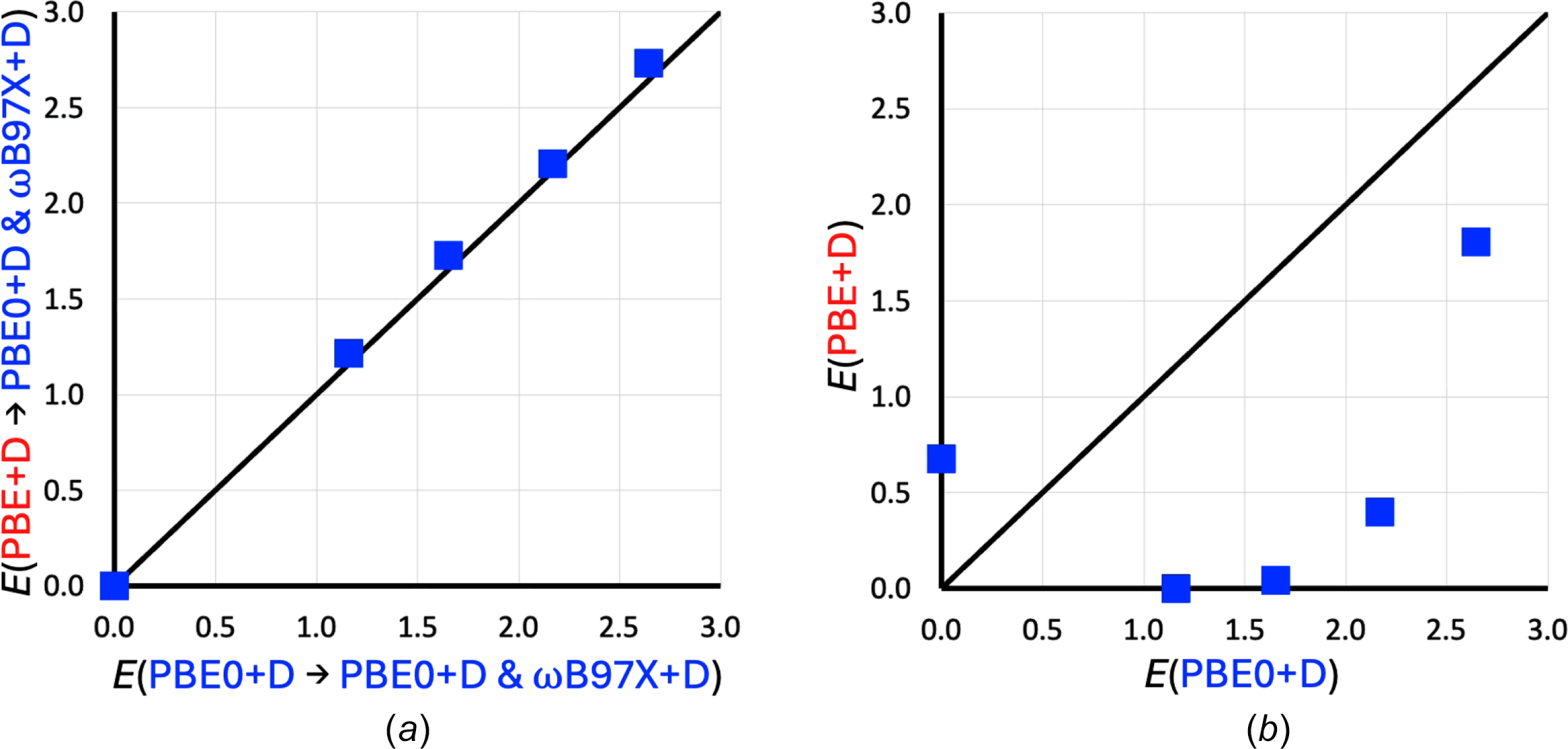
(*a*) The expensive PBE0+D/tight & ωB97X+D single-point energies of five axitinib polymorphs after energy minimization with the cheap PBE+D/lightdense method (*y* axis) and the expensive PBE0+D method (*x* axis). The line *y* = *x* is drawn to guide the eye. A cheap energy minimization followed by an expensive single-point energy calculation gives the same results as an expensive energy minimization followed by an expensive single-point energy calculation. (*b*) The relative energies of the same five axitinib polymorphs ob­tained with the cheap PBE+D method (*y* axis) and expensive PBE0+D method (*x* axis). Whereas the PBE+D and PBE0+D structures are almost indistinguishable, the PBE+D and PBE0+D energies are very dif­ferent. The line *y* = *x* is drawn to guide the eye.

The energy potential used for the energy minimization (DFT functional, dispersion correction, basis set and *k*-points) can be com­pletely independent of the energy potential applied for the calculation of the single-point energies. Thus, it is recommended to energy minimize crystal structures with PBE+D/small, and to subsequently add a cheap single-point energy calculation with the superior PBE0+D/large method, rather than performing very expensive PBE0+D/large geom­etry optimizations, as illustrated in Fig. 3 ▸(*a*). In com­parison, Fig. 3 ▸(*b*) shows the very dif­ferent behaviour of PBE+D and PBE0+D energies.

While the memory and CPU demands of a single-point calculation with PBE0 with a large basis set can still be prohibitive, such a calculation can be further simplified by splitting it into multiple single-point calculations by considering each of them a correction that is added to a cheaper reference energy. For example, the PBE0+D/large energy can be reliably approximated as
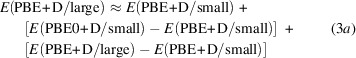
where *E*(PBE+D/small) is the reference energy ob­tained from a single-point calculation with the energy method PBE+D/small. The first correction is the dif­ference between PBE0+D and PBE+D, while the second is the dif­ference between the large and small basis set. Each of the correction terms im­proves one aspect of the reference method at a time, as graphically shown in Fig. S7. The above result can also be written as
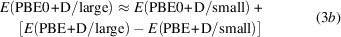
but Equation (3a[Disp-formula fd3]) shows the symmetry between the individual corrections, allows the individual corrections to be qu­anti­fied separately, and makes it more obvious how to extend the equation to include additional correction terms.

Within the 0 K approximation, PBE0+D energies are cur­rently the state of the art for inter­molecular inter­actions. Conformational energies, on the other hand, can still be im­proved by additional single-point calculations on the individual com­ponent mol­ecules that form the crystal, called a monomer correction or a single-mol­ecule correction, with either dispersion-corrected second-order Møller–Plesset per­tur­bation theory (MP2D) or range-separated hybrid functionals. ωB97X+D (Chattopadhyay *et al.*, 2025 ▸) is one such range-separated hybrid that provides a good balance of accuracy and com­putational cost; for easier typesetting, ωB97X is sometimes written as wB97X. Such isolated-mol­ecule calculations typically use Gaussian basis sets, which should be of at least triple-ζ quality and ideally include diffuse functions to ensure accurate conformational energies. For the example of MP2D, the calculation is as follows:
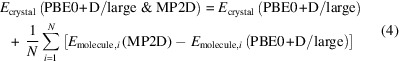
where the sum runs over all mol­ecules within the unit cell. Note that, if the single-point energy of the crystal is approximated as a linear combination of multiple single-point energies [as in Equation (3)], the single-point energies of the isolated mol­ecules should be evaluated in the same fashion before they are subtracted from the MP2D mol­ecular energy.

### Exploit error cancellation in the com­putation of relative energies

Energy does not have a natural zero point and it is therefore not possible to refer to ‘the’ energy of a crystal structure, only to energy dif­ferences. The use of relative, as opposed to absolute, energies allows one to make significant use of error cancellation when performing QM calculations. In the context of this article, error cancellation exclusively refers to the cancellation of errors as a result of considering energy dif­ferences only (and not the other type of error cancellation arising when two shortcomings in a com­putational method have opposing effects). As an example, even though the PBE functional is poor in describing the energies involved in breaking and forming bonds, energy dif­ferences between polymorphs can be calculated with much greater accuracy than mol­ecular formation energies. This is the case because the errors in the mol­ecular formation energies are the same in both polymorphs and offset when considering energy dif­ferences.

Error cancellation becomes an even more important factor when com­paring two alternative models for a crystal structure, for example, to check the correct orientation of an acetyl group in a crystal structure determined from medium-quality powder diffraction data. The calculated energy dif­ference between two such com­peting models can be expected to be much more reliable than the calculated energy dif­ference between two arbitrary experimental polymorphs due to their high degree of structural similarity. As a general rule, the more similar the crystal structures, the greater the error cancellation. For example, com­puted energy dif­ferences between polymorphs with the same hy­dro­gen-bonding pattern will be more accurate than energy dif­ferences between polymorphs with dif­ferent hy­dro­gen-bonding schemes.

### Compare the signal to the error bar

An area that is all too often ignored is the question of the error bars, as several dif­ferent error sources affect calculated energies. By far the smallest, and negligible com­pared to all other error sources, is the numerical error; it is the com­putational equivalent of what is called *precision* in experiment, *i.e.* it refers to the reproducibility when an energy minimization on the same crystal structure is repeated multiple times from slightly dif­ferent starting points. The standard deviation of the atomic coordinates due to numerical error is *ca* 0.0045 Å (see above) and, for energy dif­ferences, these errors should contribute well below 0.1 kcal mol^−1^, and usually less than 0.05 kcal mol^−1^.

There are relatively few reliable benchmarks available to assess the accuracy of DFT methods for absolute and relative lattice energies of mol­ecular crystals, with the most notable being the X23 (Otero-de-la-Roza & Johnson, 2012*a* ▸; Reilly & Tkatchenko, 2013 ▸; Dolgonos *et al.*, 2019 ▸) and ICE13 (Brandenburg *et al.*, 2015 ▸; Della Pia *et al.*, 2024 ▸) data sets. In 2023, we published what to the best of our knowledge is the first com­parison of calculated tem­per­a­ture-dependent free-energy dif­ferences *versus* experimental free-energy dif­ferences (Firaha *et al.*, 2023 ▸). These free energies included a single-point correction with PBE0+D for the crystal energy, an MP2D correction for the mol­ecular energy, and the contribution of the phonons calculated using the supercell method. For mol­ecular crystal structures of chemical com­pounds con­taining up to 50 atoms (including H atoms), the 0 K PBE+D energy dif­ferences between energy-minimized crystal structures have an error bar of about 0.75 kcal mol^−1^; this error bar is the *accuracy* of the calculated energy dif­ferences com­pared to experiment. A 0 K single-point calculation with PBE0+D on the energy-minimized crystal structures reduces the error bar to about 0.50 kcal mol^−1^, but a PBE0 single-point calculation takes about one order of magnitude more resources (com­puter memory and CPU time) than a PBE single-point calculation.

Adding the contribution from the phonons reduces the error bar to about 0.25 kcal mol^−1^. This means that the error in the calculated energy dif­ference between two mol­ecular crystal structures due to the neglect of tem­per­a­ture is ap­proximately 0.25 kcal mol^−1^. This is con­sistent with the results of Nyman & Day (2015 ▸), who found that for 70% of pairs of experimental polymorphs, the phonon contribution to the free energies (|Δ*F*_vib_|) is smaller than 0.25 kcal mol^−1^. However, phonon calculations require about two orders of magnitude more resources than a PBE single-point calculation and are beyond the scope of this article. For more sophisticated free energy calculations on mol­ecular crystal structures, the articles by Hunnisett *et al.* (2024 ▸), Firaha *et al.* (2023 ▸) and Wood *et al.* (2026 ▸), and references therein, are good starting points.

### If an energy dif­ference is unexpected, the higher-energy structure is likely in error

Occasionally, a reasonable expectation value for the sign of an energy dif­ference is available, for example, from com­petitive slurry experiments. If the com­putationally predicted stability order is substanti­ally opposite to that expected, it is almost always the energy that is higher than expected that is the problem. The reasoning is simple: if a small change to a structural model were to lower the energy of the structure, nature would already have selected the structure with the lower energy. Any change to a correct model is therefore expected to increase the energy; this is merely another application of the Anna Karenina principle. Incorrect models have energies that are systematically higher than expected, and correcting the model lowers the energy, aligning the energy with the expectation.

An excellent example is the case of morphine HCl trihydrate (CSD refcode MORPHC; Gylbert, 1973 ▸), the structure of which was investigated com­putationally by Braun *et al.* (2014 ▸). The authors found that the crystal structure distorted upon energy minimization, indicative of a likely error, and the calculated lattice energy was higher than expected. The structure was solved in 1973 and contains one O—H⋯H—O inter­action and one O⋯Cl^−^ inter­action, with the proton positions assigned to chemically unlikely positions by the crystallographer. Reshuffling the protons results in one O—H⋯O hy­dro­gen bond and one O—H⋯Cl^−^ hy­dro­gen bond, lowering the energy of the trihydrate by 14.1 kcal mol^−1^ (at the PBE+D level) and resolving the problem that the trihydrate was calculated to be highly unstable with respect to the anhydrate (see Fig. S8 in the supporting information).

### Experimental unit-cell parameters are reliable independent of the atomic coordinates

In Rietveld refinement, also called whole-profile fitting, all data points are used in the refinement of the unit-cell parameters and the peak positions are generally only affected by one error, namely, the 2θ zero-point error, which can trivially be modelled. The atomic coordinates, on the other hand, are determined by the intensities, which suffer from much more severe errors, namely, peak overlap and preferred orientation, which are much harder to model. As a result, the experimental unit-cell parameters from X-ray and neutron single-crystal data after about 1980, and from X-ray and neutron powder diffraction data, are highly accurate, even if the coordinates of one or more of the atoms is not. Note that the same is not necessarily true for electron diffraction, where the unit cell is determined with lower precision than X-ray diffraction (Gemmi *et al.*, 2026 ▸).

If a centre of symmetry has been overlooked in the determination of a single-crystal structure, this leads to singularities in the refinement and the atomic positions are unreliable (Schomaker & Marsh, 1979 ▸), but the unit-cell parameters are not affected. For example, the crystal structure of CSD refcode PASQAB (Konarev *et al.*, 1997 ▸) was published in *Cm*, *Z*′ = 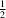, and the C—C bond lengths in the fullerene mol­ecule range between the unphysically short and long distances of 1.04 and 1.84 Å (see Fig. S9). However, the correct space group is *C*2/*m*, *Z*′ = 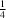. After averaging the symmetry-equivalent atoms in the corrected space group, the spread is still 1.23 to 1.62 Å, and Marsh *et al.* (2002 ▸) writes ‘further refinement is clearly needed’. DFT energy minimization is not affected by the missing centre of symmetry, and in our energy-minimized DFT structure in the incorrect space group *Cm*, *Z*′ = 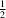, the bond lengths are between the much more reasonable values of 1.40 and 1.46 Å (see Fig. S9). In the absence of further experimental data, the best structural model, with reasonable bond lengths for all mol­ecules and including all H atoms, is the result of a DFT energy minimization in *C*2/*m*, *Z*′ = 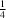 with the experimental unit cell fixed.

### The more additional degrees of freedom, the lower the energy

It should not be necessary to mention this explicitly in a scientific publication, but we have seen cases where, in the com­parison of two models, the one with additional degrees of freedom was selected as correct because it had a numerically better figure of merit, such as a more favourable energy. Per the variational principle, additional degrees of freedom will lower the energy, or at least result in it staying the same. However, it is possible that the structure with a higher DFT energy may be a better match to an experimental crystal structure due to neglect of thermal effects or errors inherent in the density functional.

## Special cases

### Comparing energies of dif­ferent chemical com­pounds

Only energies of crystal structures with the same chemical com­position can be com­pared. As we restrict ourselves to the solid state, this means that only polymorphs (and cocrystals of the same stoichiometry) can be com­pared in practice. To com­pare the energies of cocrystals of dif­ferent stoichiometries, the chemical com­positions must be balanced; see, for example, Cruz-Cabeza *et al.* (2008 ▸) and Taylor & Day (2018 ▸).

To com­pare the energies of crystal structures corresponding to dif­ferent hydration states, the com­position must be balanced either with ice at 0 K (which is a poor approximation for water vapour) or with isolated water mol­ecules (for which there is no hy­dro­gen bonding, prohibiting the error cancellation required for more accurate relative energies). Further, note that the relative stability of dif­ferent hydration states is properly a function of the relative humidity, so any energy com­parison of dif­ferent hydration states in which the energy of pure water is a constant cannot be expected to capture reality. For a proper com­putational com­parison of the free energies of dif­ferent hydration states, see Firaha *et al.* (2023 ▸).

### Racemic *versus* enanti­opure phases

Thermodynamically, the *R* enanti­omer, the *S* enanti­omer and the racemate of a chiral com­pound are three dif­ferent chemical com­pounds, but no chemical bonds are broken or formed and the energies can therefore be com­pared as if they were polymorphs. The crystal structures of the *R* enanti­omer and the *S* enanti­omer are expected to be each other’s mirror image, so their energies should be identical and calculations on only one enanti­omer need be performed. However, the race­mate will, in most cases, have a dif­ferent crystal structure and must be considered separately.

The racemate can crystallize in space groups with an im­proper symmetry element (a symmetry element that turns a left hand into a right hand), whereas an enanti­omerically pure com­pound cannot. A racemate therefore has more options available when crystallizing than an enanti­opure com­pound. If the racemate has not crystallized in one of the Sohncke space groups (or, to be pedantic, has not crystallized in a Sohncke space group with *Z*′ = 2 with the two mol­ecules in the asymmetric unit having opposite chirality), *i.e.* if the racemate has not crystallized as a so-called conglomerate, one would therefore expect that it has exploited this additional degree of freedom and that its crystal energy is lower (at the same tem­per­a­ture and pressure). Cases where both racemic and conglomerate phases of the same crystal can be ob­tained experimentally provide excellent benchmarking data to assess the accuracy of com­putational methods such as DFT, as the relative free energies of the two forms can be determined with high precision from experimental measurements of the enanti­omeric excess at the eutectic point (Otero-de-la-Roza *et al.*, 2014*a* ▸; Buchholz *et al.*, 2017 ▸).

### Deuteration

^2^H (deuterium) is twice as heavy as ^1^H (protium), which changes the vibrations and thereby the atomic equilibrium positions and the crystal free energy; because of zero-point vibrations, this is the case even at 0 K. In 0 K DFT calculations, in which vibrations do not play a role, the atomic positions, electron densities and electronic energies are exactly identical by definition. Experimentally, the dif­ferences in positions of ^2^H and ^1^H atoms are very small, even for perdeuterated com­pounds. This results in electron densities, powder X-ray diffraction patterns, unit-cell parameters and atomic coordinates for deuterated and non-deuterated com­pounds that are to all intents and purposes identical. However, some exceptions are known (Merz & Kupka, 2015 ▸) and, in those cases, 0 K DFT calculations cannot be used to reproduce or predict the energy dif­ferences.

### Salts *versus* cocrystals

The PBE functional incorrectly favours salts over cocrystals (LeBlanc *et al.*, 2018 ▸). In our experience, there is often virtually no energy barrier between the salt and the cocrystal forms, *i.e.* the proton’s energy potential is a single well, not a double well. This means that energy minimization will typically yield the same final structure (salt or cocrystal), regardless of the starting structure (salt or cocrystal). It is therefore not possible to calculate im­proved energies for both models to decide which is more stable, and the energy calculations as described in this article cannot be used to address the question if a crystal structure is a salt or a cocrystal. Moreover, in resonance-assisted hy­dro­gen bonds (Dračínský *et al.*, 2015 ▸; Štoček *et al.*, 2022 ▸), the H atoms are delocalized and can only be properly described by a dynamic model and not a static structure from energy minimization. However, as the non-H atoms are not affected by such a dynamic proton exchange, the distortion of a crystal structure model upon energy minimization as qu­anti­fied by the RMSCD of the non-H atoms can still be used as a measure for the correctness of a crystal structure.

The distinction between a salt and a cocrystal is very important for the pharmaceutical industry, yet the low X-ray scattering power of a H atom can com­plicate the experimental distinction *via*X-ray diffraction. The com­putational determination of the proton position is therefore an active area of research (Abramov & Wang, 2024 ▸; Bal & Collas, 2024 ▸; Beran, 2025 ▸) but beyond the scope of this best practice guide.

### Isomers and diastereomers

PBE+D can give significant errors for the relative energies of chemical isomers, even in cases such as dif­ferences between linear and branched alkanes, in which the numbers and types of all atoms and bonds are conserved, such that the inter­conversion reaction between the isomers would be isodesmic (Goerigk *et al.*, 2017 ▸). However, if two (or more) mol­ecules are sufficiently similar, error cancellation can facilitate com­parison of their lattice energies. For example, to show that the dif­ferences in their melting points were caused by the contributions of their mol­ecular symmetries to their entropies, the lattice energies of three tri­bromo­benzene isomers were calculated and com­pared (van de Streek, 2022 ▸), relying on their chemical similarity to maximize error cancellation.

Additionally, diastereomers, unlike enanti­omers, are chem­i­­cally distinct com­pounds so their energies cannot be directly com­pared. However, diastereomers are a subset of structural isomers where the bonding is maximally conserved and only the arrangements about two or more stereocentres dif­fer, so error cancellation will reduce PBE+D errors in the relative single-mol­ecule energies.

### Tautomers

In the crystal structures of tautomers, the positions of the H atoms in the individual crystal structures are generally un­am­big­u­ous and are reproduced very well by PBE+D energy minimizations (Fig. S10). Comparison of the energies of crystal structures con­sisting of the same tautomer should be no dif­ferent from any other crystal energy calculation. Prob­lems arise when the crystal energies of structures con­sisting of dif­ferent tautomers are com­pared: the inherent energy errors of the PBE+D and PBE0+D functionals when bonds are broken and formed are often greater than the energy dif­ferences between polymorphs. As a bare minimum, the mean­ingful com­parison of the energies of polymorphs con­taining dif­ferent tautomers requires not only a PBE0 single-point energy correction, but also an MP2D or ωB97X+D monomer correction. Fig. S11 shows the PBE+D/small crystal energies, PBE0+D/large crystal energies, ωB97X+D mol­ecular energies and PBE0+D/large & ωB97X+D crystal energies for thio­barbituric acid Form II (PABNAJ, enol tautomer), Form IV (PABNIR, 1:1 keto–enol tautomers) and Form III (THBARB01, keto tautomer) (Chierotti *et al.*, 2010 ▸), illustrating the poor performance of the PBE and PBE0 functionals for the energy dif­ferences between tautomers. More sophisticated quantum-mechanical methods may be needed for a more general approach to the modelling of the energies of tautomers (Perry *et al.*, 2025 ▸).

Determining the correct tautomer for a crystal structure model is still possible if the appropriate metrics are considered, as illustrated for the CSD entry NILVEL (Nfor *et al.*, 2013 ▸). Here, inspection of the hy­dro­gen bonds with *Mercury* reveals two non-bonded N⋯N contacts of 2.907 Å; in the absence of a H atom, this implies a short contact between two lone pairs, which is highly unlikely. Upon energy minimization with PBE+D, the RMSCD is 0.84 Å, whereas the RMSCD for a model corresponding to an alternative tautomer is only 0.11 Å (Figs. S12 and S13). As the low RMSCD alone is sufficient to be confident in structural assignment, more sophis­ticated energy calculations are not needed, although the PBE+D energy dif­ference between the two models (19.1 kcal mol^−1^ in favour of the alternative tautomer) is also con­sistent with the geometry argument.

## Examples

### Example I: confirming the paroxetine HCl 0.9-hydrate structure

The crystal structure of paroxetine HCl hemihydrate was a late-appearing phase, displacing the thermodynamically less stable paroxetine HCl 0.9-hydrate, and the two forms were the subject of a patent battle (Bučar *et al.*, 2015 ▸). Only one crystal structure for paroxetine HCl 0.9-hydrate, determined from synchrotron powder diffraction data, is present in the CSD (CSD refcode EHOXEE; Howard *et al.*, 2003 ▸). As this crystal structure was submitted as a *CSD Communication*, there was no associated peer-reviewed publication and no associated publicly available experimental powder diffraction data or plot of the Rietveld refinement. The experimental powder diffraction data was not deposited with the Inter­national Center for Diffraction Data (IDD) either. Thus, we turned to DFT modelling to confirm or dispute the deposited crystal structure.

While the H atoms of the water mol­ecule are not present in the CSD structure, their approximate positions are obvious and were added using an in-house C++ code. In the calculations, the occupancy of the water mol­ecule is exactly 1.0 rather than 0.9, and only additional experimental data can determine the exact occupancy. We then energy minimized the experimental structure, including the unit-cell parameters. The resulting RMSCD in the non-H-atom positions is 0.20 Å (see Fig. S14), from which we conclude that the experimental structure is most likely correct. As unit-cell parameters from PXRD are generally accurate, we can prepare a reliable model of the experimental structure, including H atoms, by keeping the unit-cell parameters at their experimental values and energy minimizing the atomic coordinates.

### Example II: pyrazine-ring rotation in glipizide

The crystal structure of glipizide was determined from laboratory powder X-ray diffraction data (Burley, 2005 ▸). Two orientations are possible for the pyrazine ring, dif­fering only in the exchange of a CH group and an N atom, both representing seven electrons. Burley commented on the two possibilities in the article and chose one at random. To confirm or refute this choice, both models were energy minimized with PBE+D with the unit-cell free. The model as published distorts with an RMSCD of 0.72 Å [Fig. S15(*a*)], while the alternative model hardly distorts with an RMSCD of 0.13 Å [Fig. S15(*b*)] and has a crystal energy that is 4.6 kcal mol^−1^ more favourable. This leads us to conclude that the alternative model is actually correct. A Rietveld refinement with polymorph-dependent restraints against the original experimental data is shown in Fig. S16.

### Example III: determining H-atom positions in riboflavin

Riboflavin contains four hydroxyl groups and there are mul­tiple possible arrangements for the hy­dro­gen-bond net­work. The crystal structure was determined from syn­chro­tron powder X-ray diffraction data and the authors com­pared the energies of several hy­dro­gen-bonding patterns from DFT calculations with the unit cell fixed (CSD refcode XARWUM; Guerain *et al.*, 2021 ▸). The published hy­dro­gen-bonding pattern looks plausible and satisfies all hy­dro­gen-bond donors. Moreover, when the unit cell is kept fixed, the RMSCD of the published structure upon energy minimization is only 0.23 Å [Fig. S17(*a*)]. However, energy minimization with the unit cell free provides additional degrees of freedom, allowing for a large distortion of the unit cell, with an RMSCD of 0.54 Å [Fig. S17(*b*)]. One of the hydroxyl groups can be rotated by 180°, turning a OH⋯O=C hy­dro­gen bond and a chain of OH⋯OH⋯O=C hy­dro­gen bonds into one chain of con­certed OH⋯OH⋯OH⋯O=C hy­dro­gen bonds. The RMSCD after energy minimization with the unit cell free is then only 0.13 Å [Fig. S17(*c*)] and is 0.86 kcal mol^−1^ more stable than the distorted structure. A Rietveld refinement of the corrected hy­dro­gen-bonded structure with polymorph-dependent restraints against the original experimental data is shown in Fig. S18. After energy minimization with the unit-cell parameters fixed at their published values, the energy dif­ference between the published and the corrected crystal structure models is 4.3 kcal mol^−1^.

## Supplementary Material

Additional figures. DOI: 10.1107/S2053229626006169/ky3233sup1.pdf

## Figures and Tables

**Table 1:** Relative PBE+D single-point energies for a set of crystal structure models for axitinib Form XLI (CSD refcode VUSDIX04) ob­tained from dif­ferent energy-minimization protocols

Energy-minimization protocol	Single-point energy (kcal mol^−1^)
As published, no minimization	308.4
*X*—H normalized, no minimization	10.54
Energy minimized, unit cell fixed	0.30
Energy minimized, unit cell free	0.00
<|Δ*E*|>^1^	0.85

Note: (1) average of the ten PBE+D energy dif­ferences between the five known axitinib polymorphs (I, IV, VI, XXV and XLI) after full energy minimization.

**Table 2:** Licensed and unlicensed features in *Mercury*

Feature	Free *Mercury*	Licensed *Mercury*	JvdS′ github repository^1^
Adding H atoms	**X**	**X**	
Overlay structures		**X**	
Visualize hy­dro­gen bonds	**X**	**X**	
Normalize **X**—H bonds		**X**	**X**
Calculate RMSCDs		**X**	**X**
Calculate void space		**X**	**X**
Reduce centred cells	**X**	**X**	**X**
Convert to *P*1	**X**	**X**	**X**
Simulate PXRD	**X**	**X**	**X**
De Gelder’s PXRD similarity measure		**X** ^2^	**X**
Split disorder ADPs	(**X**)^3^	(**X**)^3^	**X**
Reduce orthogonality defect	**X** ^4^	**X** ^4^	**X**

Notes: (1) https://github.com/JvdS147/ComputationalCrystallographersAnonymous, open-source, C++, can be com­piled on Windows, Linux and MacOS. (2) In the ‘Searches’ panel under ‘Options’ | ‘Customize Columns’ add ‘PXRD similarity’. (3) A feature request has been sent to the Cambridge Crystallographic Data Centre. (4) Implicit in the ‘Transform to Reduced Cell’ command.
