# Supplementary material for: Best practices in ‘0 K’ DFT energy calculations on mol­ecular crystal structures
Source: Acta Crystallogr C Struct Chem. 2026 Jul 8;82(Pt 8):383–400. doi: 10.1107/S2053229626006169 (PMC13439669; doi:10.1107/S2053229626006169)
Supplement: Supplementary file 1 [file c-82-00383-sup1.pdf]

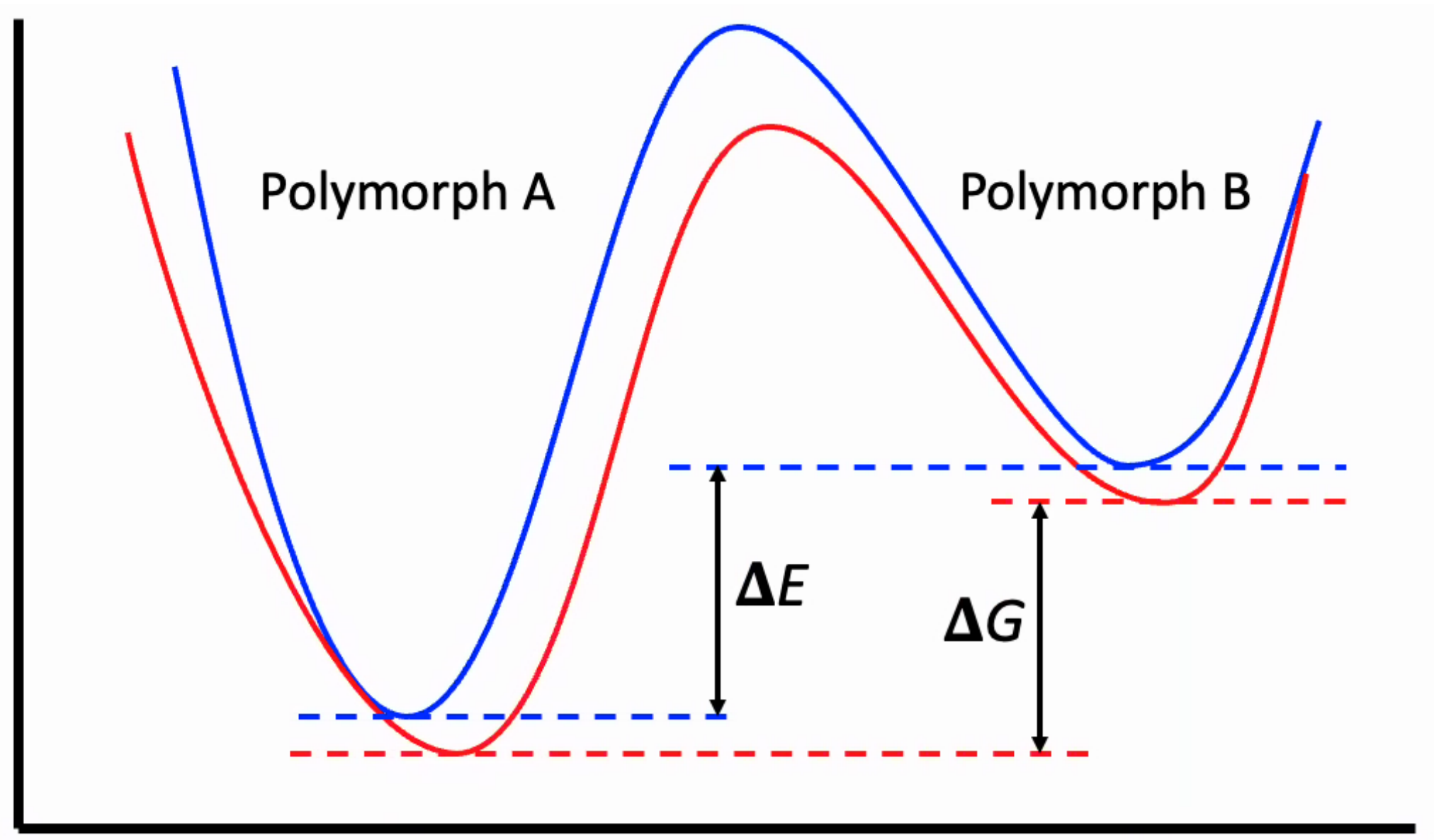

Figure S 1a. Calculated 0 K crystal energies in blue and experimental free energies at 300 K in red (*y*-axis), as a function of an abstract 1-dimensional coordinate representing the crystal structure (*x*-axis).

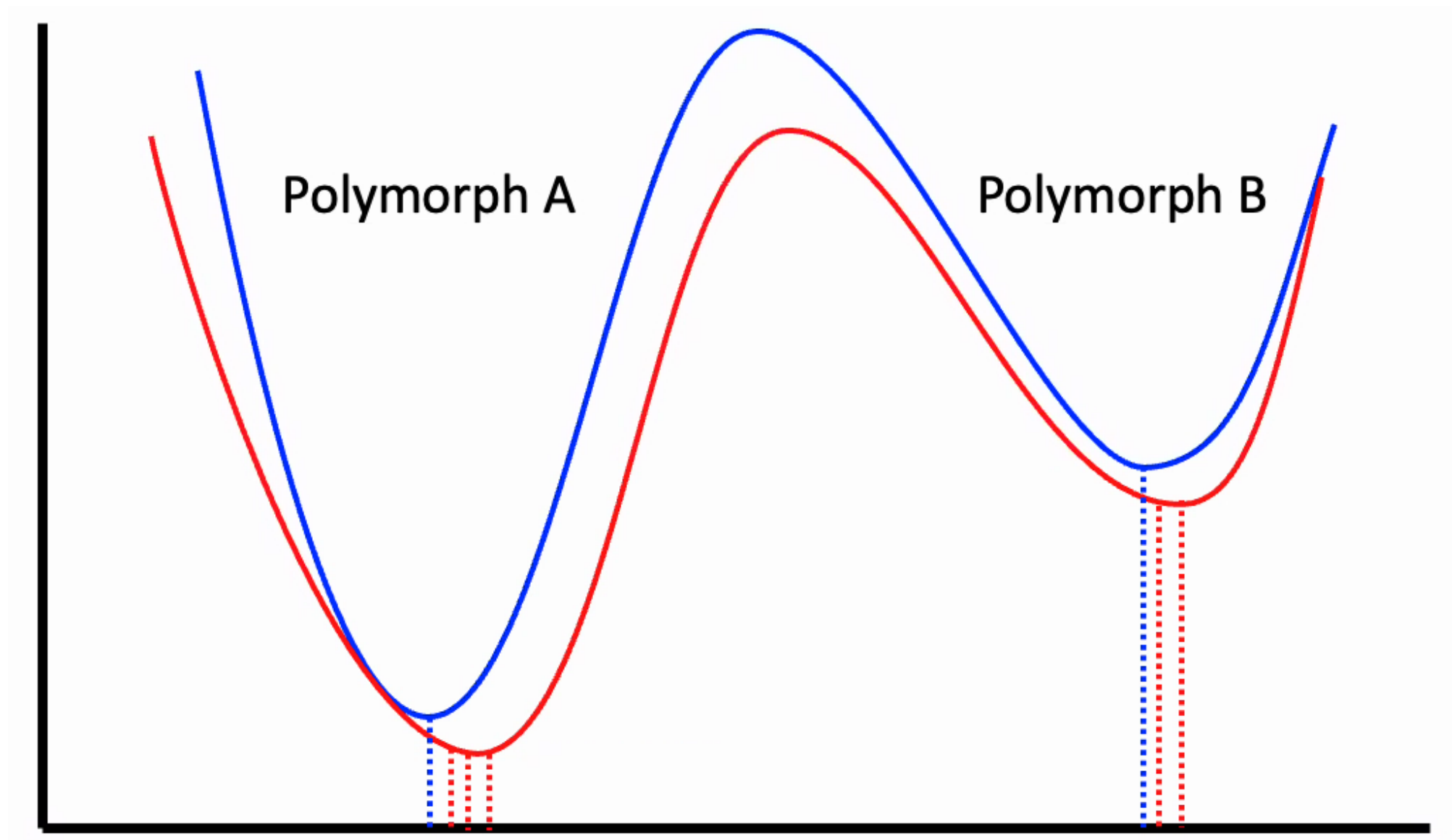

Figure S 1b. Calculated 0 K crystal energies in blue and experimental free energies at 300 K in red (*y*-axis), as a function of an abstract 1-dimensional coordinate representing the crystal structure (*x*-axis). Multiple experimental determinations correspond to slightly different structures and therefore to slightly different calculated single-point energies. After energy minimisation, all experimental determinations of the same polymorph converge to the same structure.

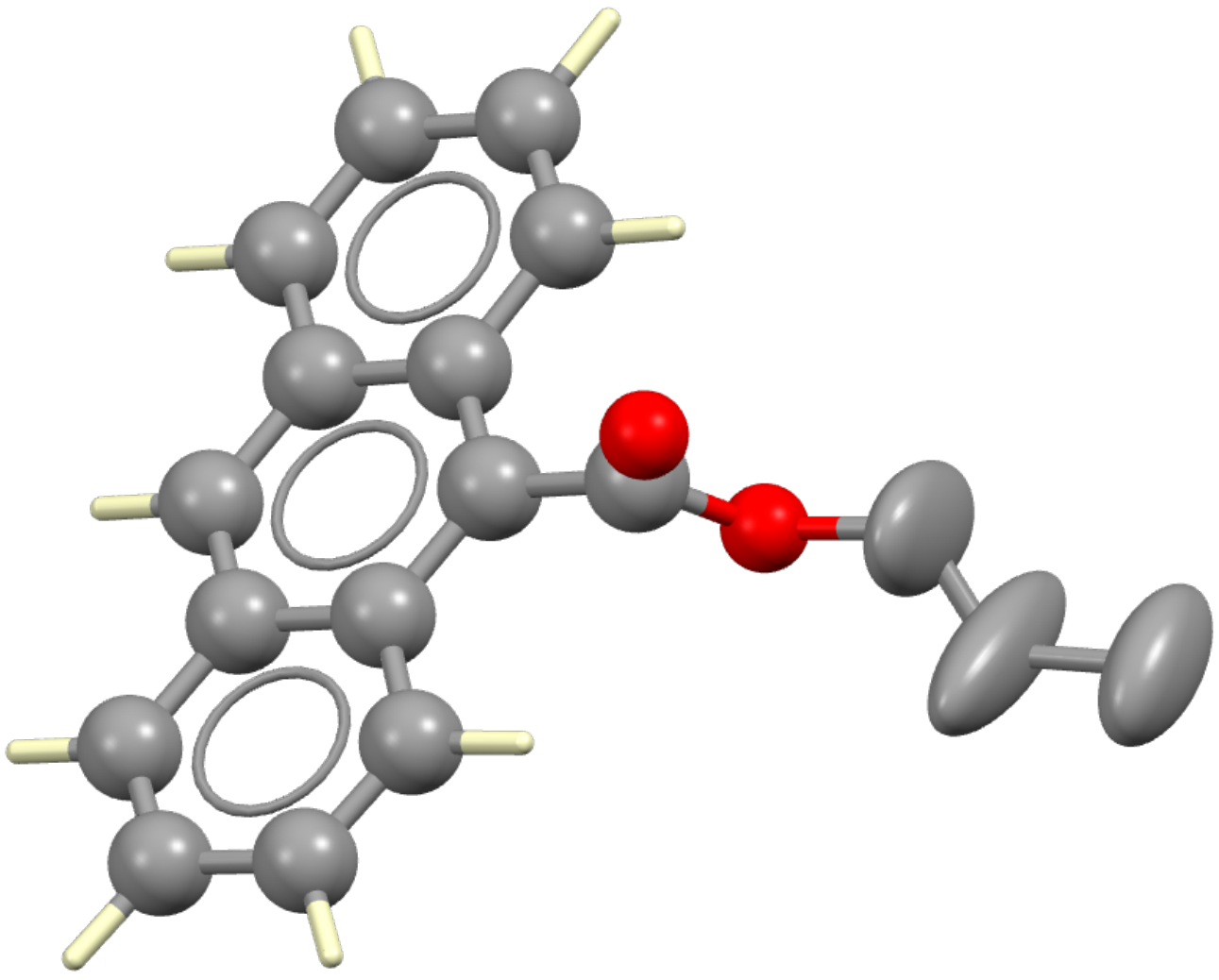

Figure S 2a. CSD reference code ANOCOW01, experimental structure as published, disorder has been modelled through large ADPs.

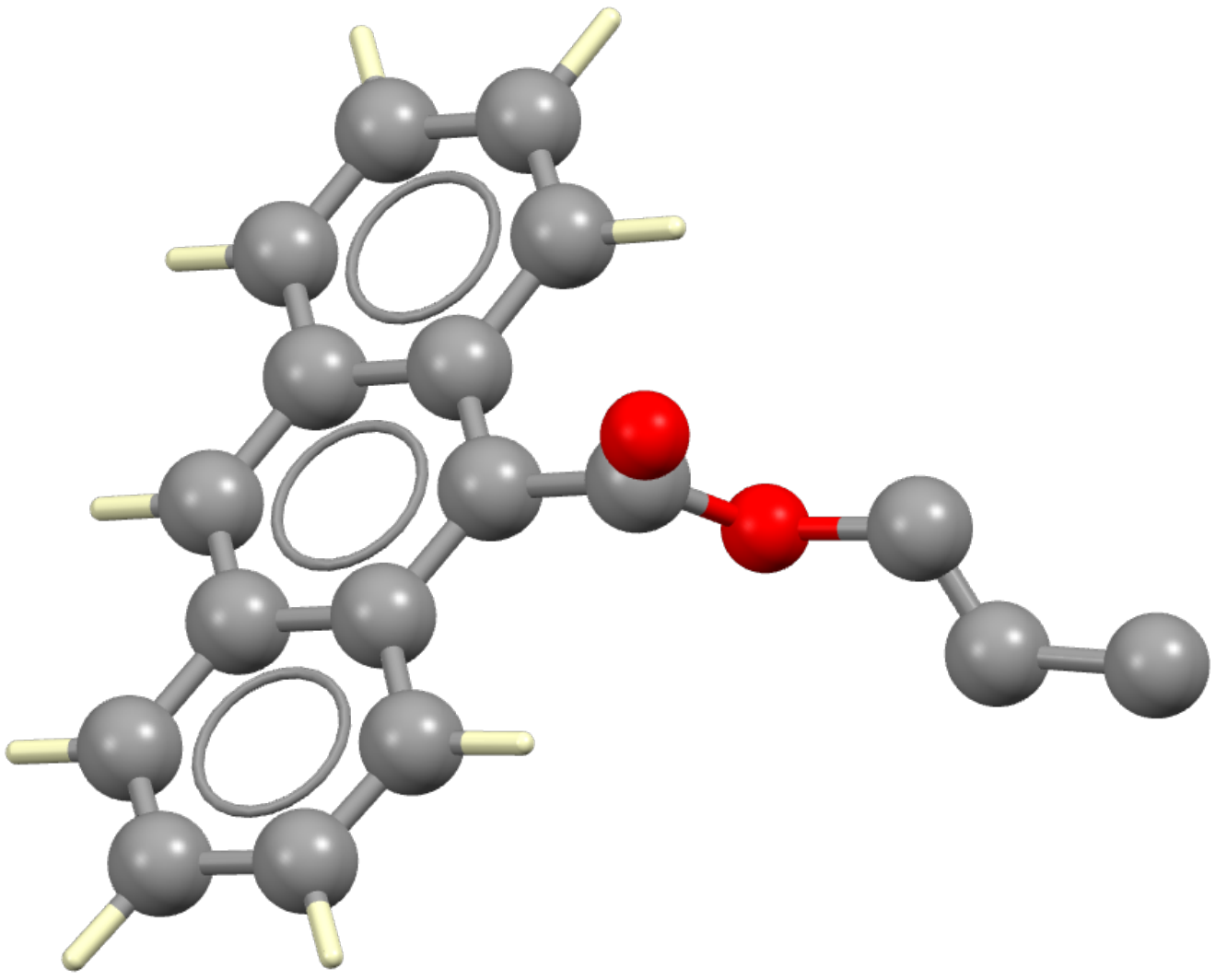

Figure S 2b. When the ADPs are ignored, the disordered atoms are incorrectly represented by their average positions.

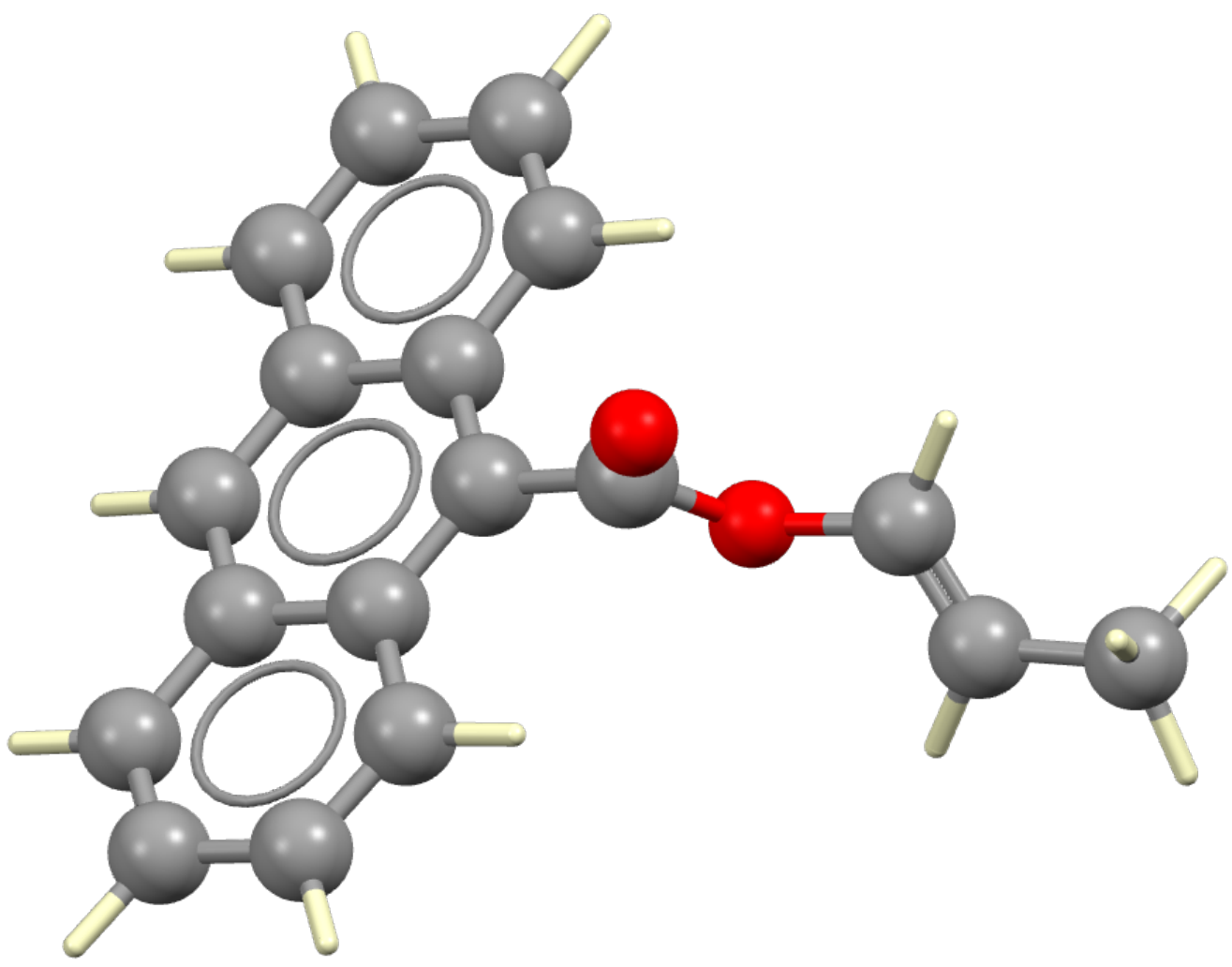

Figure S 2c. Incorrect bond-type assignment and hydrogen-atom placement based on averaged atomic positions. Based on the C–C distance, a single bond has been interpreted as a double bond.

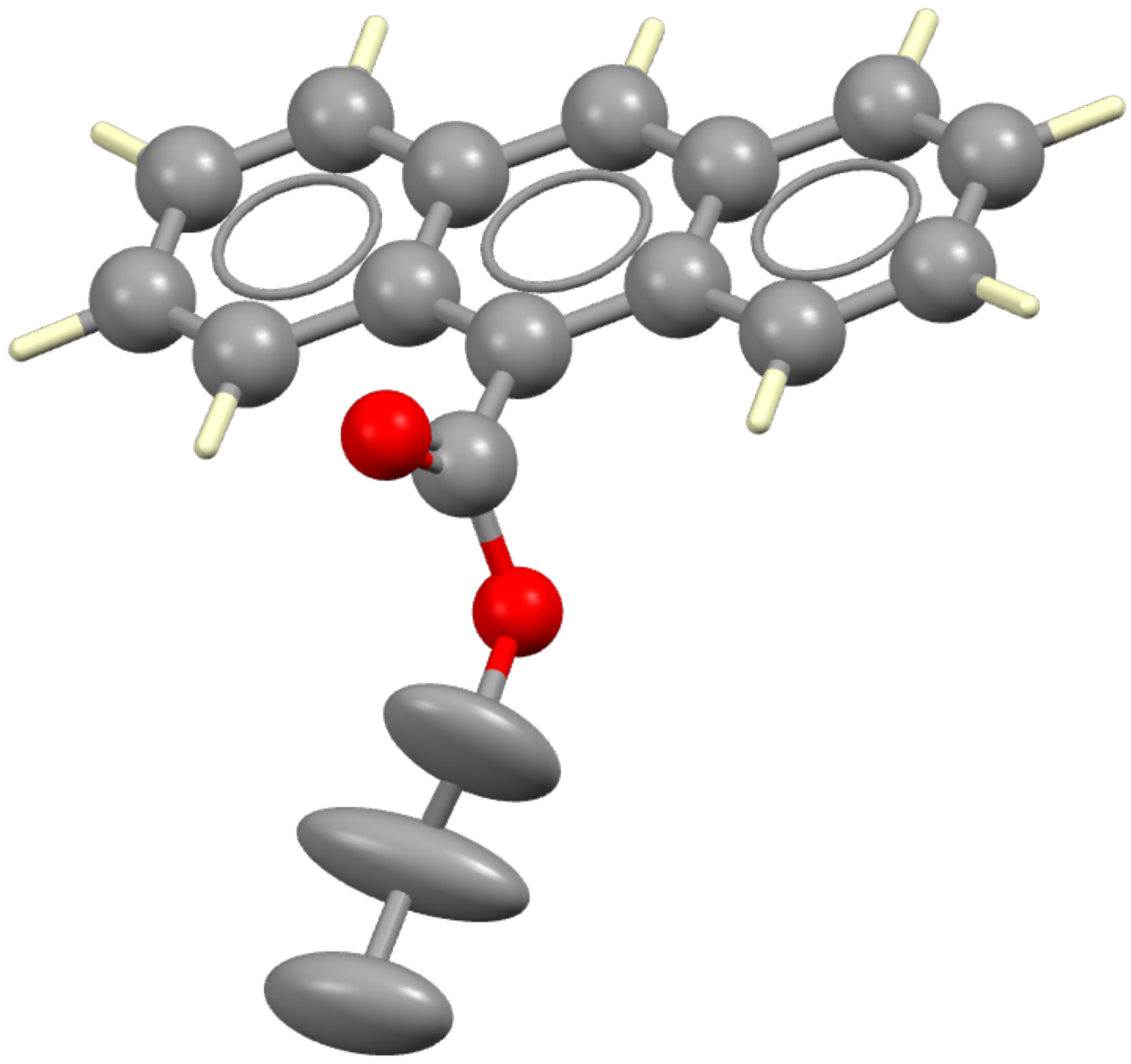

Figure S 2d. CSD reference code ANOCOW01, experimental structure as published, disorder has been modelled through large ADPs.

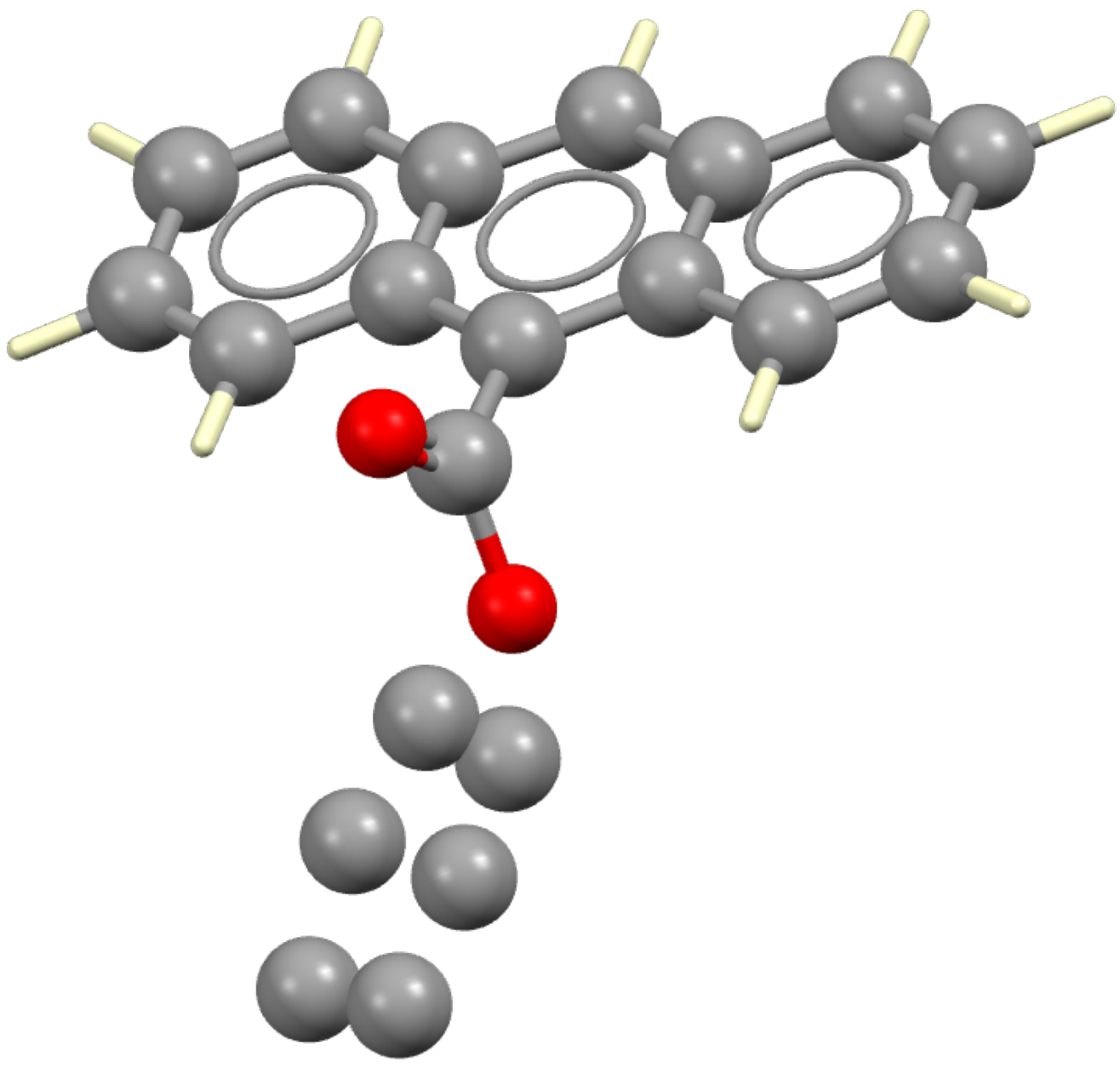

Figure S 2e. Splitting each large ADPs ellipsoid along its principal axis with the largest eigenvalue into two separate atoms.

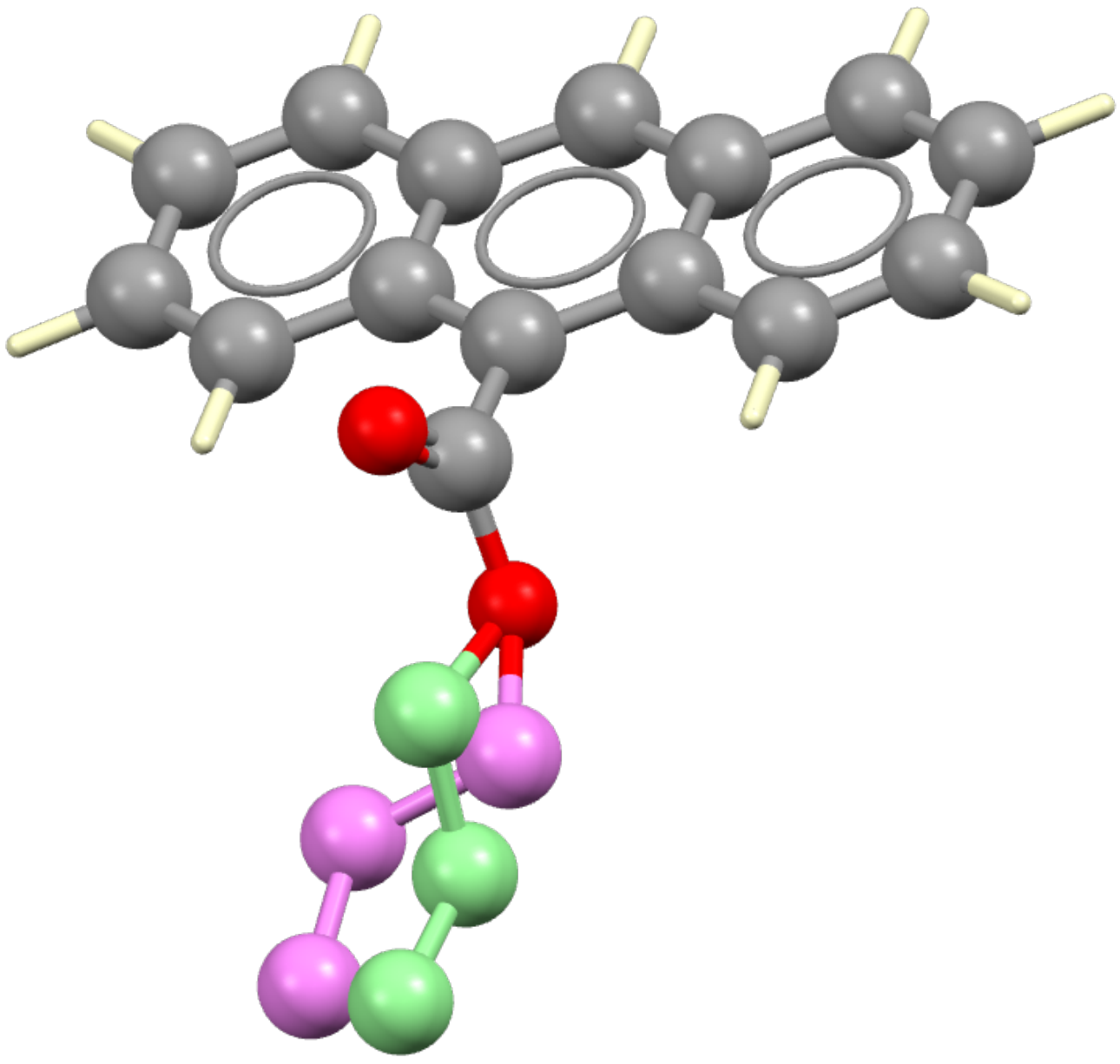

Figure S 2f. Correct interpretation of the disorder as two separate propyl chains.

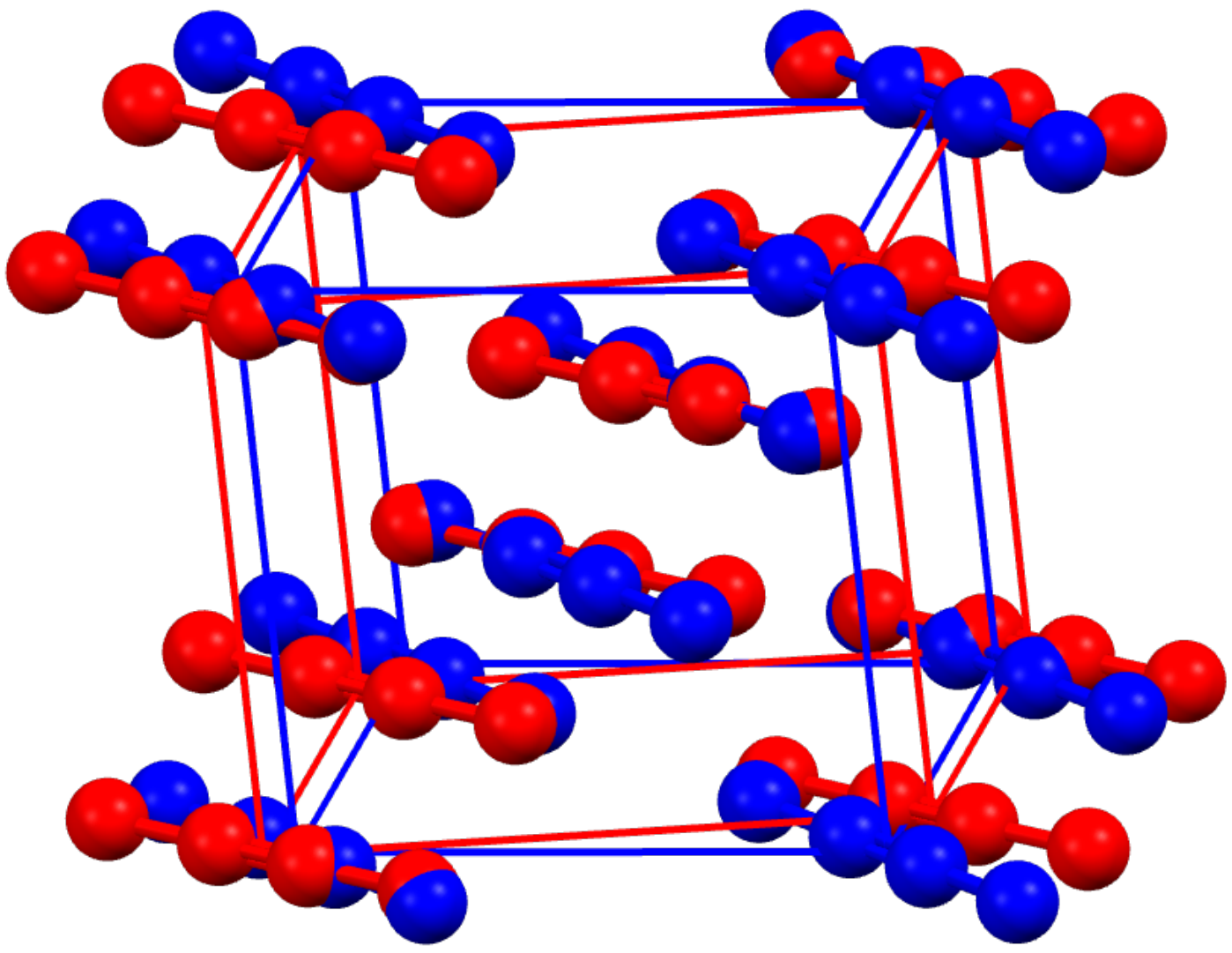

Figure S 3a. Overlay of the experimental crystal structure of BUTYNE01 (red) and the same structure after energy minimisation with PBE+D (blue). The structure distorts considerably. RMSCD = 0.048 Å.

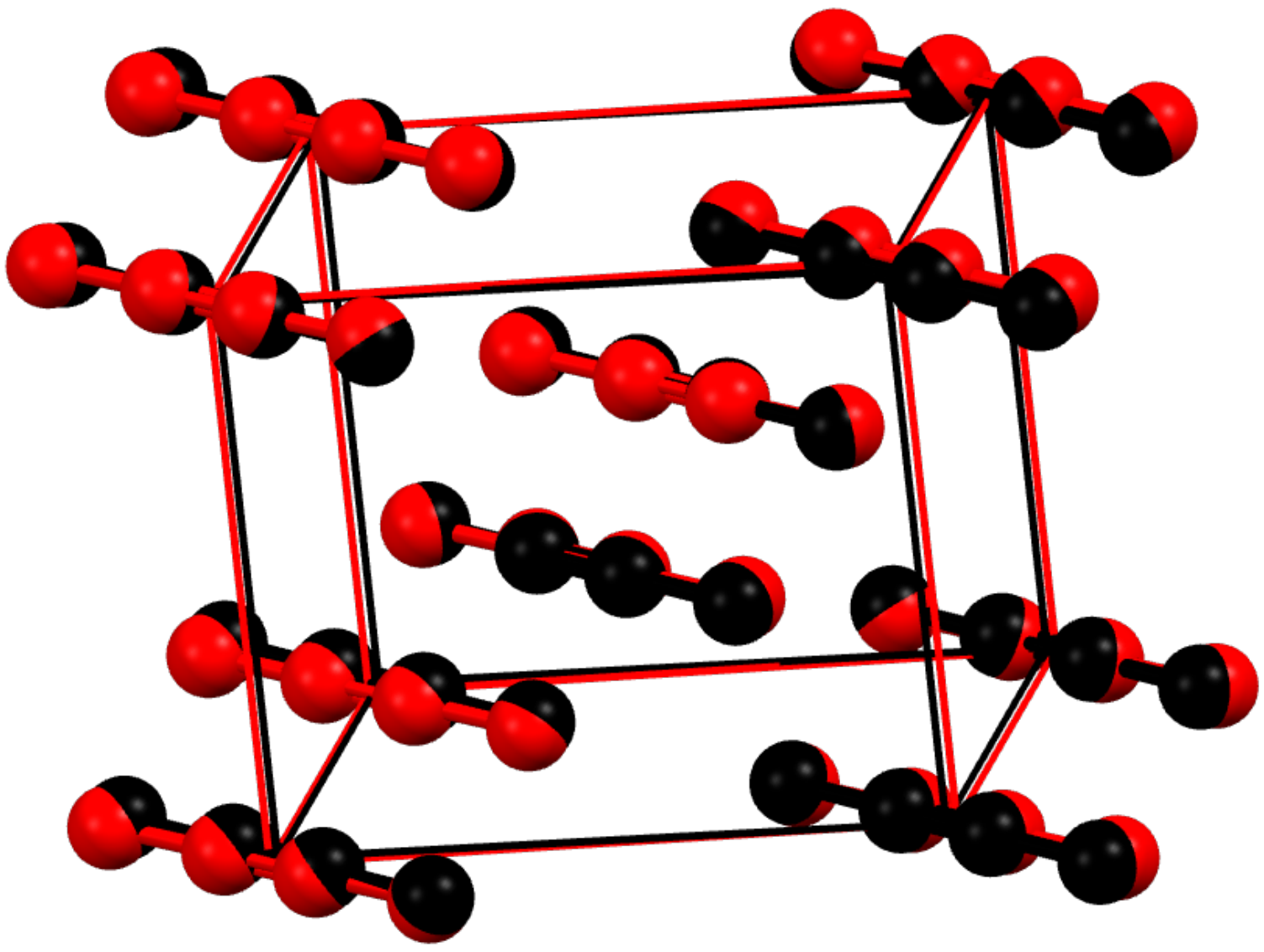

Figure S 3b. Overlay of the experimental crystal structure of BUTYNE01 (red) and the same structure after energy minimisation with PBE0+D (black); RMSCD = 0.0055 Å. PBE0 is a better approximation for the exchange-correlation functional than PBE, and the crystal structure is reproduced much better. This demonstrates that the experimental crystal structure is correct and that the PBE functional is unsuitable for this compound.

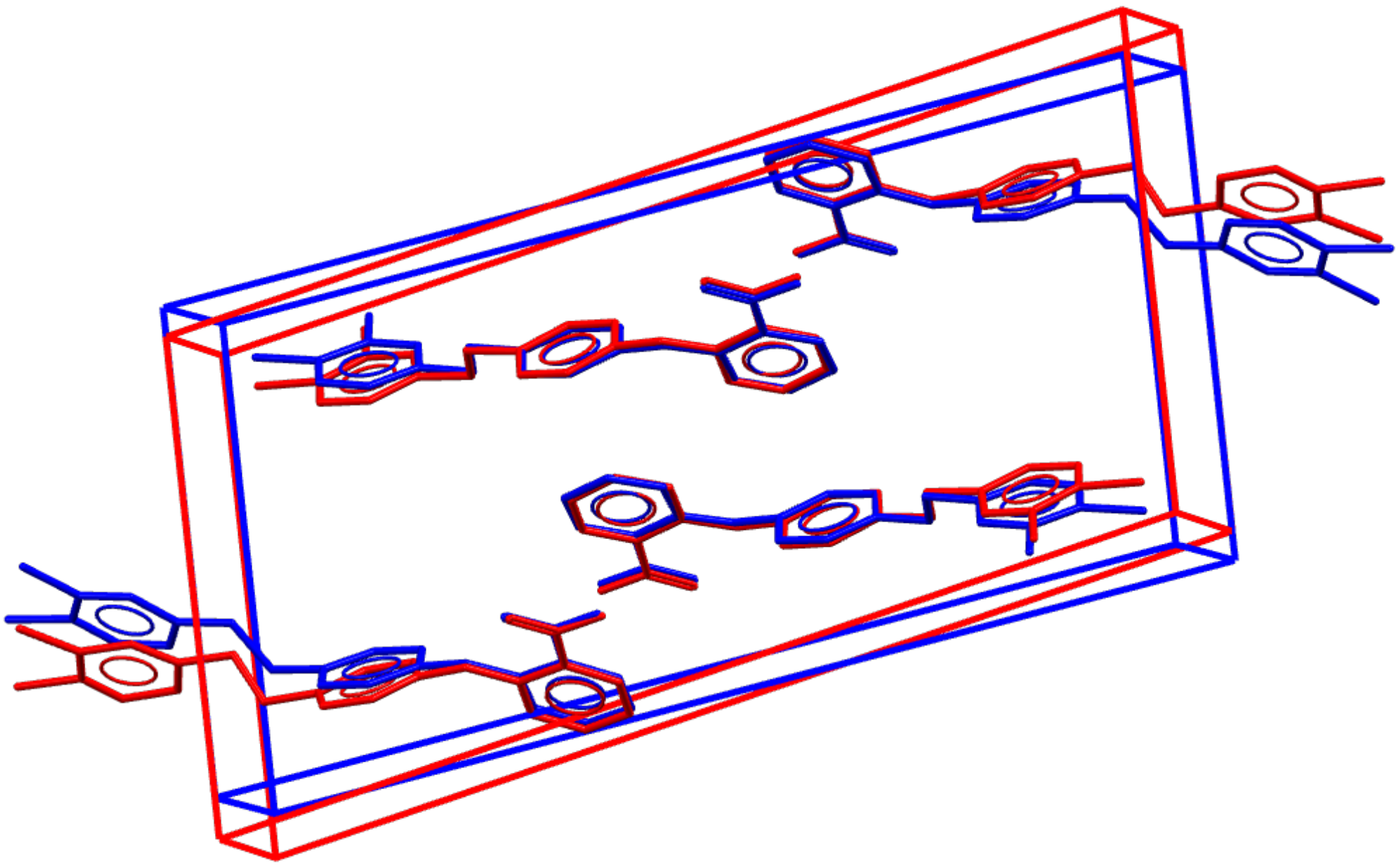

Figure S 4. Overlay of the experimental crystal structure of crystal structure prediction blind test compound XXIII Form E (red) and the same structure after energy minimisation (blue). The structure distorts for unknown reasons. RMSCD = 0.31 Å.

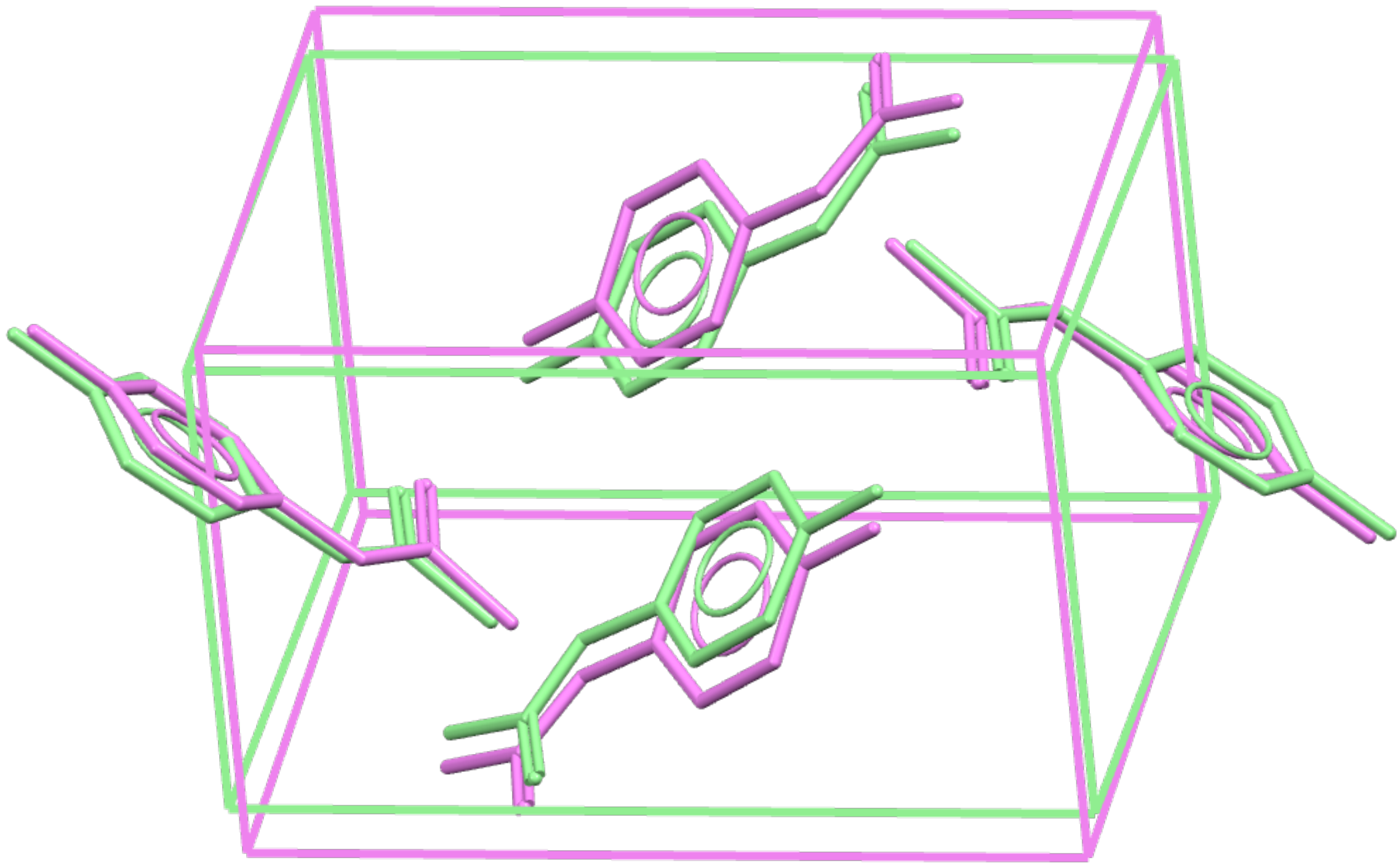

Figure S 5a. Overlay of the experimental crystal structures of Paracetamol at atmospheric pressure, *i.e*. at ≈0.0 GPa, (violet) and at 4.0 GPa (light green).

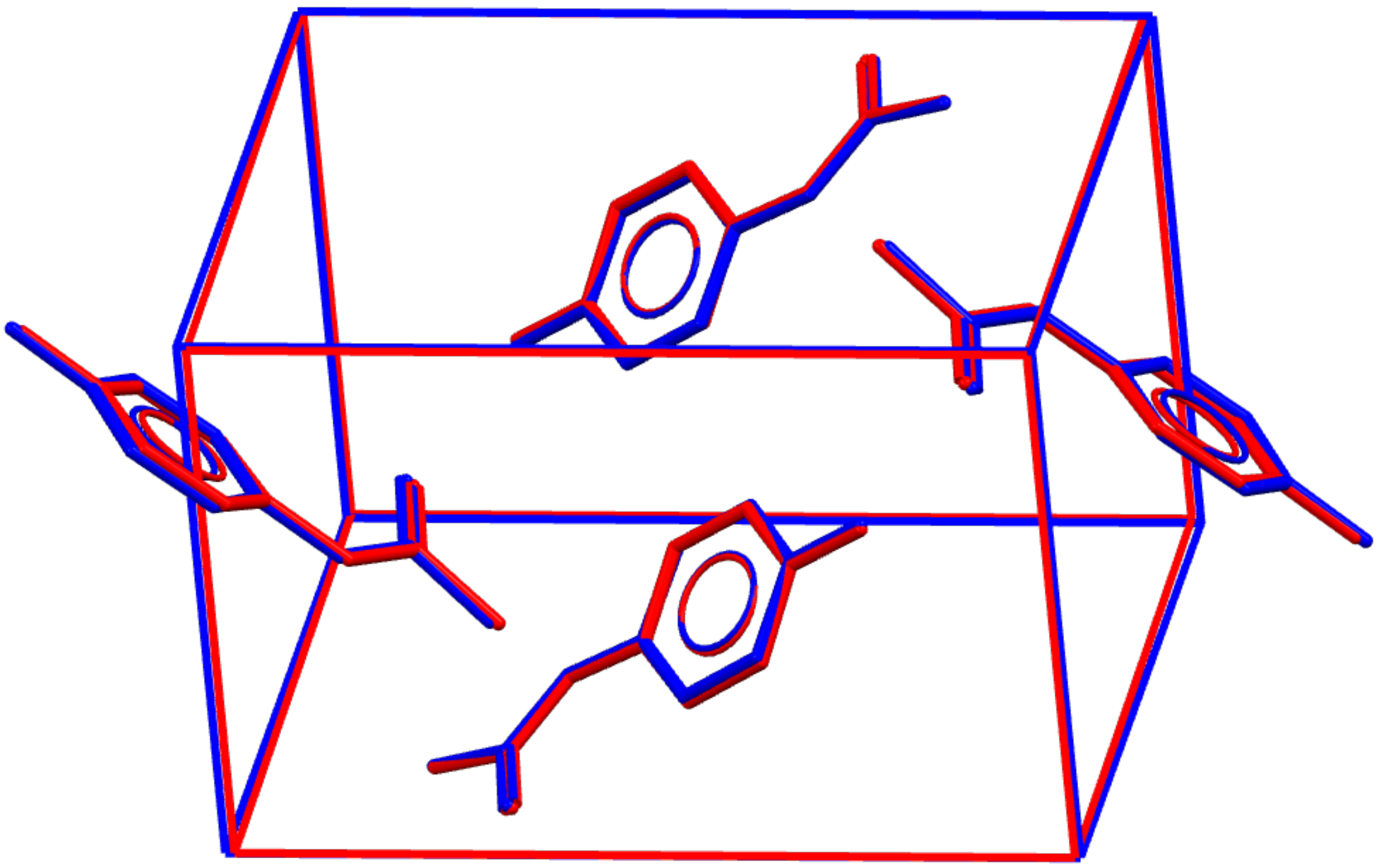

Figure S 5b. Overlay of the experimental crystal structure of Paracetamol at atmospheric pressure, *i.e*. at ≈0.0 GPa, (red) and the same crystal structure after energy minimisation without a pressure term, *i.e*. at 0.0 GPa, (blue). RMSCD = 0.078 Å.

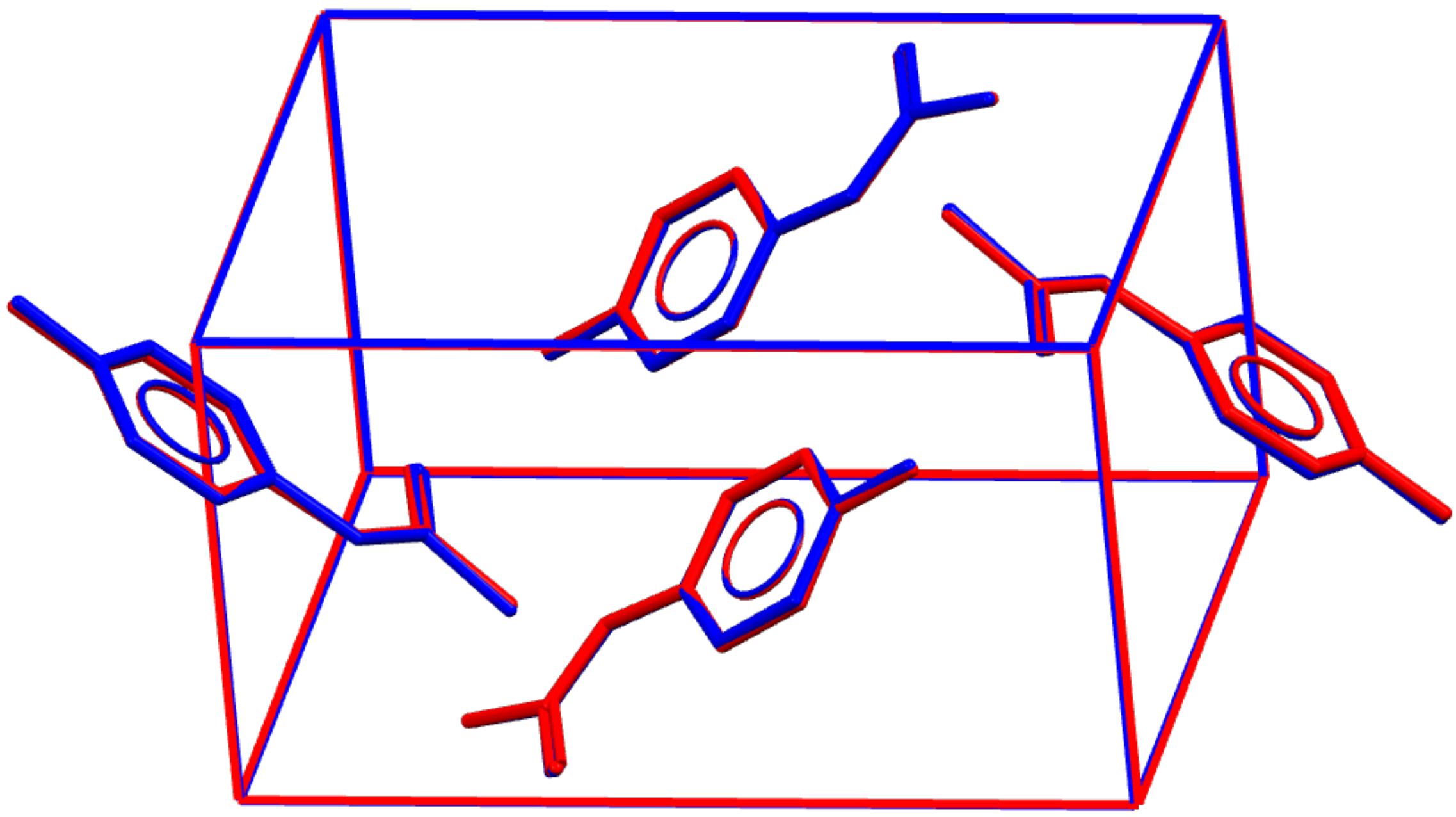

Figure S 5c. Overlay of the experimental crystal structure of Paracetamol at 4.0 GPa (red) and the same crystal structure after energy minimisation at 4.0 GPa (blue); RMSCD = 0.051 Å. Including the effect of hydrostatic pressure in DFT calculations is easy, fast and reliable.

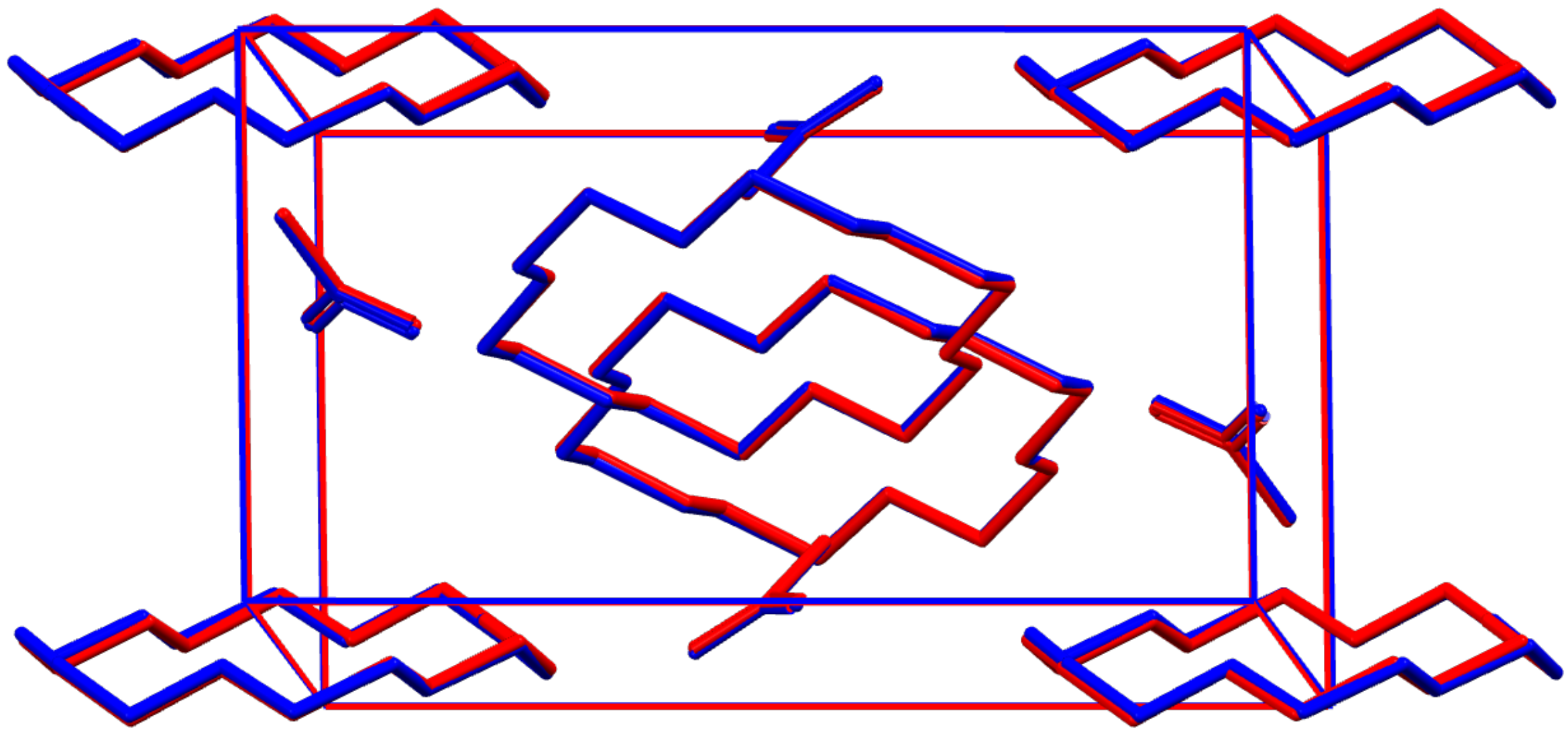

Figure S 6a. Overlay of the CSD reference code BIJWAS02, determined at 123 K (red) and the same crystal structure after energy minimisation (blue); RMSCD = 0.075 Å.

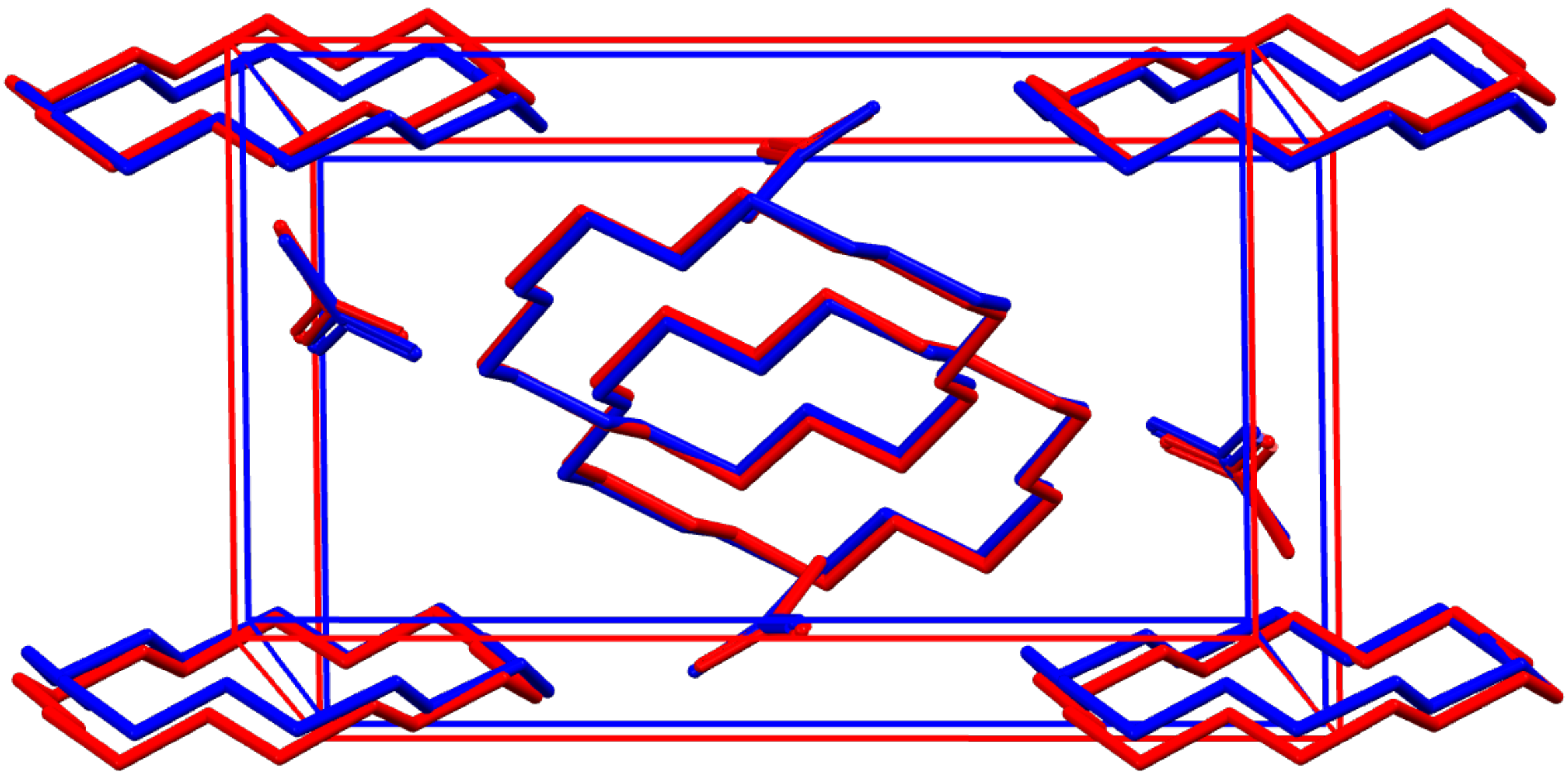

Figure S 6b. Overlay of the CSD reference code BIJWAS01, determined at room temperature (red) and the same crystal structure after energy minimisation (blue); RMSCD = 0.18 Å. The crystal structure exhibits untypically large thermal expansion.

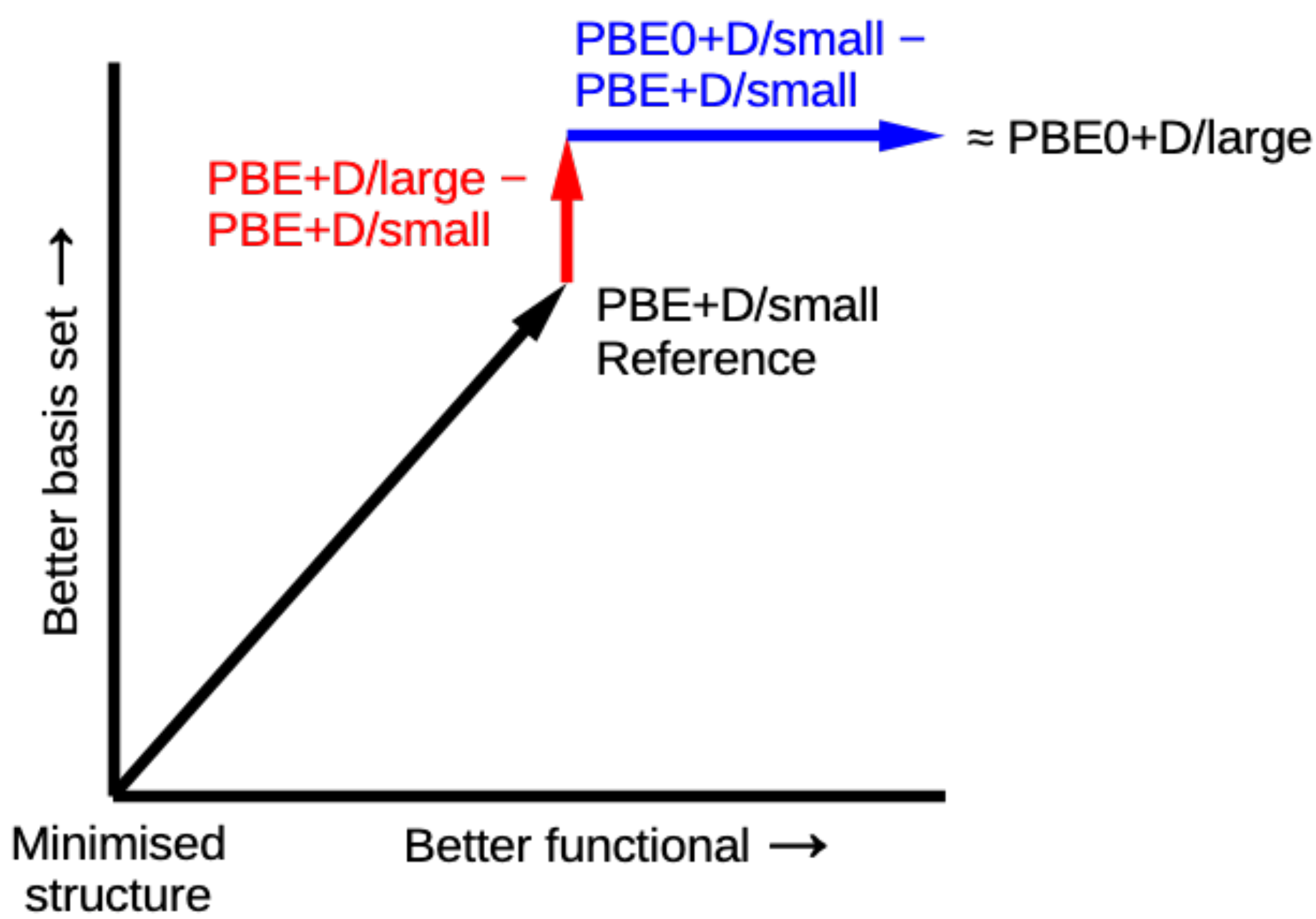

Figure S 7. Visualisation of Equation (3). If a single-point calculation with the PBE0 functional with the tight basis set is too expensive to run, it can be approximated by running a reference single-point calculation to which two single-point corrections are added, one to improve the functional and one to improve the basis set.

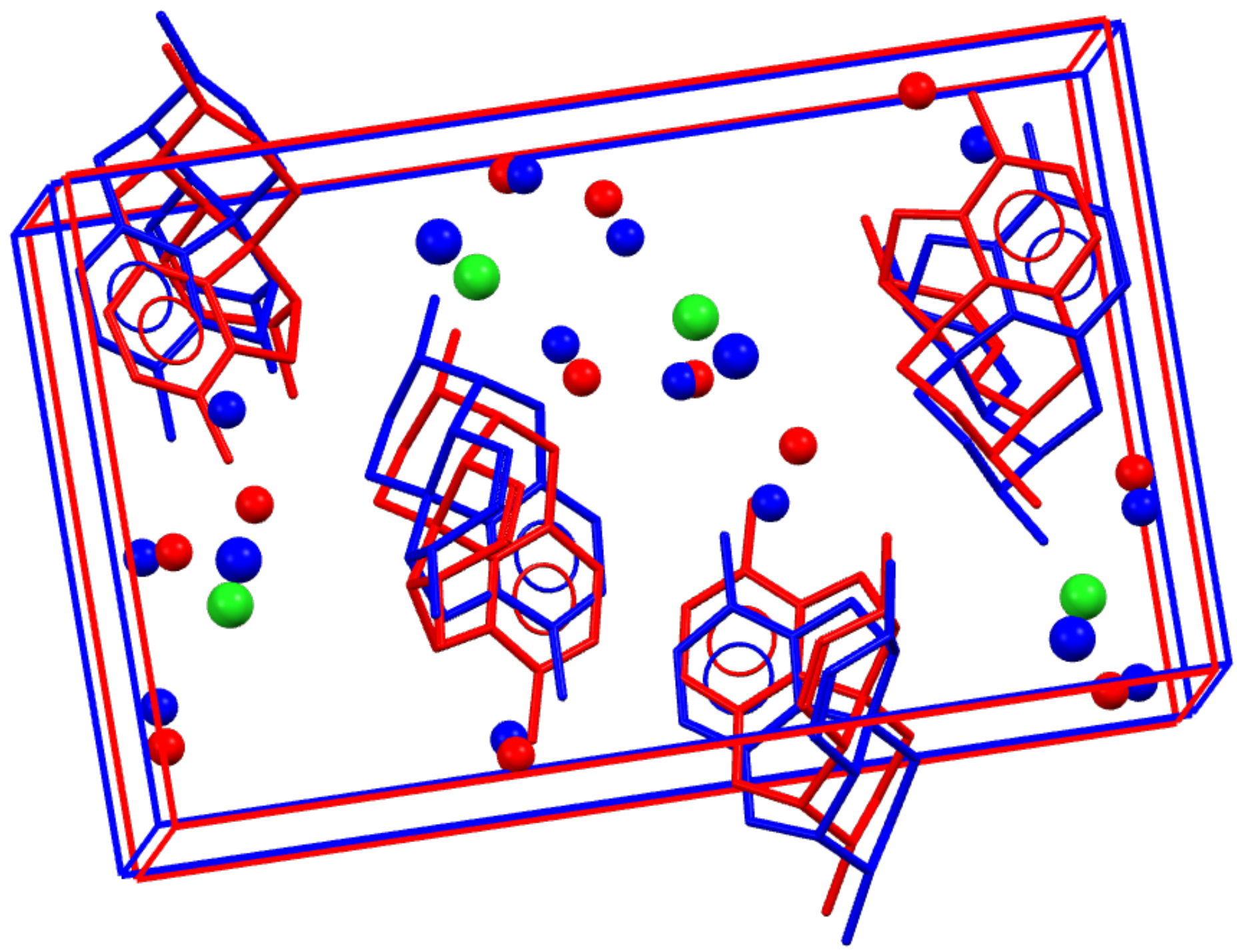

Figure S 8a. Overlay of the experimental crystal structure of Morphine HCl trihydrate (red) as published in 1973, and the same structure after energy minimisation (blue). The experimental position of the chloride anion is shown in green. RMSCD = 0.81 Å.

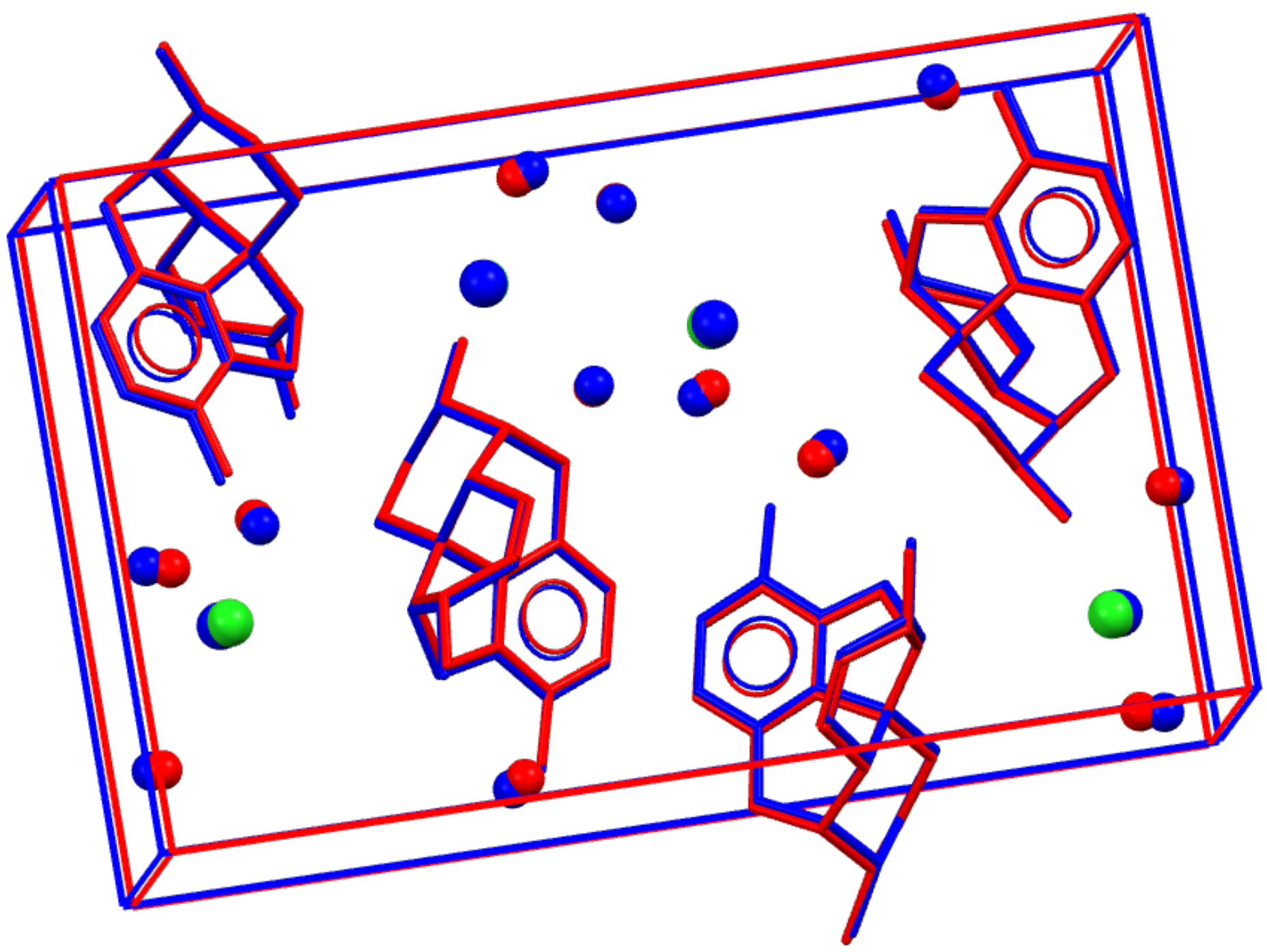

Figure S 8b. Overlay of the experimental crystal structure of Morphine HCl trihydrate after minor adjustments to some of the hydrogen atoms (red), and the same structure after energy minimisation (blue). The experimental position of the chloride anion is shown in green. RMSCD = 0.15 Å.

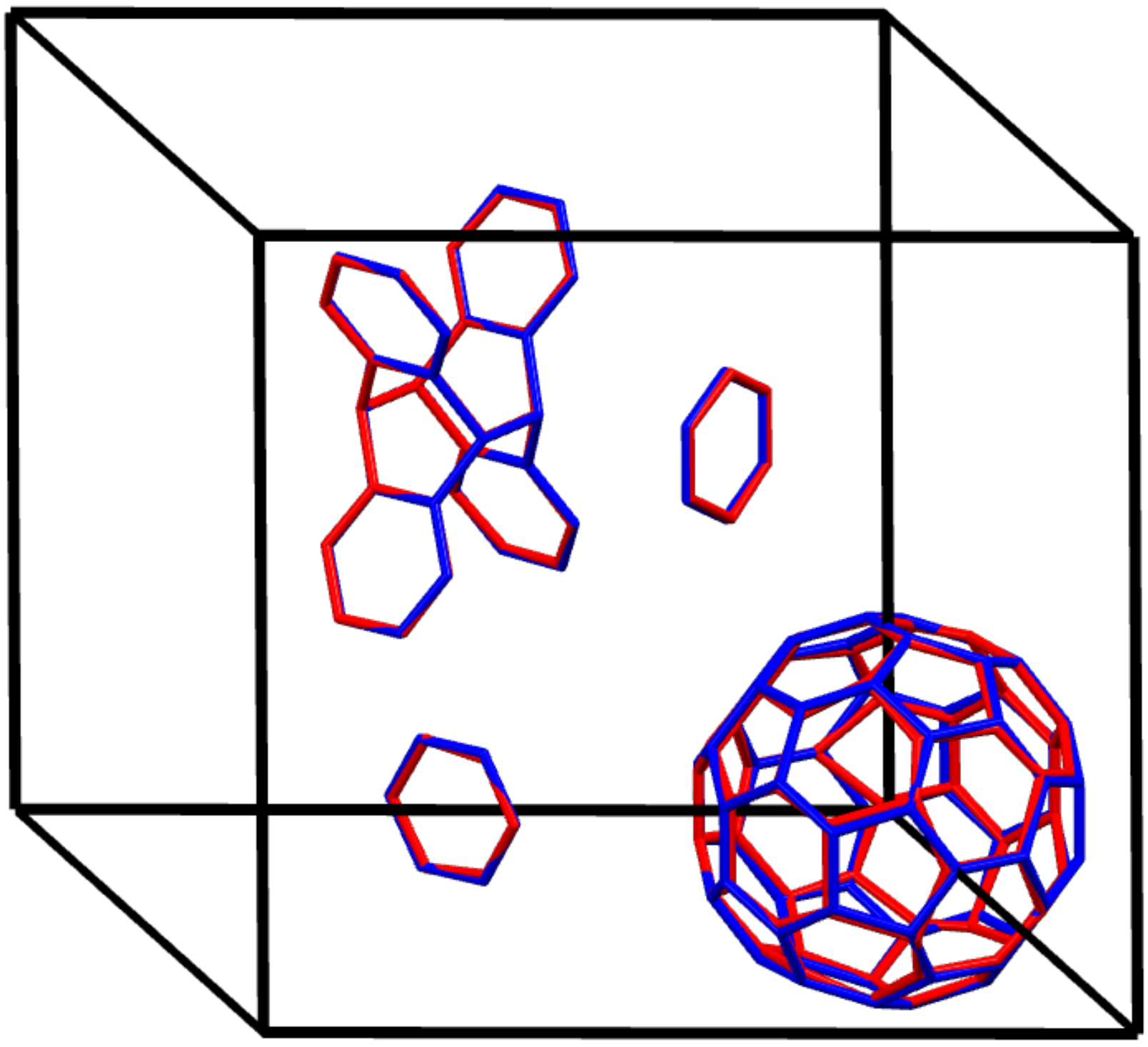

Figure S 9a. Overlay of CSD reference code PASQAB, refined with a missing centre of symmetry resulting in distortions of the molecular geometry (red) and the same structure after energy minimisation with the unit cell fixed (blue). RMSCD = 0.042 Å.

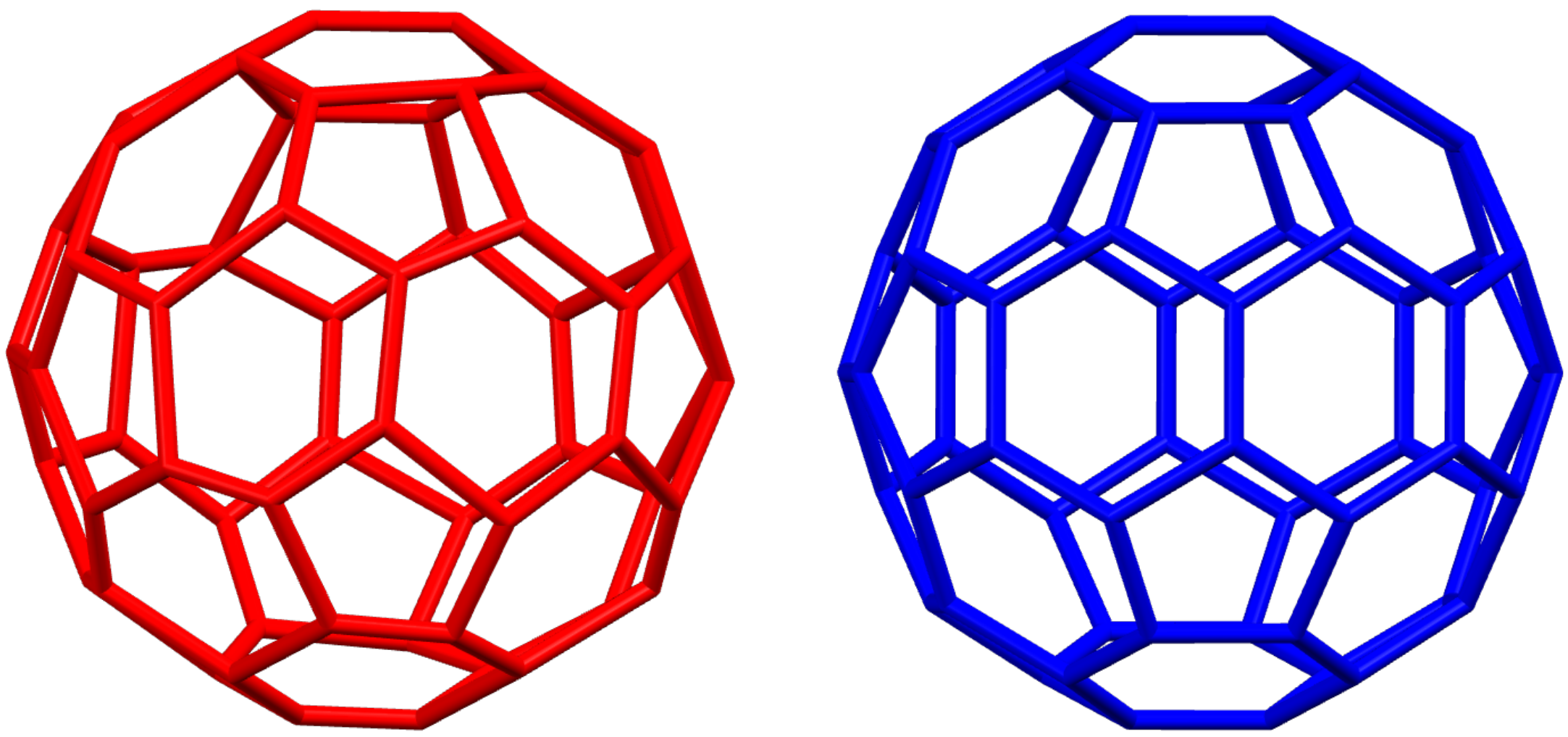

Figure S 9b. Molecular geometry from the crystal structure of CSD reference code PASQAB as refined and published in space group *Cm*, *i.e*. with a missing centre of symmetry (red) and after energy minimisation of the crystal structure in *Cm* (blue).

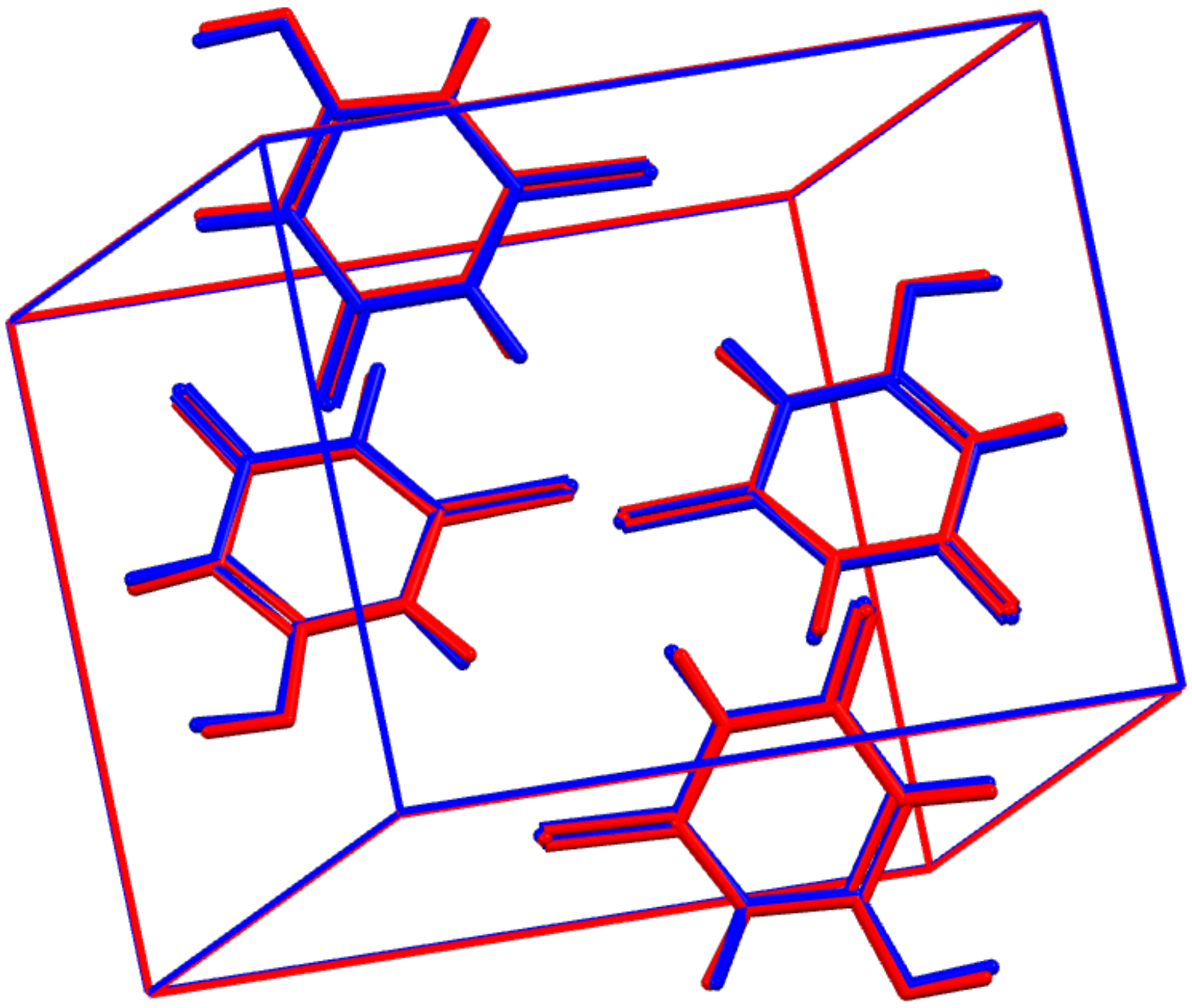

Figure S 10a. Overlay of the experimental crystal structure of the enol-only tautomer of Thiobarbituric acid (PABNAJ, Form II, red) and the same crystal structure after energy minimisation (blue). RMSCD = 0.070 Å.

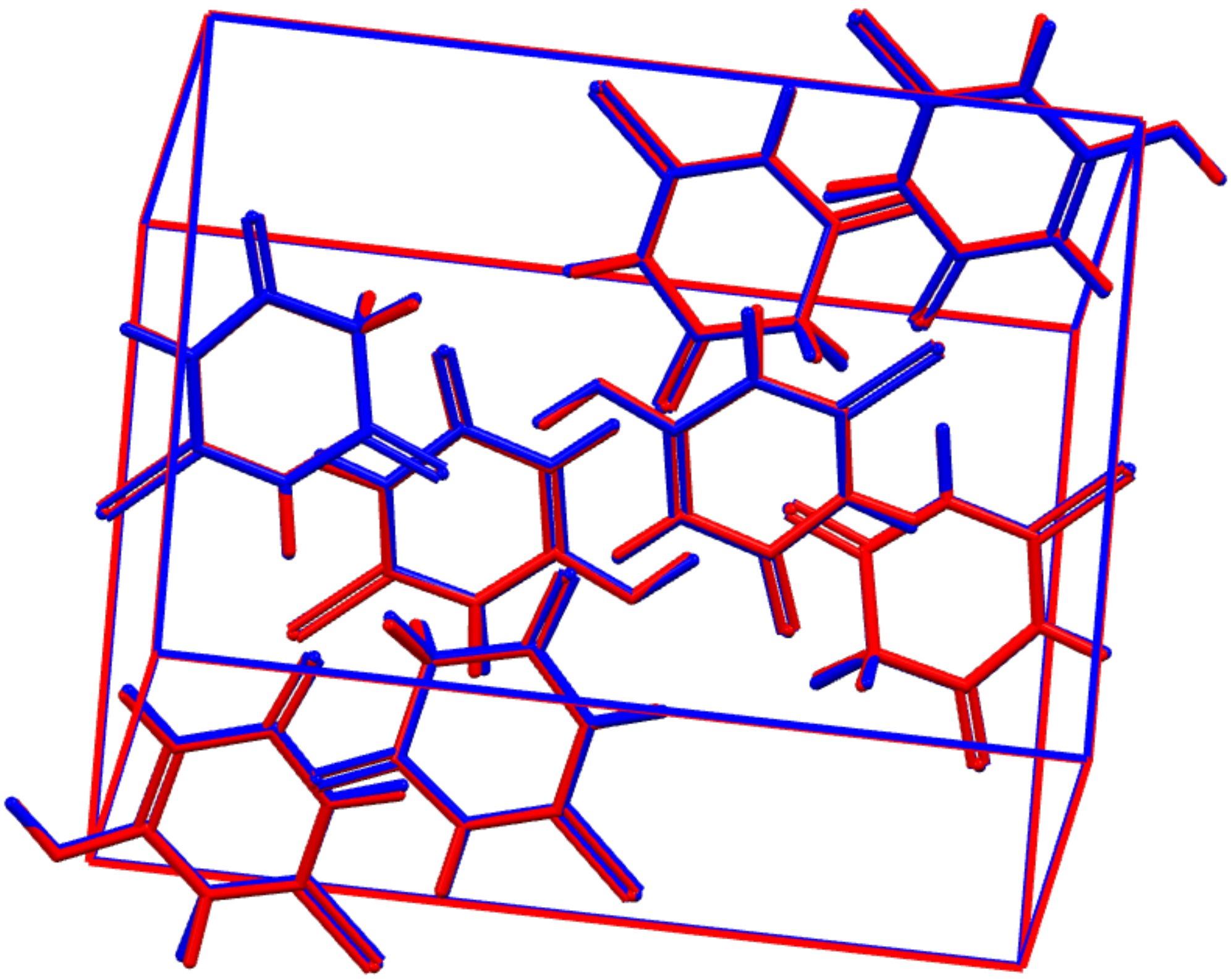

Figure S 10b. Overlay of the experimental crystal structure of the enol + keto tautomers of Thiobarbituric acid (PANBIR, Form IV, red) and the same crystal structure after energy minimisation (blue). RMSCD = 0.039 Å.

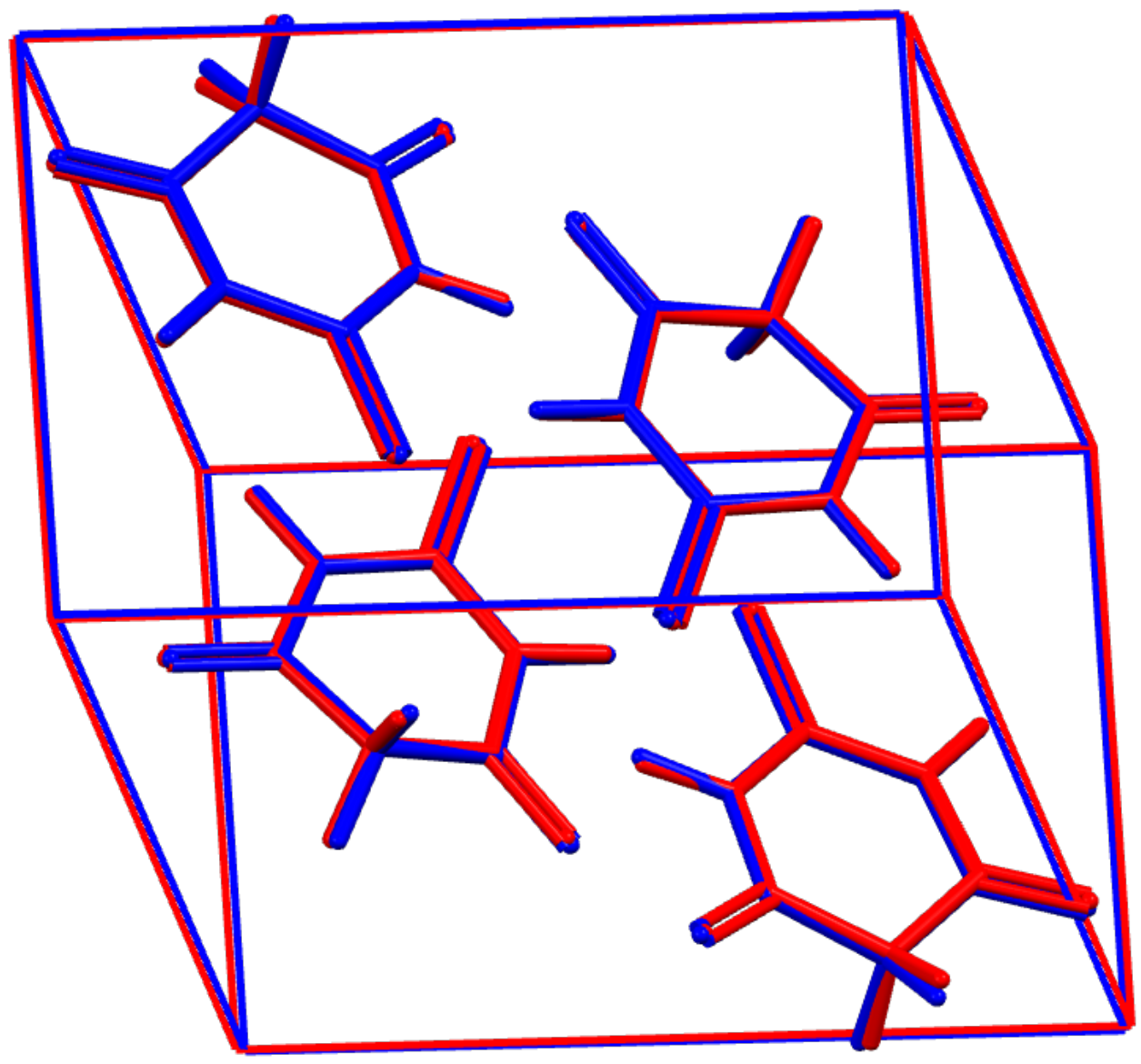

Figure S 10c. Overlay of the experimental crystal structure of the keto-only tautomer of Thiobarbituric acid (THBARB01, Form III, red) and the same crystal structure after energy minimisation (blue). RMSCD = 0.035 Å.

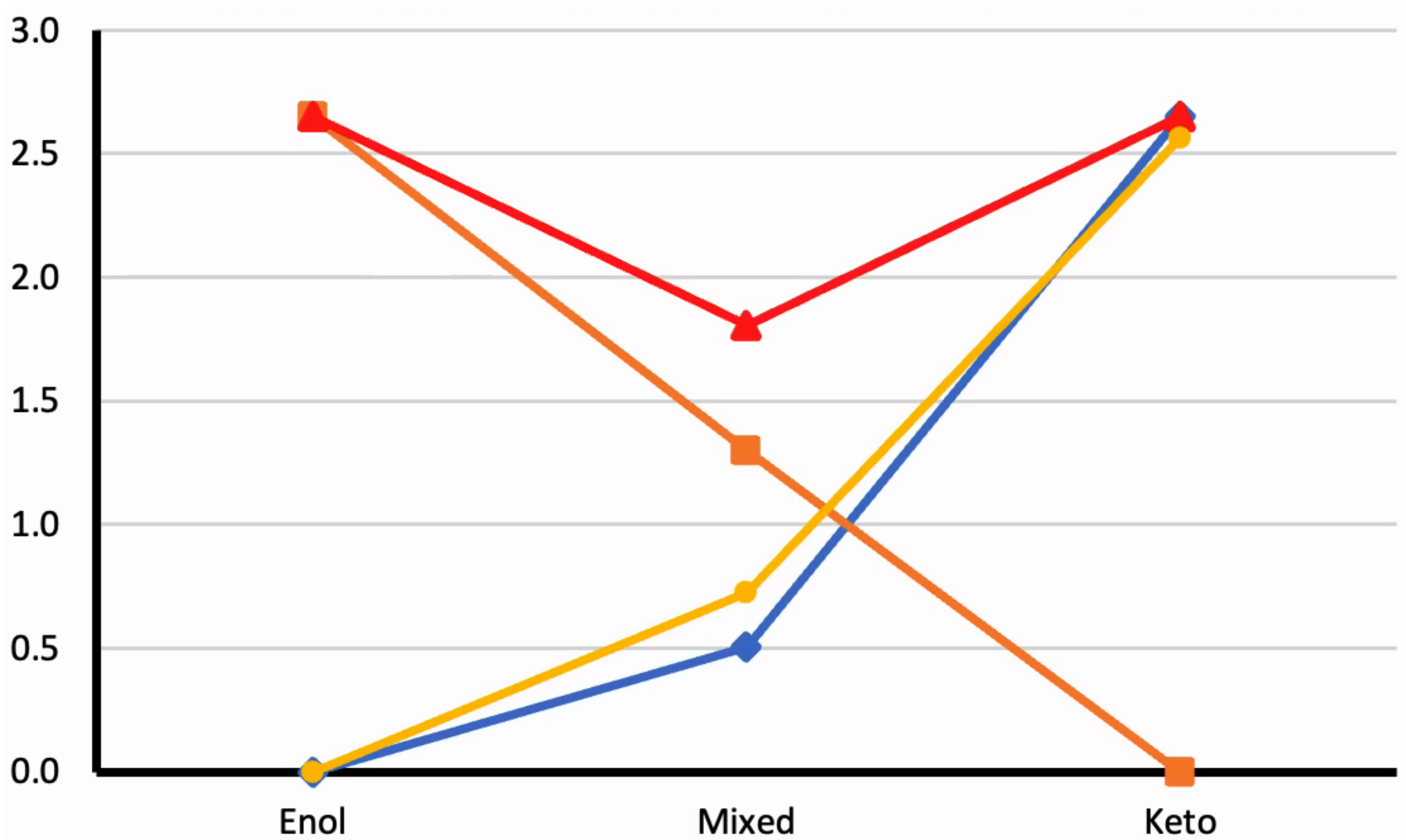

Figure S 11. PBE+D/lightdense crystal energy (yellow circles), PBE0+D/tight single-point crystal energy (blue diamonds), ωB97X+D single-point conformer energy correction (orange squares) and PBE0+D/tight & ωB97X+D crystal energy (red triangles) of three polymorphs of Thiobarbituric acid containing different tautomers. The mixed polymorph, containing equal amounts of the enol and the keto tautomer, is experimentally the most stable form.

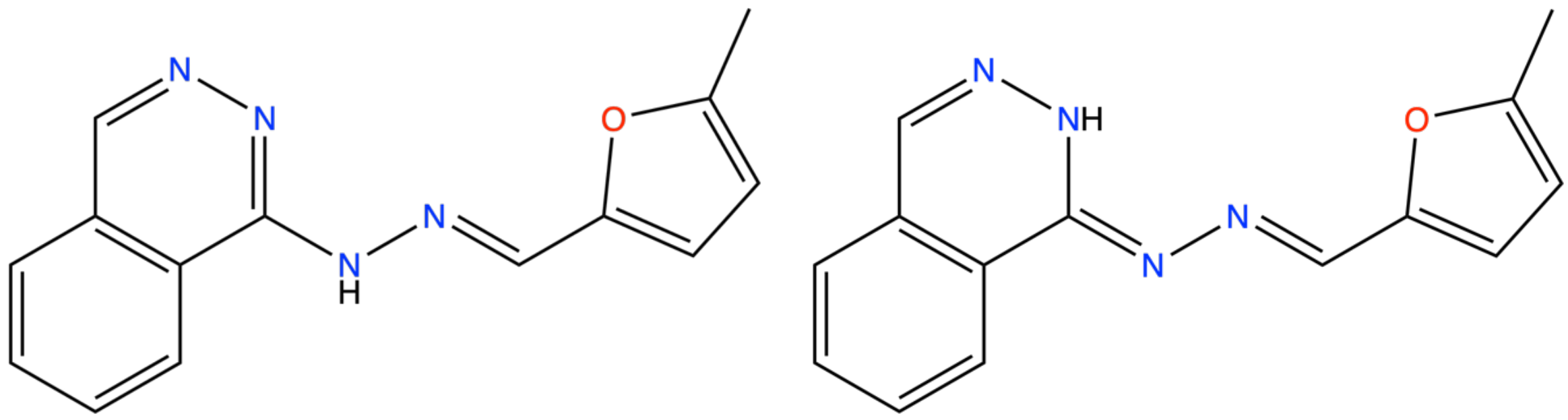

Figure S 12. The 2D chemical diagram of CSD reference code NILVEL as published (left) and a tautomer (right).

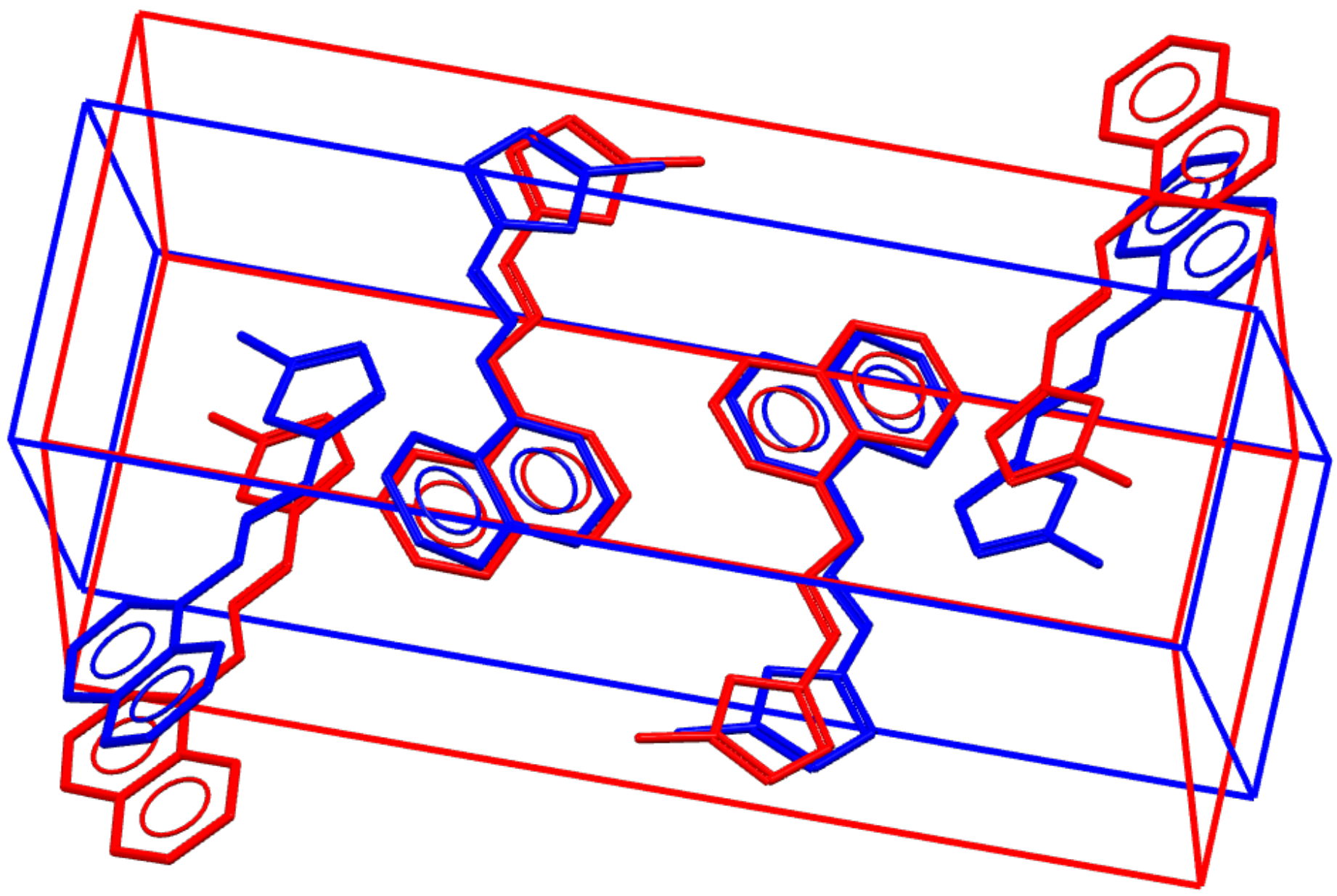

Figure S 13a. Overlay of the experimental crystal structure of CSD reference code NILVEL as published (red) and the same structure after energy minimisation (blue). The distortion of the structure upon energy minimisation suggests a problem with the experimental structure. RMSCD = 0.84 Å.

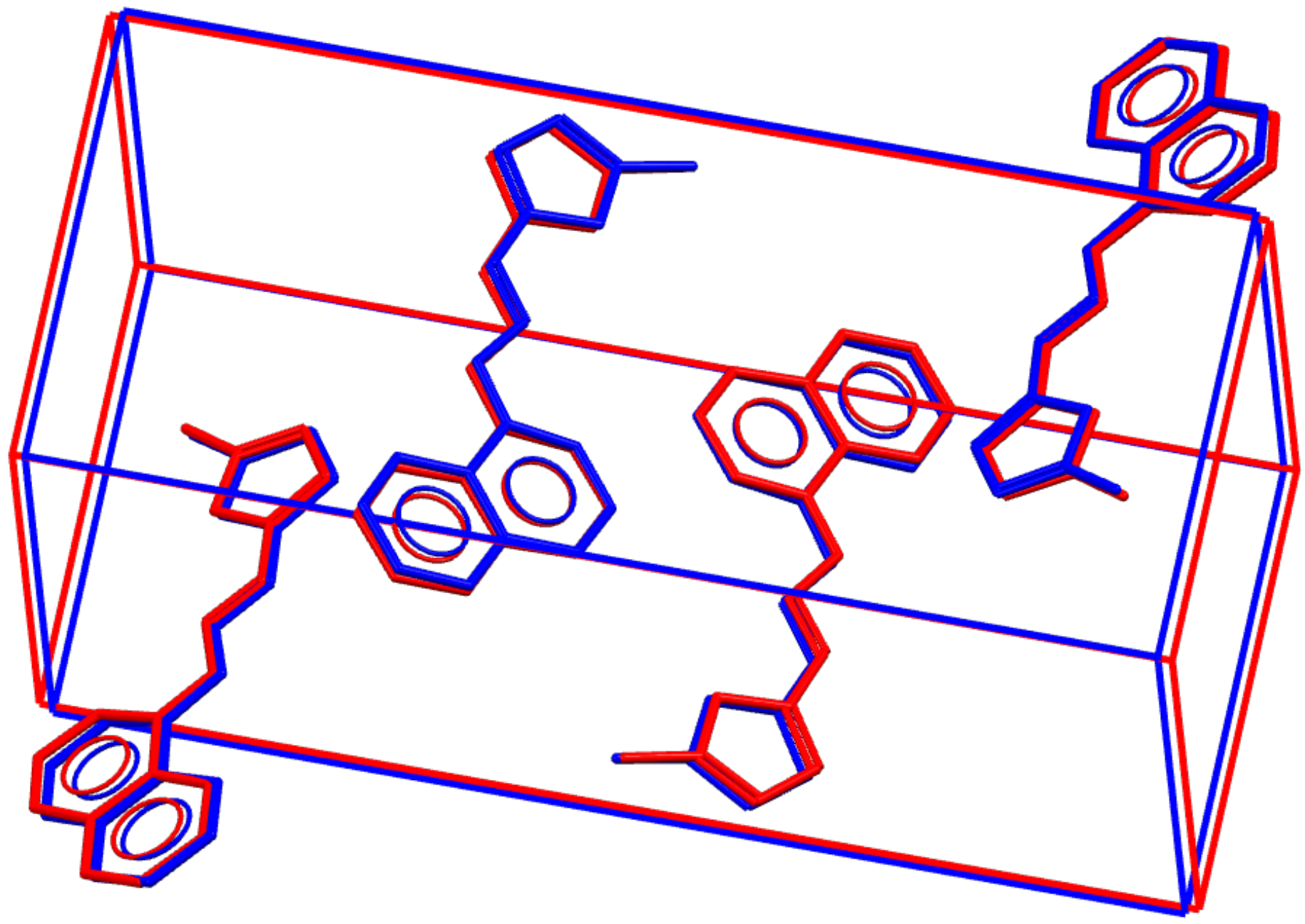

Figure S 13b. Overlay of the experimental crystal structure of CSD reference code NILVEL with a reinterpretation of the tautomer (red) and the same structure after energy minimisation (blue). RMSCD = 0.11 Å.

Example Paroxetine HCl 0.9 hydrate.

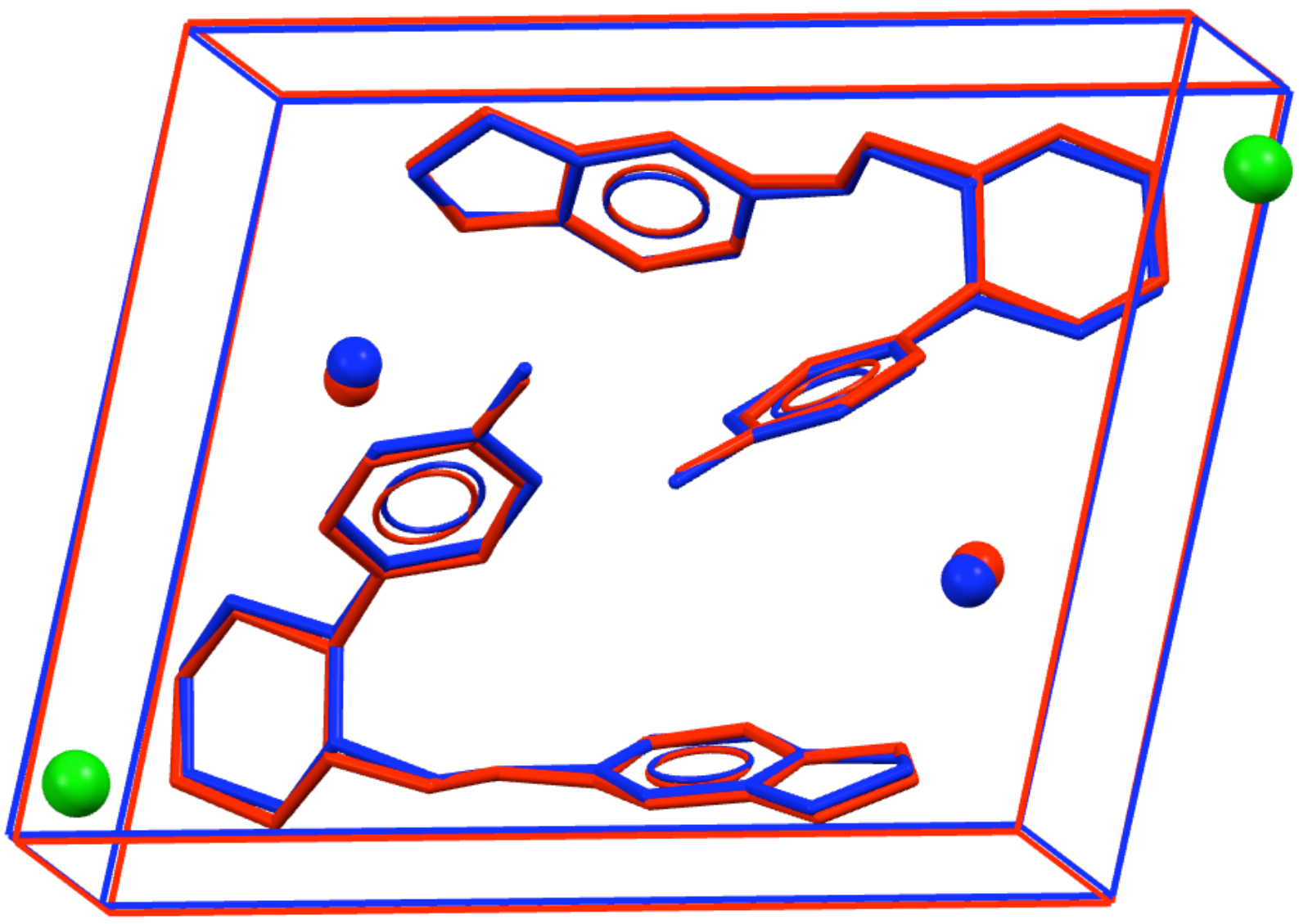

Figure S 14. Overlay of the experimental crystal structure of Paroxetine HCl 0.9 hydrate (red), determined from powder diffraction data, and the same structure after energy minimisation (blue); RMSCD = 0.20 Å. The chloride anion, which hardly moves upon energy minimisation, is shown in green.

Example Glipizide.

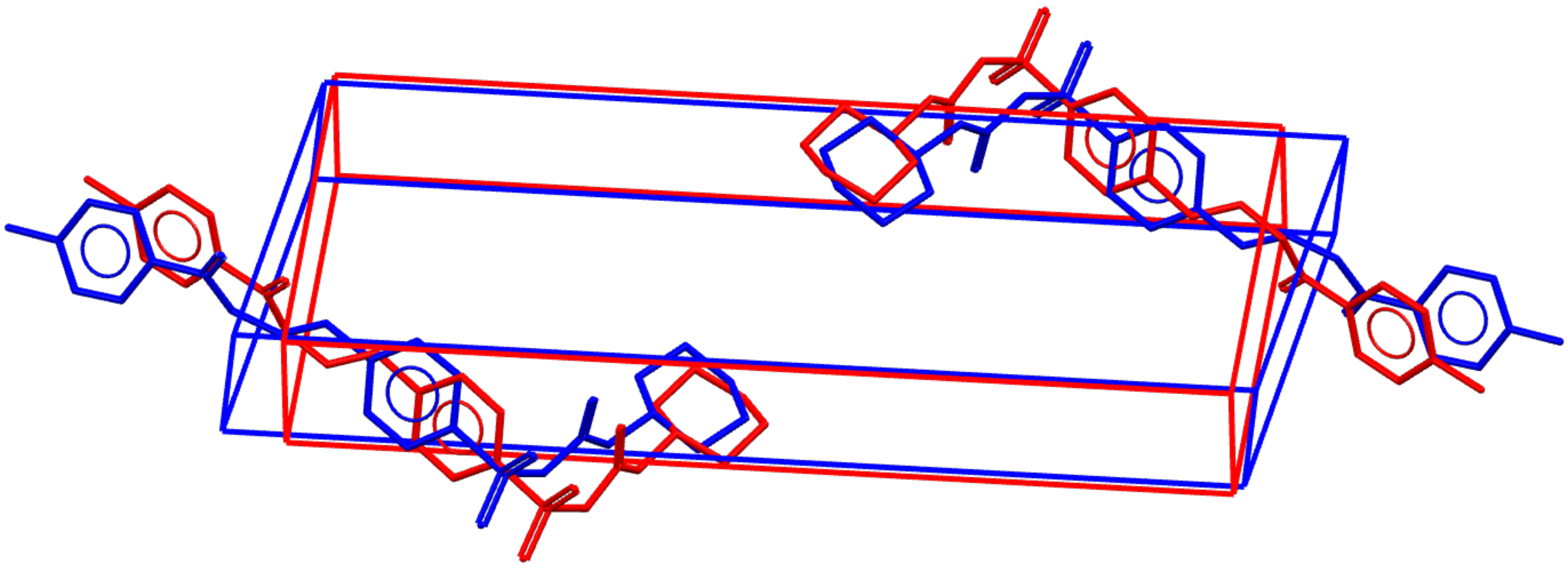

Figure S 15a. Overlay of the crystal structure of Glipizide, determined from powder diffraction data, as published (red) and the same structure after energy minimisation (blue); RMSCD = 0.72 Å. The distortion of the structure upon energy minimisation suggests a problem with the experimental structure.

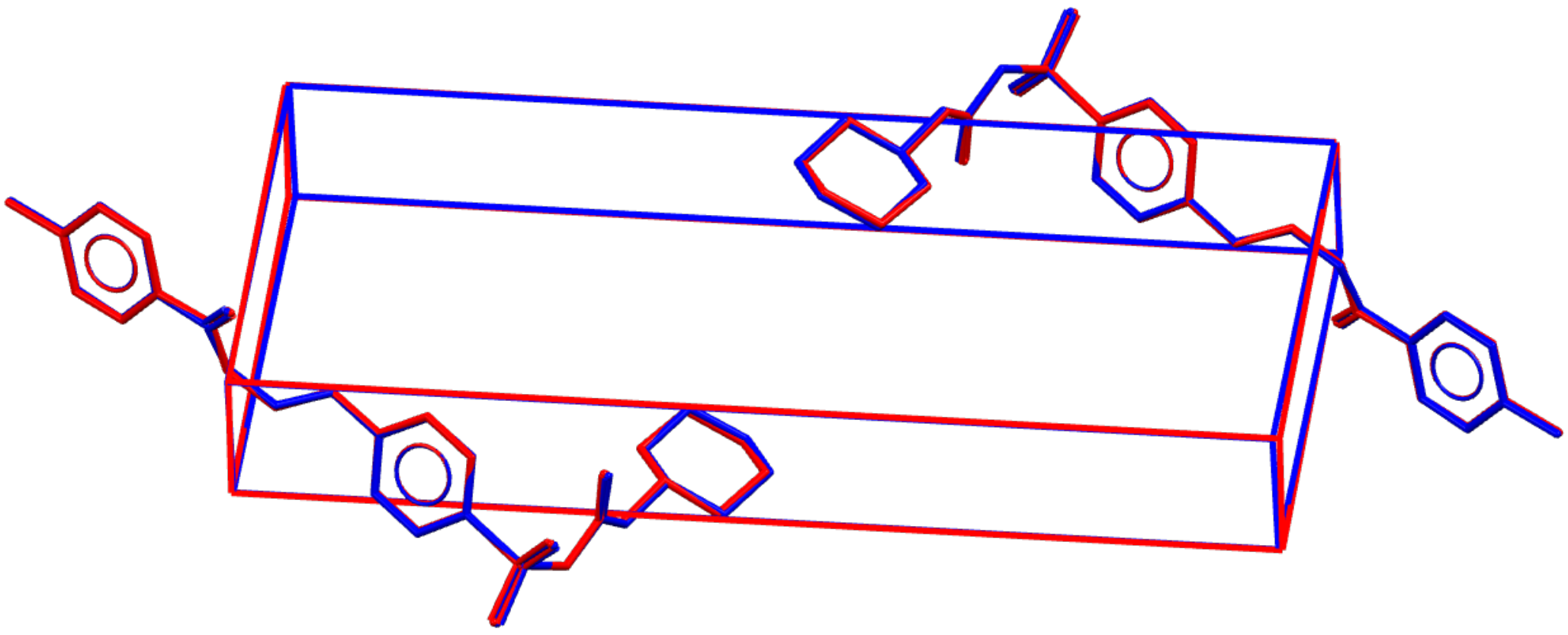

Figure S 15b. Overlay of the corrected crystal structure of Glipizide (red) and the same structure after energy minimisation (blue); RMSCD = 0.13 Å.

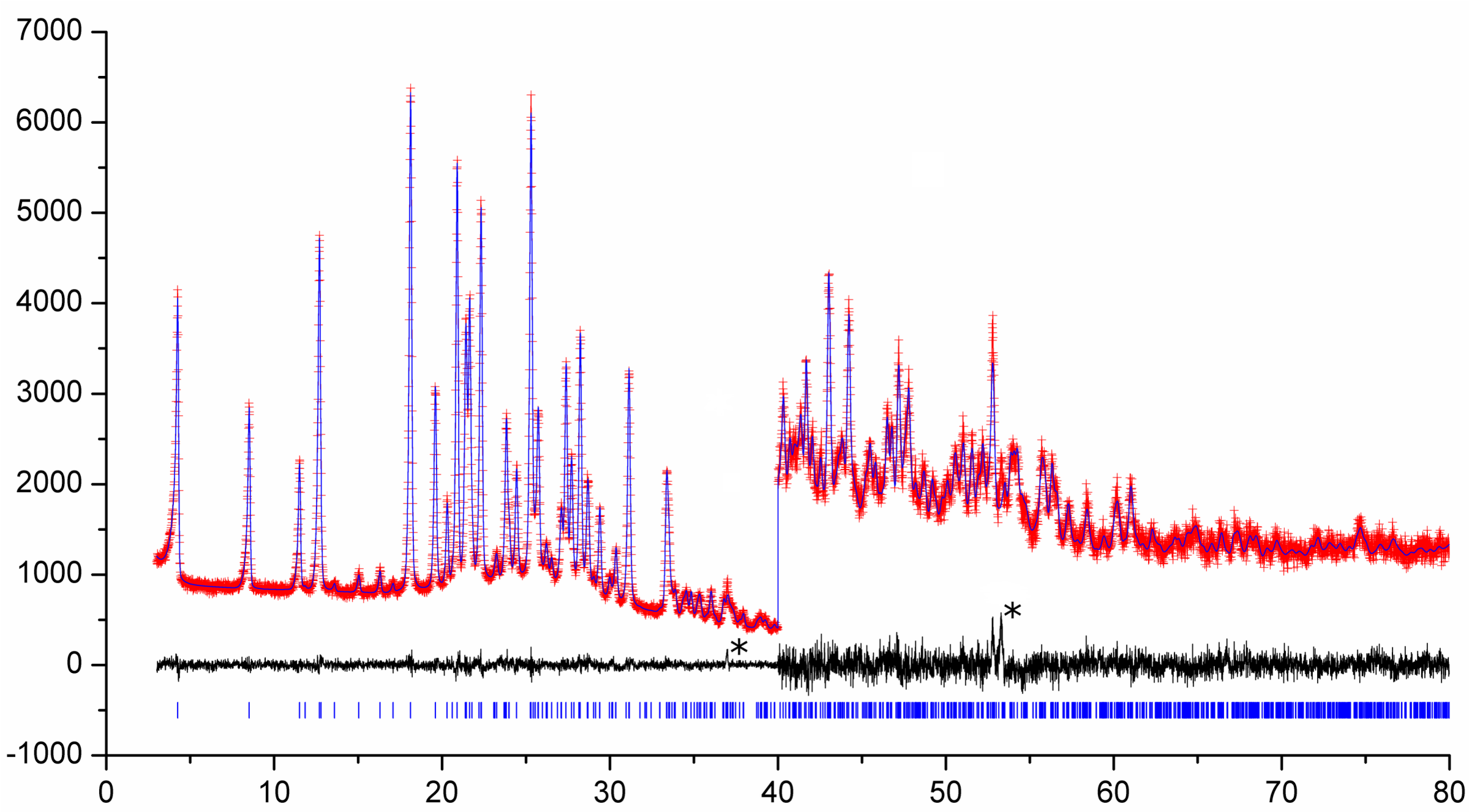

Figure S 16. Rietveld refinement of the corrected model of Glipizide against the published experimental data using polymorph-dependent restraints from a DFT energy minimisation. The two peaks indicated by asterisks correspond to NaCl. Intensities above 40° 2θ multiplied by five. Experimental data (red), Rietveld fit (blue) and difference curve (black) are shown. *x*-axis: 2θ, *y*-axis: counts.

Example Riboflavin.

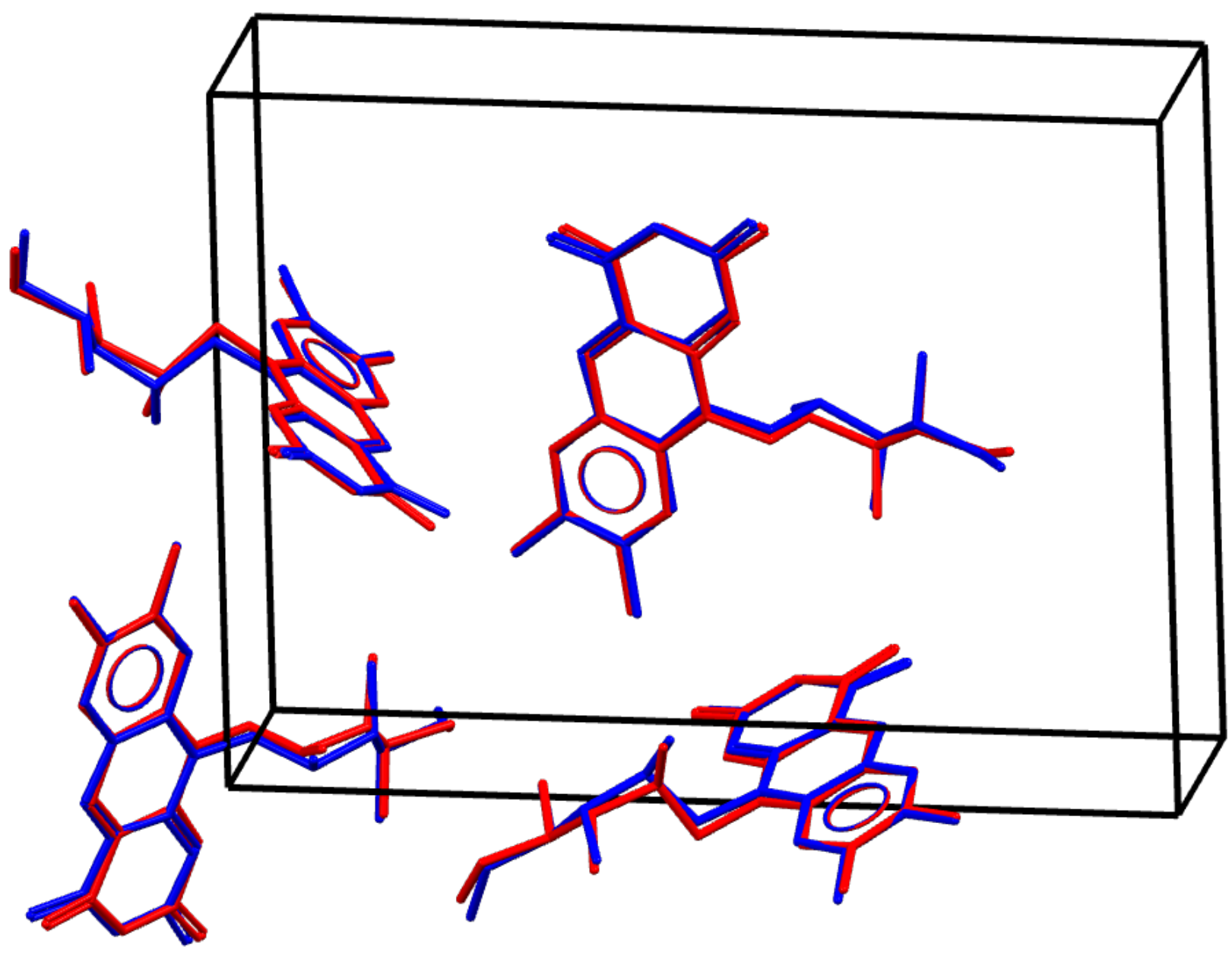

Figure S 17a. Overlay of the crystal structure of Riboflavin, determined from powder diffraction, as published (red) and the same structure after energy minimisation with the unit cell fixed (blue); RMSCD = 0.23 Å. By keeping the unit cell fixed, the structure is not given the space to distort, hiding the problem in the crystal structure.

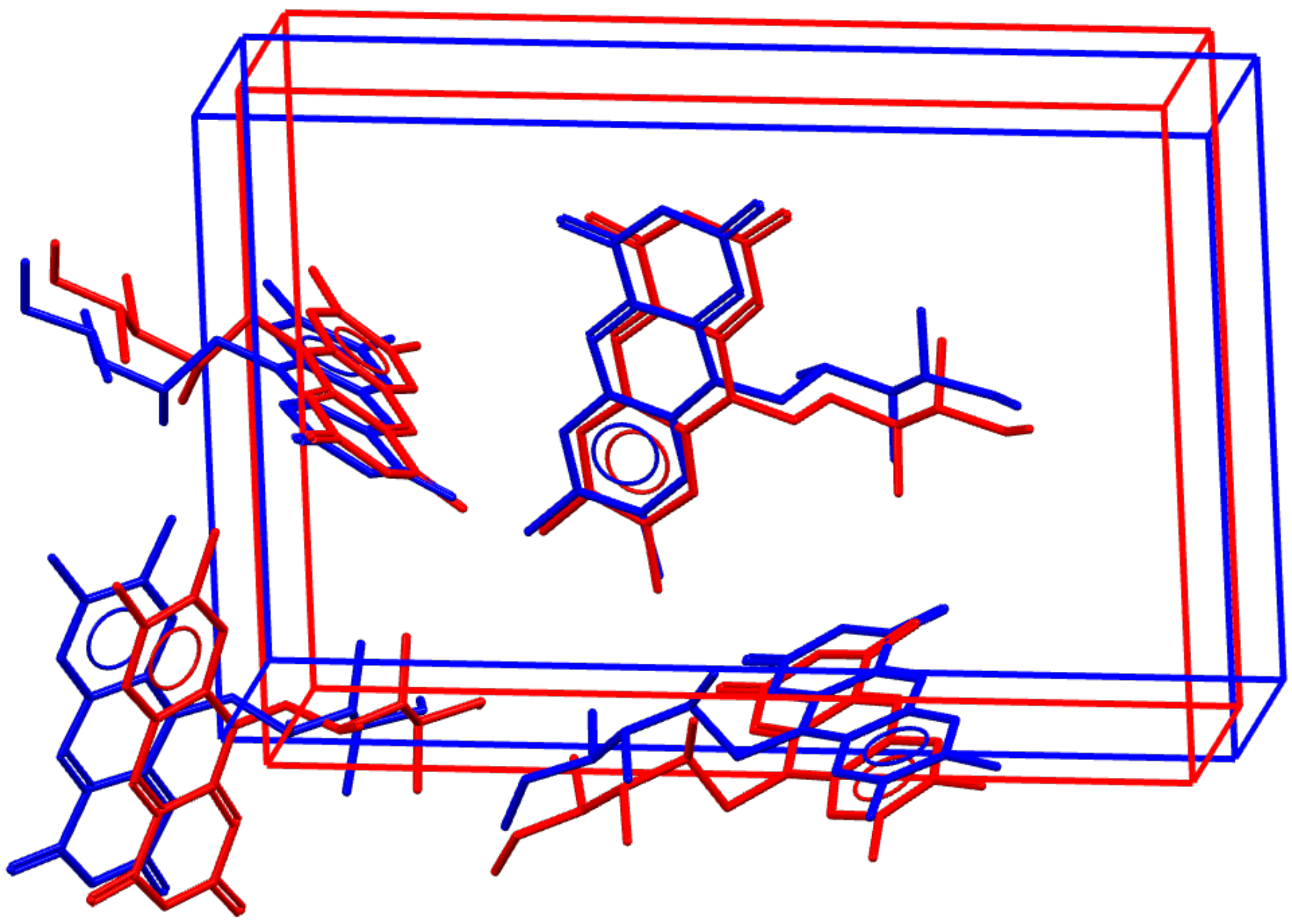

Figure S 17b. Overlay of the crystal structure of Riboflavin, determined from powder diffraction, as published (red) and the same structure after energy minimisation with the unit cell free (blue); RMSCD = 0.54 Å. The distortion of the structure upon energy minimisation suggests a problem with the experimental structure.

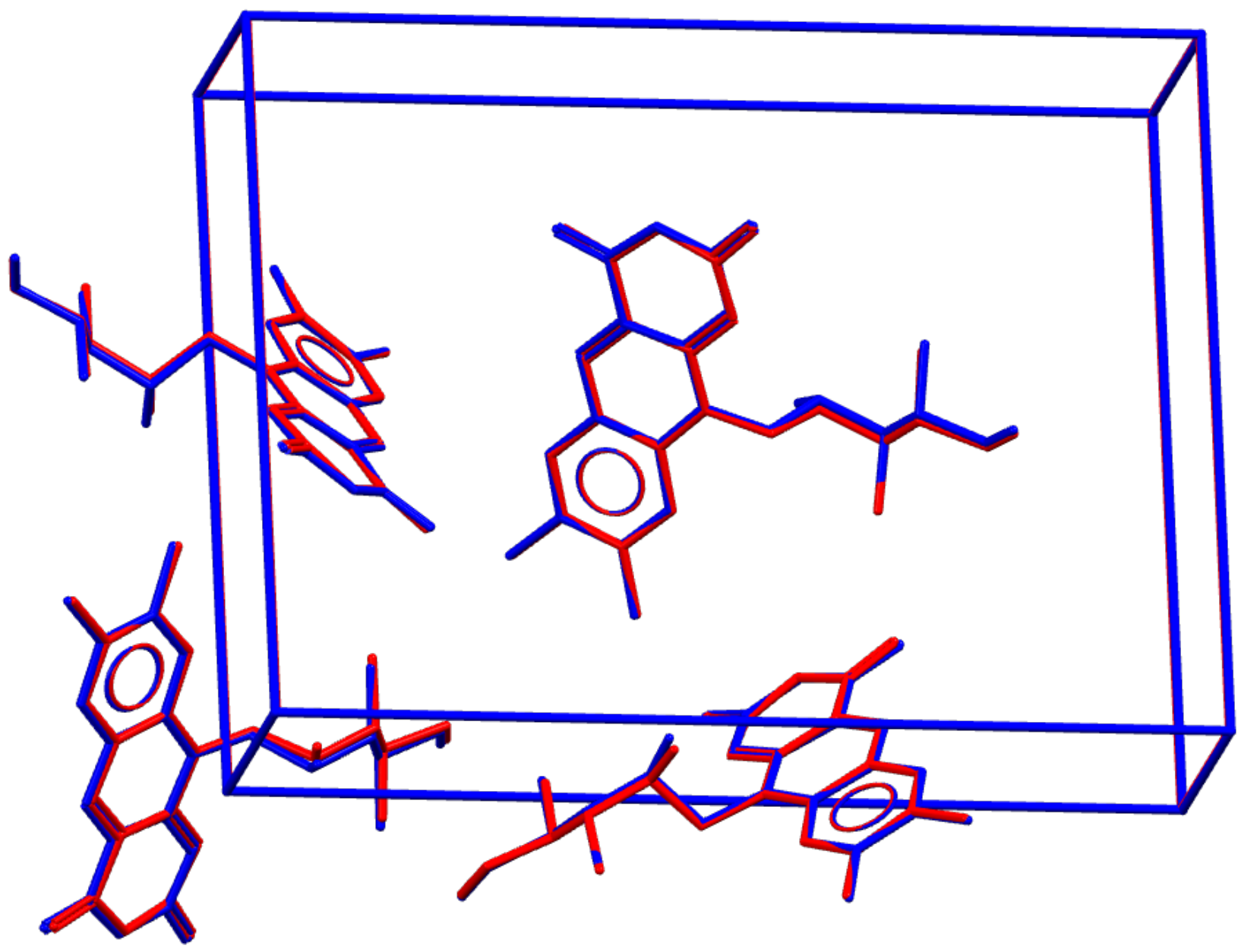

Figure S 17c. Overlay of the crystal structure of Riboflavin with an alternative hydrogen-bonding pattern, Rietveld refined against the original powder diffraction data (red) and the same structure after energy minimisation with the unit cell free (blue); RMSCD = 0.13 Å.

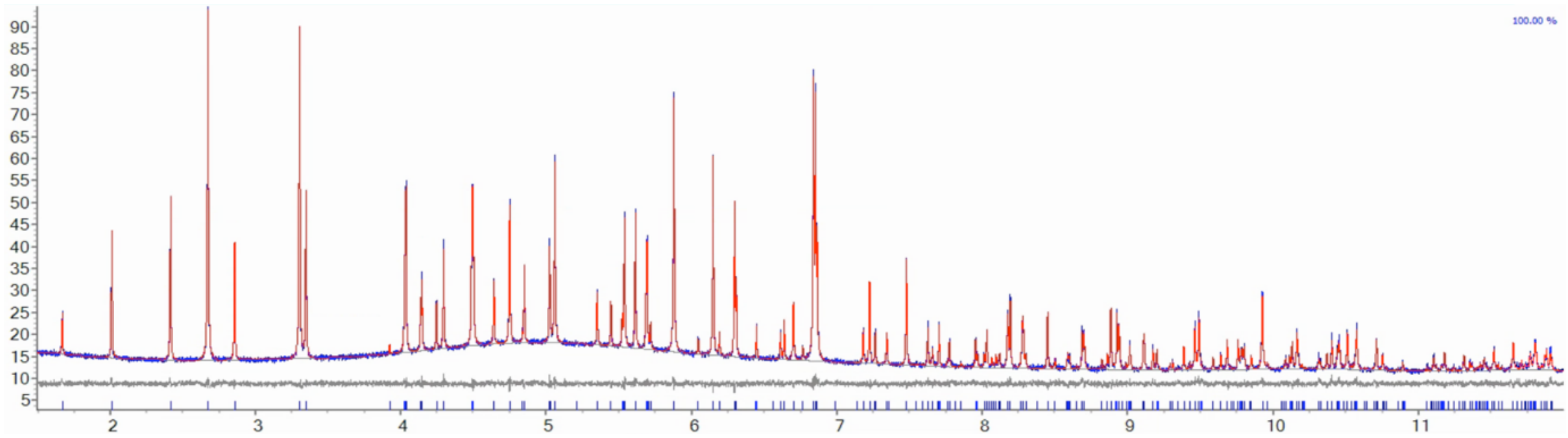

Figure S 18. Rietveld refinement of the corrected model of Riboflavin against the published experimental data using polymorph-dependent restraints from a DFT energy minimisation. Experimental data (blue), Rietveld fit (red) and difference curve (black) are shown. *x*-axis: 2θ, *y*-axis: sqrt(counts).
